# Supplementary figures and images for: Conifer-killing bark beetles locate fungal symbionts by detecting volatile fungal metabolites of host tree resin monoterpenes
Source: PLoS Biol. 2023 Feb 21;21(2):e3001887. doi: 10.1371/journal.pbio.3001887 (PMC9943021; doi:10.1371/journal.pbio.3001887)

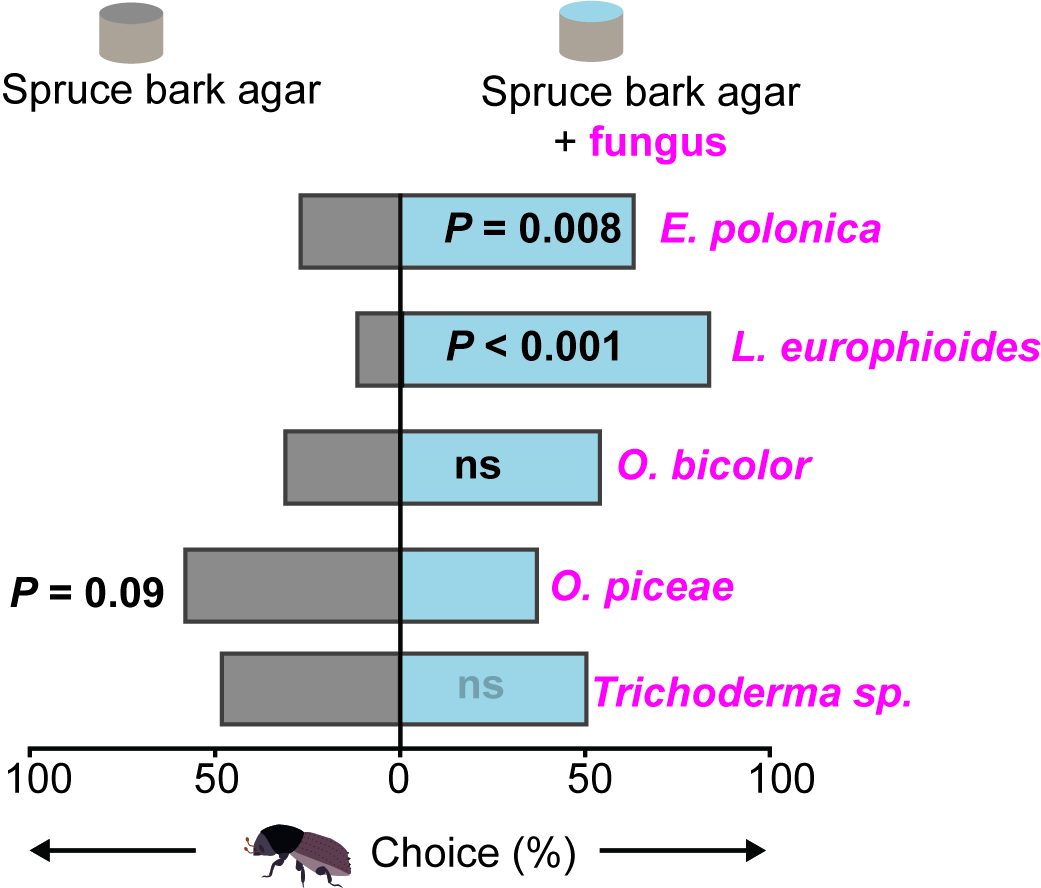

Supplement: S1 Fig — Adult beetles did not prefer O. bicolor, O. piceae, and Trichoderma sp., the latter two species are saprophytes. Deviation of response indices against zero was tested using Wilcoxon’s test (n = 20 or 25). The data underlying this Figure can be found at https://doi.org/10.6084/m9.figshare.21692156.v1. (TIF) [file pbio.3001887.s001.tif]

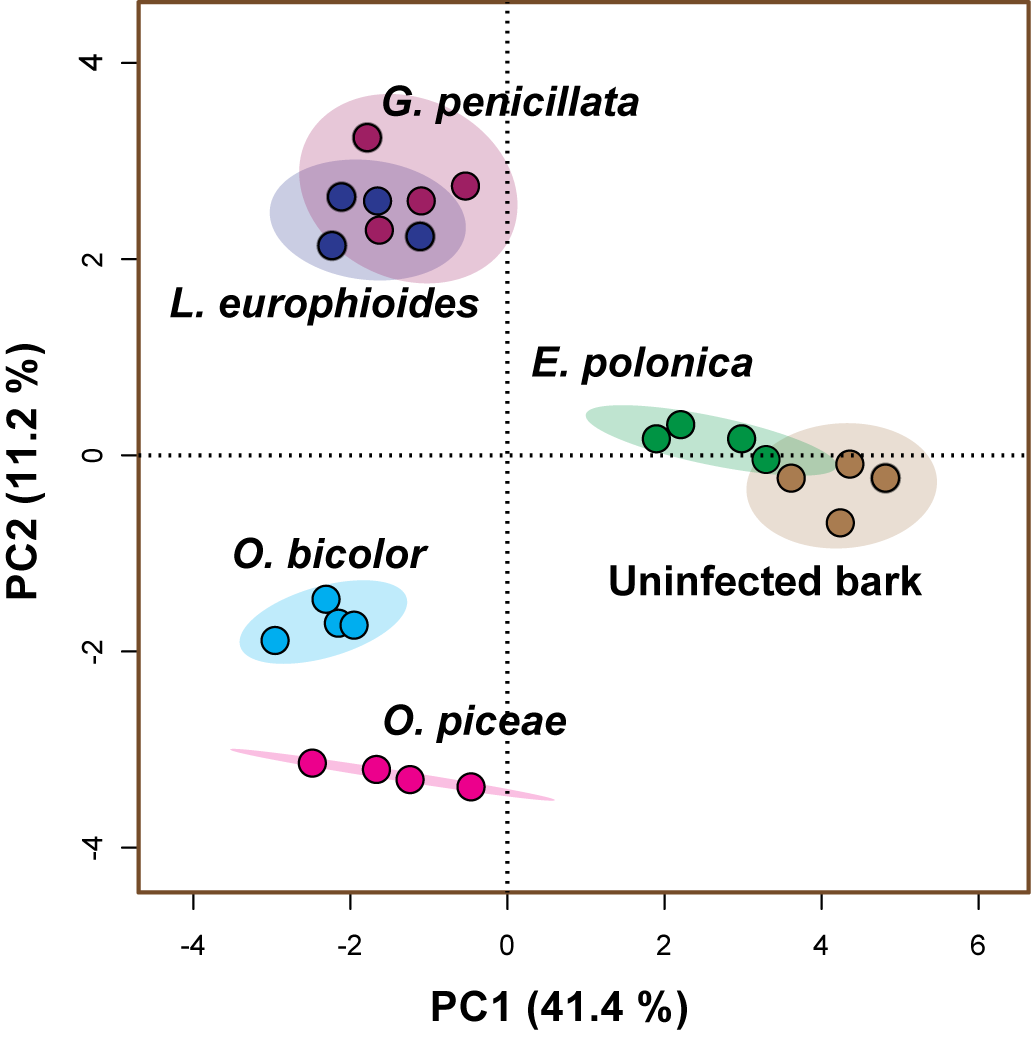

Supplement: S2 Fig — Analysis was performed using 59 compounds listed in S2 Table. Principal components (PC1 and PC2) explain 41.4% and 11.2% of the total variation, respectively, and ellipses denote 95% confident intervals around each species. The sPLS-DA plot was generated by using MetaboAnalyst 3.0 software with normalized data (both log transformed and range scaled). The data underlying this Figure can be found at https://doi.org/10.6084/m9.figshare.21692156.v1. (TIF) [file pbio.3001887.s002.tif]

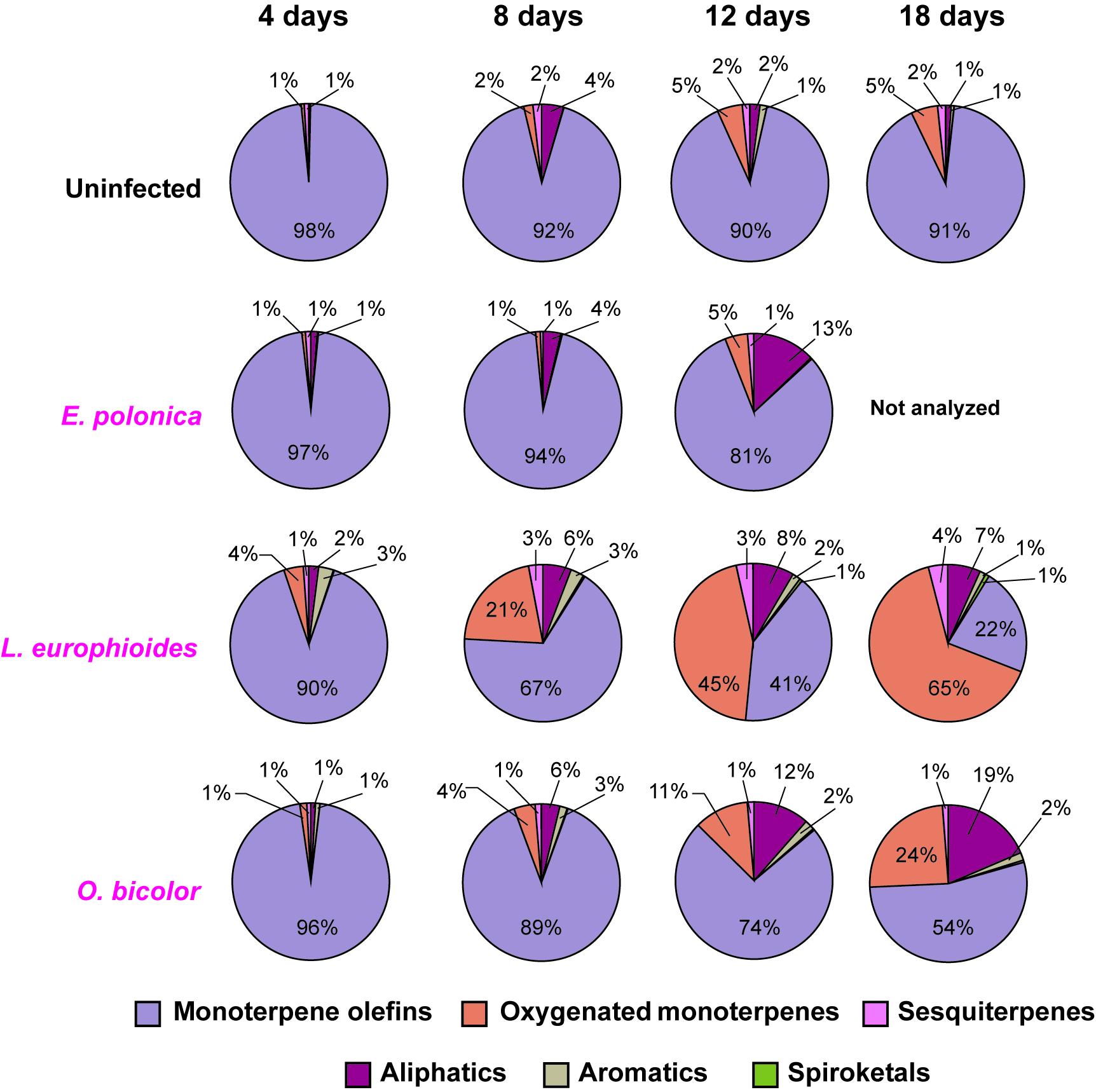

Supplement: S3 Fig — Complete volatile emission data by compound and time point for each fungal species are given in S3–S6 Tables. (n = 3 or 5). The data underlying this Figure can be found at https://doi.org/10.6084/m9.figshare.21692156.v1. (TIF) [file pbio.3001887.s003.tif]

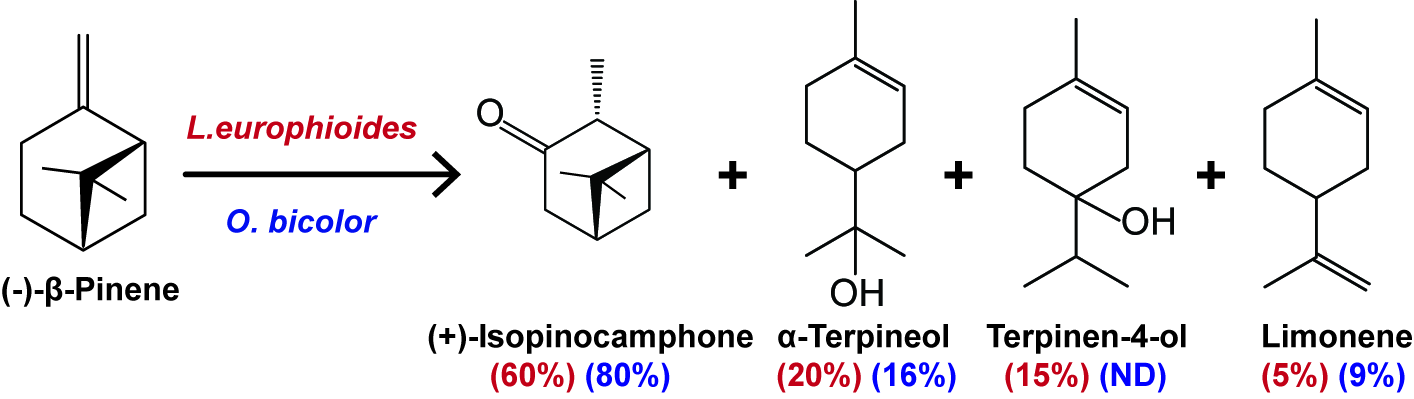

Supplement: S4 Fig — Isopinocamphone was the major biotransformation product (n = 4 or 5). E. polonica produced no detectable products. ND, not detected. The data underlying this Figure can be found at https://doi.org/10.6084/m9.figshare.21692156.v1. (TIF) [file pbio.3001887.s004.tif]

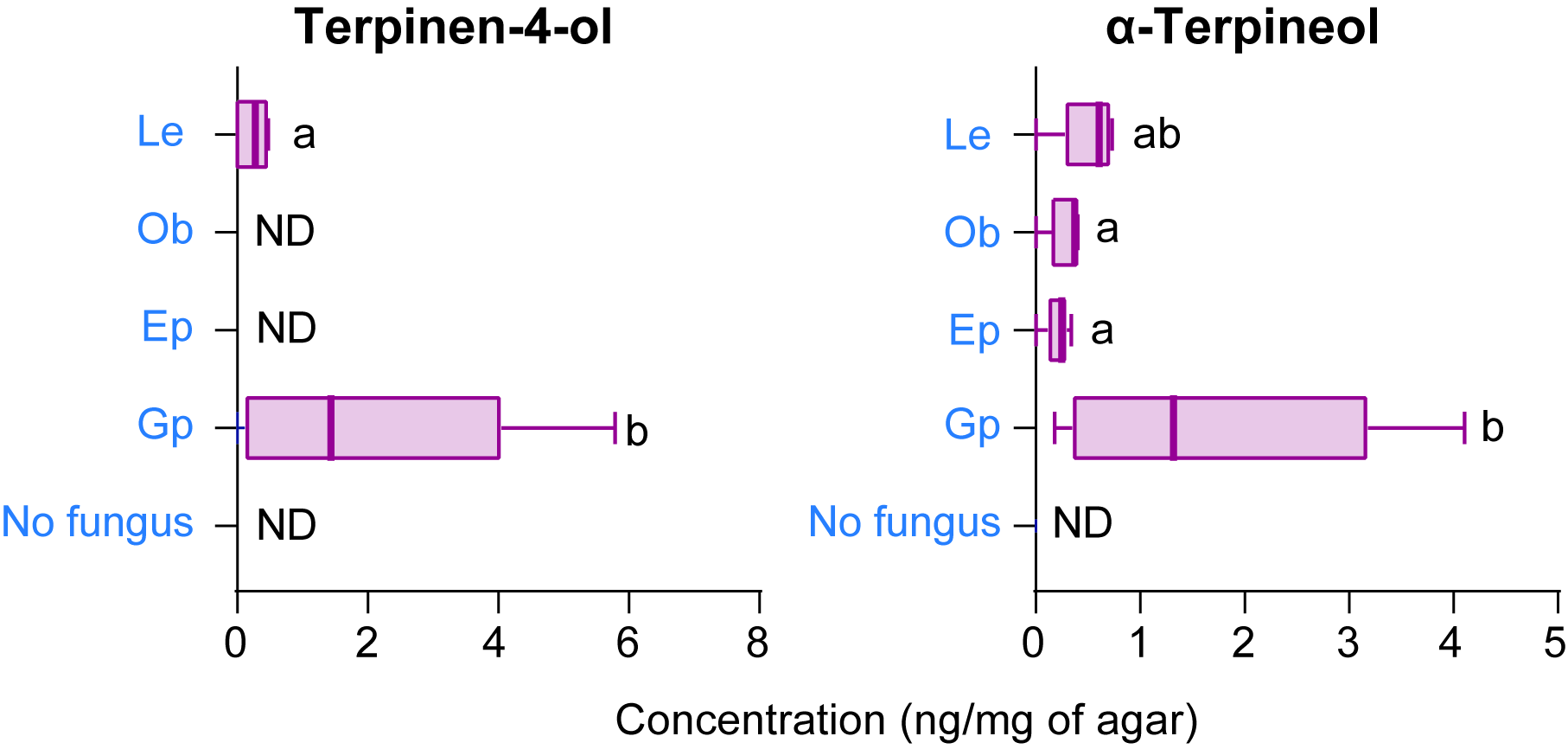

Supplement: S5 Fig — Amounts of metabolites were determined after hexane extraction of the agar. Error bars represent SEM (n = 5 or 11). ND, not detected. Different lowercase letters denote significant differences between treatments (ANOVA, Sidak’s test; P < 0.05). Fungal abbreviations: E. polonica (Ep), L. europhioides (Le), G. penicillata (Gp), O. bicolor (Ob). The data underlying this Figure can be found at https://doi.org/10.6084/m9.figshare.21692156.v1. (TIF) [file pbio.3001887.s005.tif]

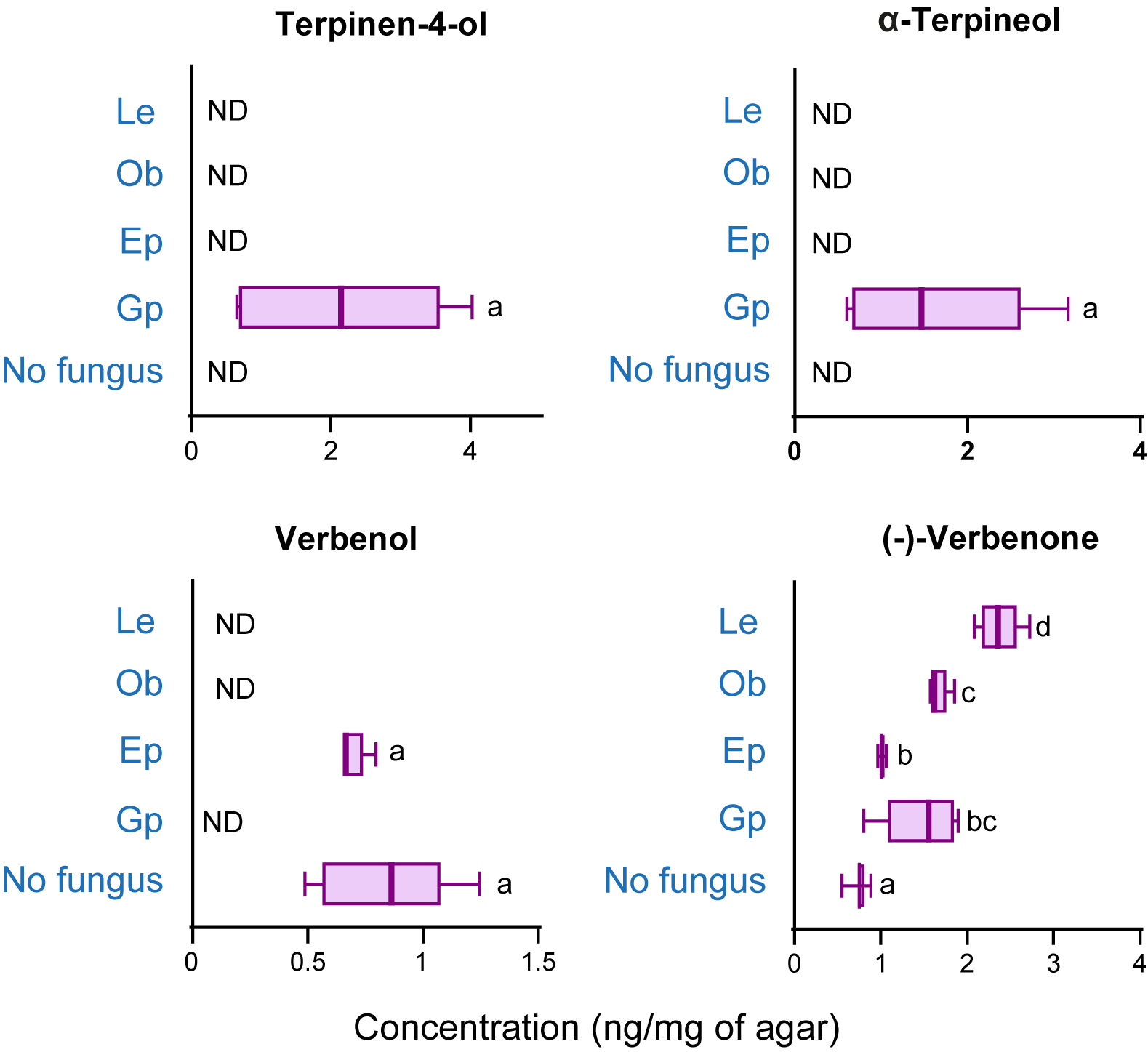

Supplement: S6 Fig — Amounts of metabolites were determined after hexane extraction of the agar. Error bars represent SEM (n = 5 to 12). ND, not detected. Different lowercase letters denote significant differences between treatments (ANOVA, Sidak’s test; P < 0.05). Fungal abbreviations: E. polonica (Ep), L. europhioides (Le), G. penicillata (Gp), O. bicolor (Ob). The data underlying this Figure can be found at https://doi.org/10.6084/m9.figshare.21692156.v1. (TIF) [file pbio.3001887.s006.tif]

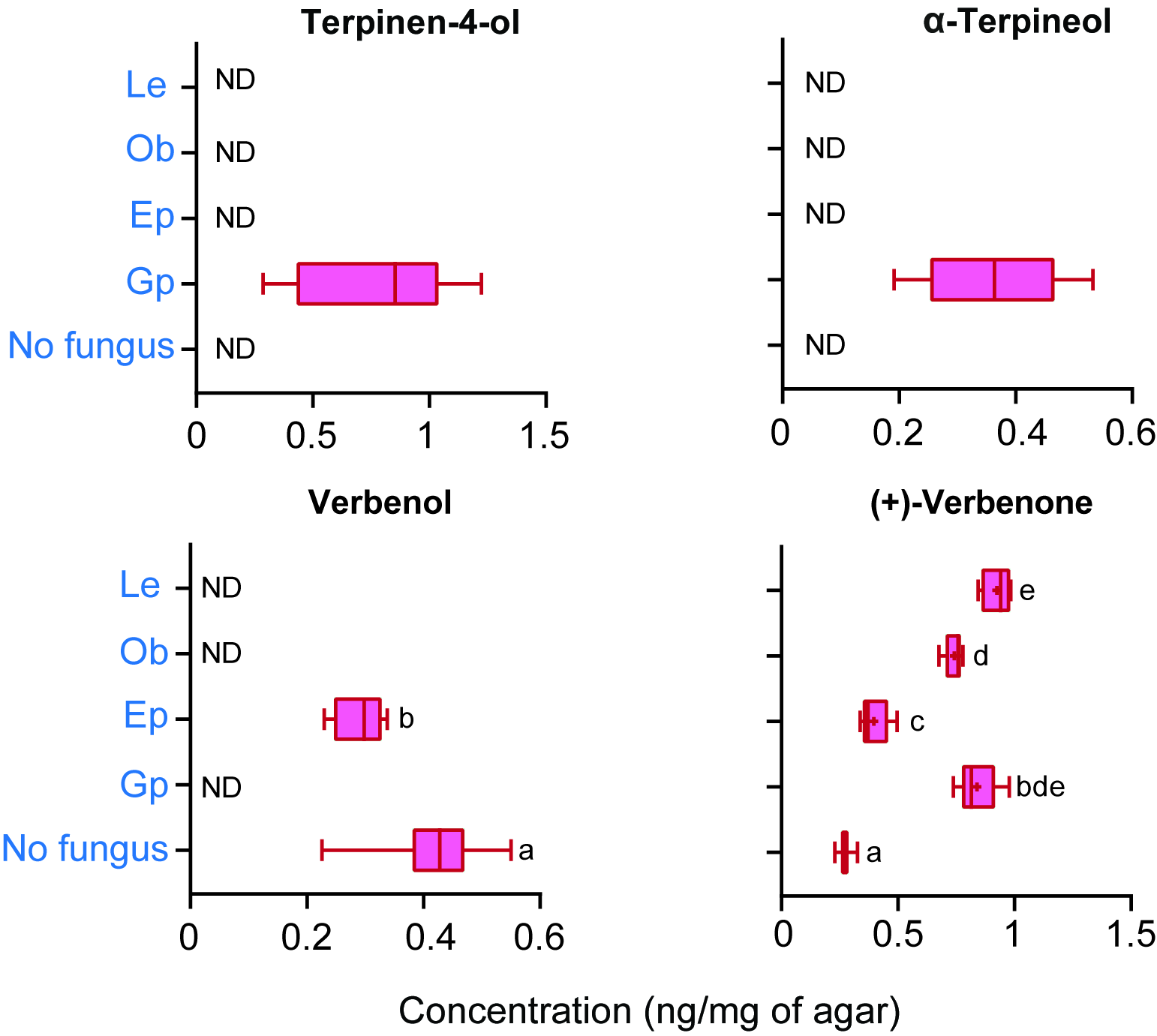

Supplement: S7 Fig — Amounts of metabolites were determined after hexane extraction of the agar. Error bars represent SEM (n = 5 or 13). ND, not detected. Different lowercase letters denote significant differences between treatments (ANOVA, Sidak’s test; P < 0.05). Fungal abbreviations: E. polonica (Ep), L. europhioides (Le), G. penicillata (Gp), O. bicolor (Ob). The data underlying this Figure can be found at https://doi.org/10.6084/m9.figshare.21692156.v1. (TIF) [file pbio.3001887.s007.tif]

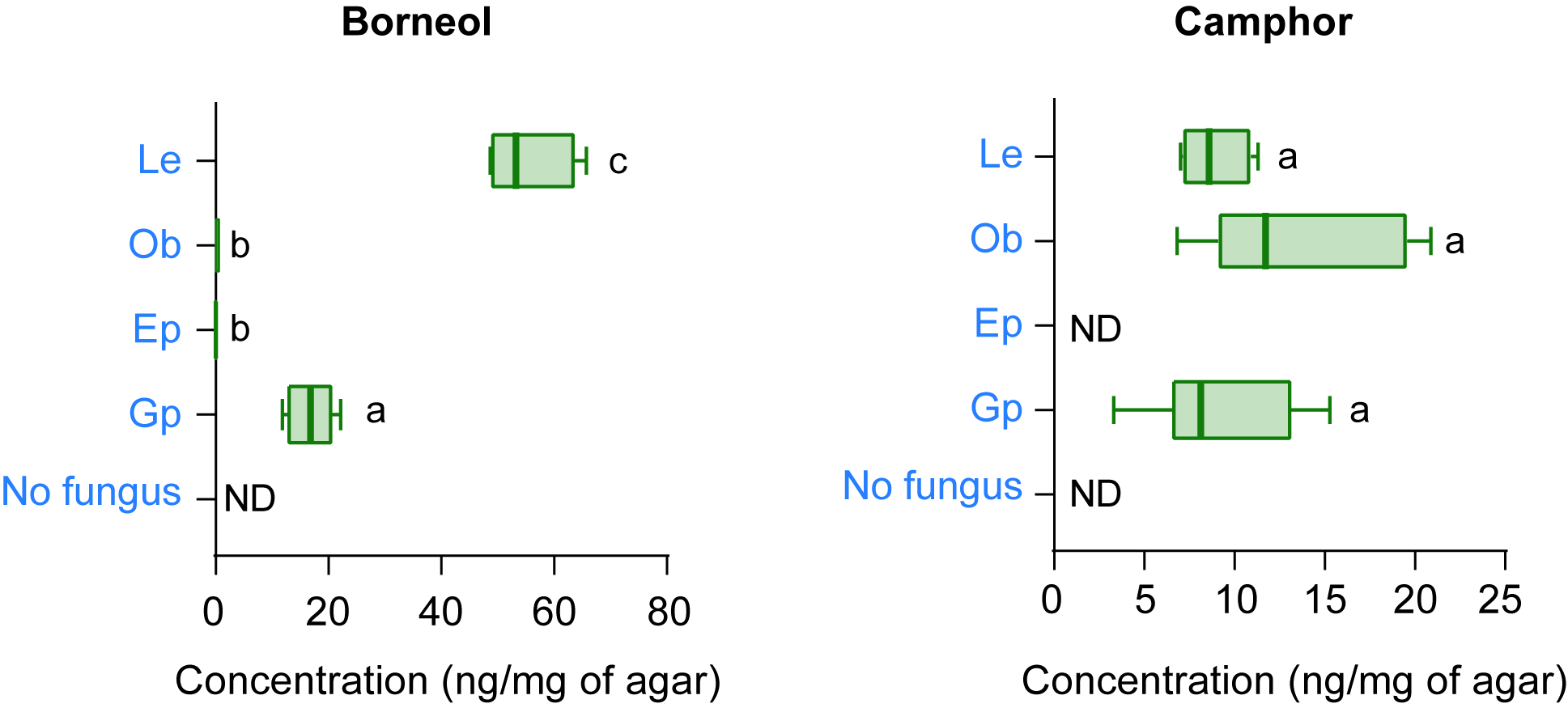

Supplement: S8 Fig — Amounts of metabolites were determined after hexane extraction of the agar. Error bars represent SEM (n = 5 or 13). ND, not detected. Different lowercase letters denote significant differences between treatments (ANOVA, Sidak’s test; P < 0.05). Fungal abbreviations: E. polonica (Ep), L. europhioides (Le), G. penicillata (Gp), O. bicolor (Ob). The data underlying this Figure can be found at https://doi.org/10.6084/m9.figshare.21692156.v1. (TIF) [file pbio.3001887.s008.tif]

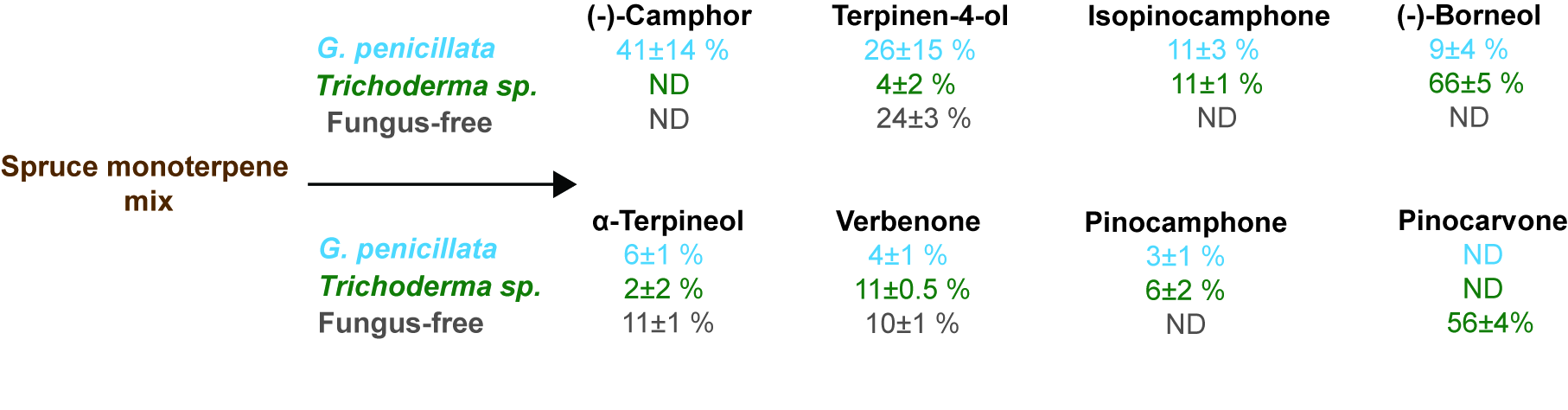

Supplement: S9 Fig — The data underlying this Figure can be found at https://doi.org/10.6084/m9.figshare.21692156.v1. (TIF) [file pbio.3001887.s009.tif]

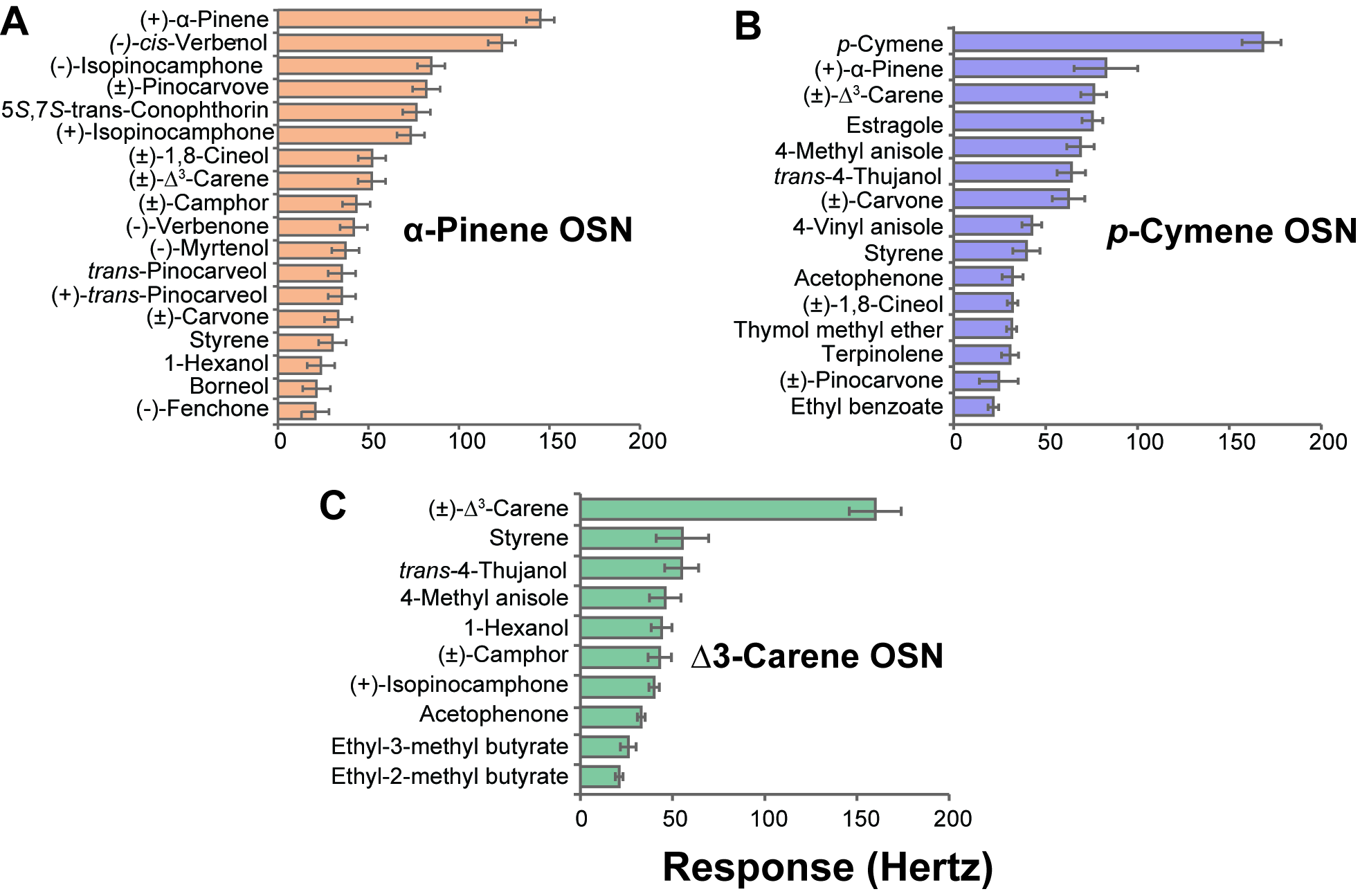

Supplement: S10 Fig — In addition to responses to the primary ligands, which are monoterpene hydrocarbons, these OSN classes show comparatively strong secondary responses to oxygenated monoterpenes produced by symbiotic fungi from host tree monoterpenes. Error bars represent SEM. The data underlying this Figure can be found at https://doi.org/10.6084/m9.figshare.21692156.v1. (TIF) [file pbio.3001887.s010.tif]

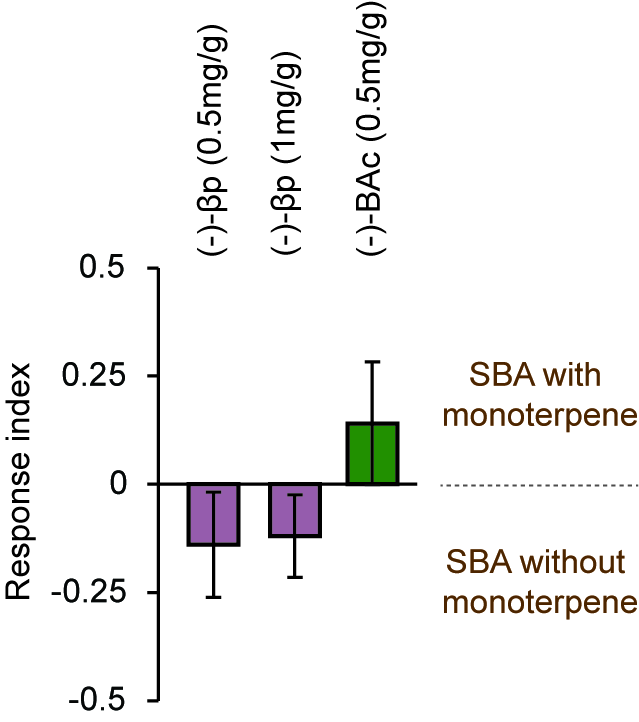

Supplement: S11 Fig — Error bars represent SEM (n = 25 for each trial). The data underlying this Figure can be found at https://doi.org/10.6084/m9.figshare.21692156.v1. (TIF) [file pbio.3001887.s011.tif]

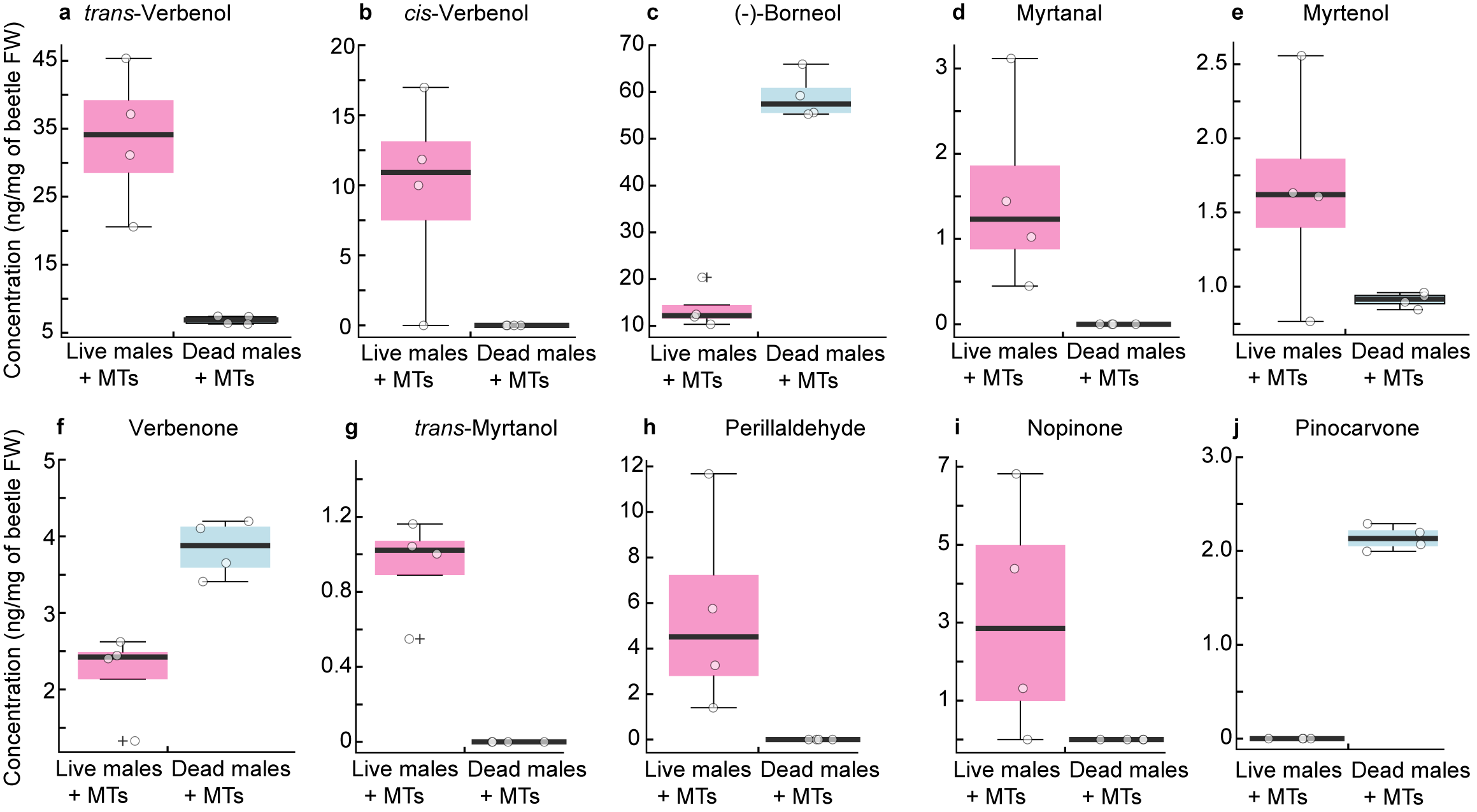

Supplement: S12 Fig — The method for chemical analysis of beetles is in the supplementary methods (S3 Method). The data underlying this Figure can be found at https://doi.org/10.6084/m9.figshare.21692156.v1. (TIF) [file pbio.3001887.s012.tif]

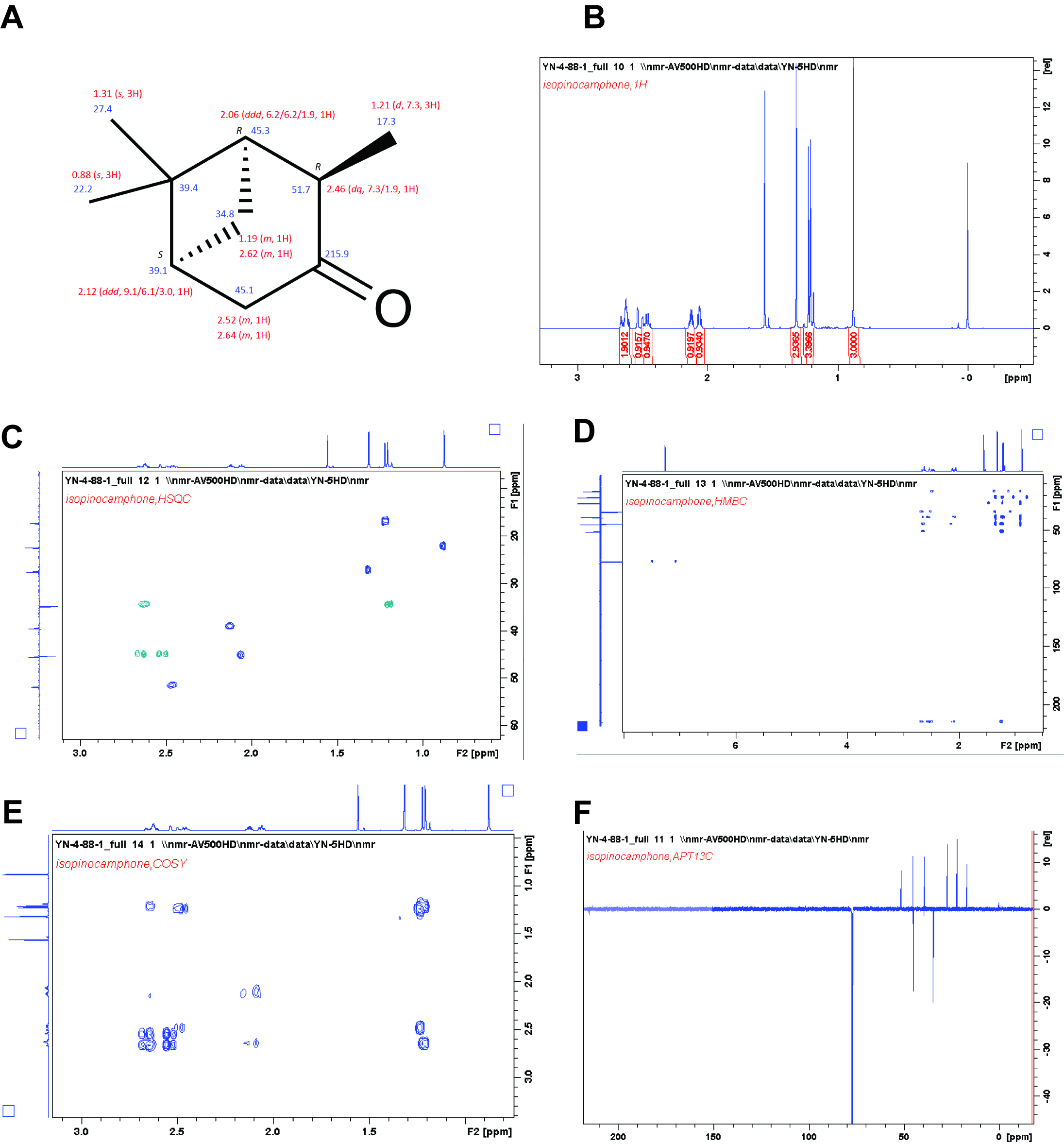

Supplement: S13 Fig — (A) 1H and 13C signal assignments in deuterated chloroform (CDCl3), (B) 1H NMR spectrum in CDCl3, (C) phase-sensitive heteronuclear single quantum coherence (HSQC) in CDCl3, (D) heteronuclear multiple bond correlation (HMBC) in CDCl3, (E) correlated spectroscopy (COSY) in CDCl3, and (F) 13C attached proton test (APT) in CDCl3. (TIF) [file pbio.3001887.s013.tif]

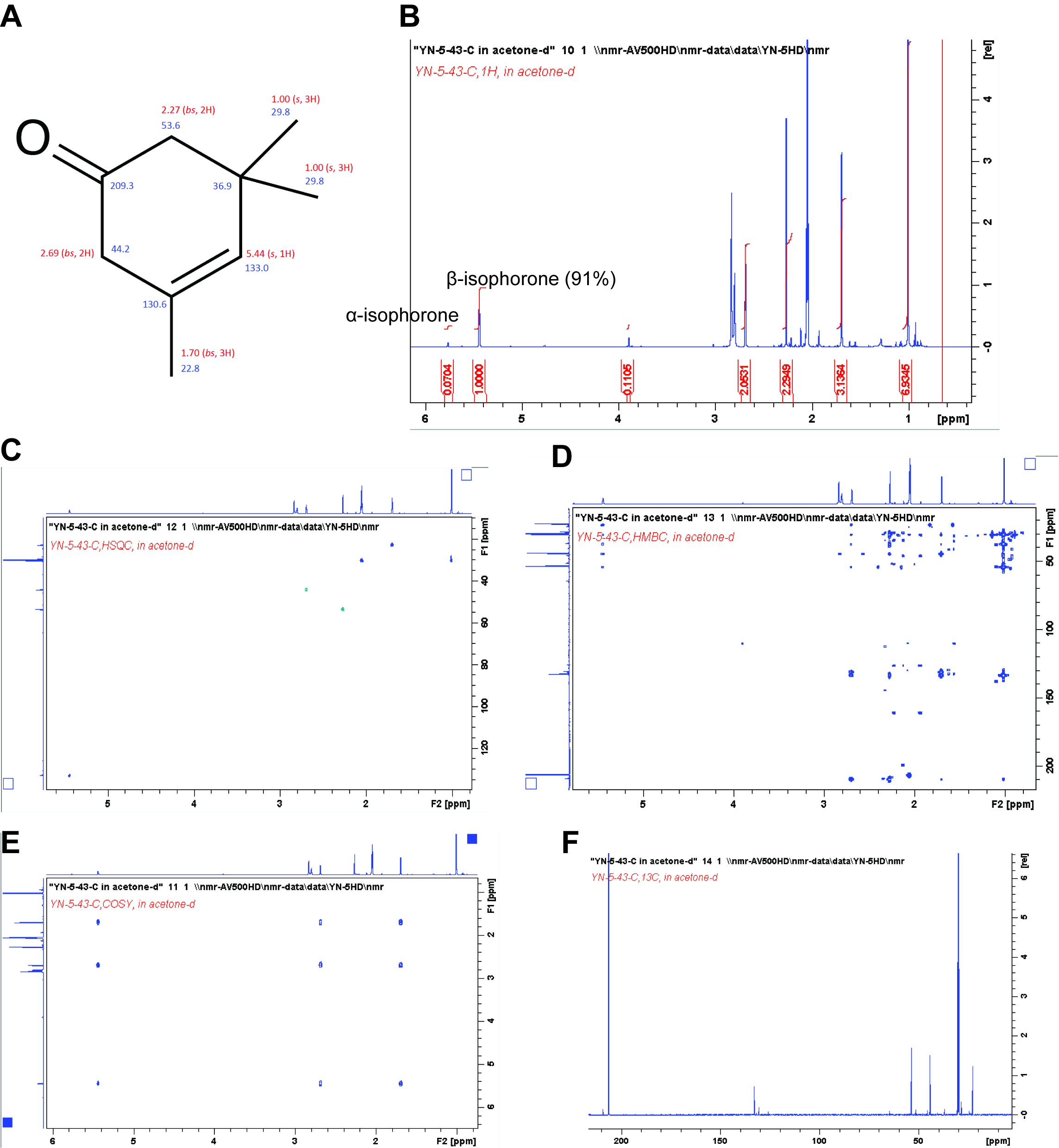

Supplement: S14 Fig — (A) 1H and 13C signal assignments in acetone-d6, (B) 1H NMR spectrum in acetone-d6, purity- 91%, (C) phase-sensitive heteronuclear single quantum coherence (HSQC) in acetone-d6, (D) heteronuclear multiple bond correlation (HMBC) in acetone-d6, (E) correlated spectroscopy (COSY) in acetone-d6, and (F) 13C spectrum in acetone-d6. (TIF) [file pbio.3001887.s014.tif]

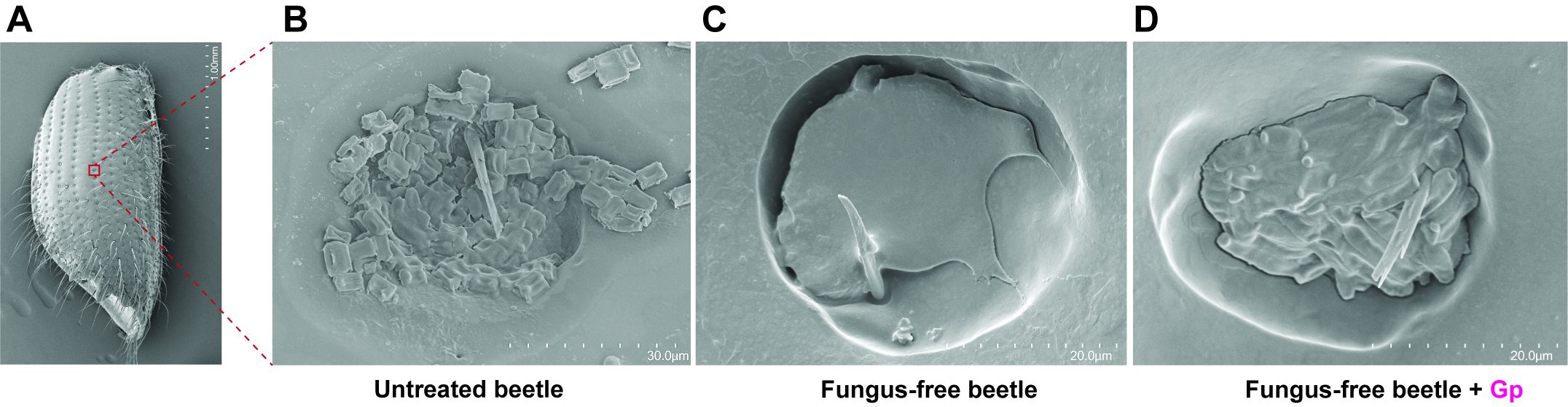

Supplement: S15 Fig — Scanning electron micrographs of (A) an elytron of an untreated bark beetle showing (B) spores of an ophiostomatoid fungus in the elytral pit, (C) an empty elytral pit of a fungus-free beetle, and (D) spore mass of G. penicillata in the elytral pit of a fungus-free beetle reinoculated with this fungal species. (TIF) [file pbio.3001887.s015.tif]
