# Supplementary material for: Conifer-killing bark beetles locate fungal symbionts by detecting volatile fungal metabolites of host tree resin monoterpenes
Source: PLoS Biol. 2023 Feb 21;21(2):e3001887. doi: 10.1371/journal.pbio.3001887 (PMC9943021; doi:10.1371/journal.pbio.3001887)
Supplement: S1 Table — (DOCX) [file pbio.3001887.s016.docx]

| ***Table S1***: Purity, source and biological origins of chemicals used in the experiments | | | |  |  |
| --- | --- | --- | --- | --- | --- |
| **Chemical** | **Purity (%)** | **Chemical source^1^** | **Biological origin** |  |  |
| Acetoin | >99 | Supelco | Fungi* |  |  |
| Acetophenone | 99 | Acros | Beetle/fungi^#^ |  |  |
| Amitinol | 96 | 1 | Beetle |  |  |
| Anisole | >99 | Sigma-Aldrich | Fungi^#^ |  |  |
| Benzaldehyde | >99 | Merck | Non-host/Fungi^*,#^ |  |  |
| Benzyl acetate | >99 | Aldrich | Fungi^*^ |  |  |
| Benzyl alcohol | >99 | Aldrich | Non-host/Fungi^*, #^ |  |  |
| Borneol | 94 | 2 | Fungi^#^ |  |  |
| (±)-2,3-Butanediol | >99 | Sigma-Aldrich | Fungi^*^ |  |  |
| (±)-Camphor | >99 | Aldrich | Host/fungi^#^ |  |  |
| (±)-∆3-Carene | 93 | Aldrich | Host |  |  |
| (±)-Carvone | >99 | Fluka | Fungi^#^ |  |  |
| (*E*)-β-Caryophyllene | >99 | Sigma | Host/fungi^*^ |  |  |
| β-Caryophyllene oxide | >99 | Givaudan-Roure | Fungi^*^ |  |  |
| Chavicol | >99 | Givaudan-Roure | Fungi^#^ |  |  |
| (±)-1,8-Cineole | >99 | Aldrich | Host |  |  |
| Cinnamaldehyde | >99 | Aldrich | Fungi^#^ |  |  |
| Cinnamyl alcohol | >99 | Aldrich | Fungi^#^ |  |  |
| Citral (*E* & *Z* mix) | >93 | Fluka | Fungi^*^ |  |  |
| (-)-Citronellol | >99 | Aldrich | Fungi^*^ |  |  |
| (+)-Citronellol | >99 | Aldrich | Fungi^*^ |  |  |
| β-Citronellyl acetate | >83 | 2 | Fungi^*^ |  |  |
| 5*S*,7*S*-*trans*-Conophthorin | 92 | 1 | Non-host/fungi^*,#^ |  |  |
| Coumaran | >99 | Sigma-Aldrich | Fungi^#^ |  |  |
| *p-*Cymene | >99 | Acros | Host |  |  |
| 4-Decanolide | ≥96 | Aldrich | Fungi^*^ |  |  |
| (-)-2,3-Dihydrofarnesol | >96 | 2 | Fungi^*^ |  |  |
| 3,4-Dimethoxytoulene | >98 | Sigma-Aldrich | Host/Fungi^#^ |  |  |
| Estragole | >99 | Fluka | Host/Fungi^#^ |  |  |
| Ethyl acetate | >99 | Sigma | Fungi^*^ |  |  |
| Ethyl benzoate | >99 | Fluka | Fungi^*^ |  |  |
| Ethyl butanoate | >99 | Firmenich | Fungi^*^ |  |  |
| Ethyl cinnamate | >99 | Aldrich | Fungi^#^ |  |  |
| 4-Ethyl guaiacol | >98 | Sigma | Fungi^#^ |  |  |
| Ethyl isobutyrate | >99 | Sigma-Aldrich | Fungi^*^ |  |  |
| Ethyl propionate | >99 | Aldrich | Fungi^*^ |  |  |
| Ethyl-2-methylbutyrate | >99 | Sigma-Aldrich | Fungi^*^ |  |  |
| Ethyl-3-methylbutyare | >99 | Sigma-Aldrich | Fungi^*^ |  |  |
| *p-*Eugenol | >99 | Fluka | Fungi^#^ |  |  |
| (*E,E*)-α-Farnesene | 95 | Fluka | Host/Fungi^*^ |  |  |
| (*E,E*)-Farnesol | 95 | Fluka | Fungi^*^ |  |  |
| (-)-Fenchol | >99 | 2 | Fungi^#^ |  |  |
| (-)-Fenchone | >99 | Fluka | Fungi^#^ |  |  |
| Geranyl acetate | >90 | Fluka | Fungi^*^ |  |  |
| Geranylacetone | >97 | Fluka | Non-host/fungi^*^ |  |  |
| 1-Hexanol | >99 | Fluka | Non-host/fungi^*^ |  |  |
| (±)-Ipsdienol | 94 | Bedoukian | Beetle |  |  |
| (±)-Ipsenol | 97 | Bedoukian | Beetle |  |  |
| Isoamyl acetate | >98 | Aldrich | Fungi^*^ |  |  |
| Isobutyl acetate | >99 | Fluka | Fungi^*^ |  |  |
| Isoeugenol | >99 | Aldrich | Fungi^#^ |  |  |
| α-Isophorone | >99 | Acros | NA |  |  |
| β-Isophorone | >90 | 3 | Beetle |  |  |
| (-)-Isopinocamphone | 96 | 4 | Host/fungi^#^ |  |  |
| (+)-Isopinocamphone | 92 | 3,4 | Host/fungi^#^ |  |  |
| 4-Methyl anisole | >99 | Aldrich | Fungi^#^ |  |  |
| Methyl cinnamate | >99 | Sigma-Aldrich | Fungi^#^ |  |  |
| Methyl eugenol | >90 | Sigma | Host/Fungi^#^ |  |  |
| 2-Methyl-1-butanol | >99 | Aldrich | Fungi^*^ |  |  |
| 3-Methyl-1-butanol | 99 | Aldrich | Fungi^*^ |  |  |
| 2-Methyl-3-buten-2-ol | >99 | Acros | Beetle/fungi^*^ |  |  |
| Myrcene | 95 | Fluka | Host |  |  |
| (-)-Myrtenol | 99 | 2 | Beetle/fungi^#^ |  |  |
| Neryl acetate | ≥97 | Aldrich | Fungi^*^ |  |  |
| (±)-3-Octanol | >99 | Acros | Non-host/fungi^*^ |  |  |
| (±)-Octen-3-ol | 98 | Acros | Non-host/fungi^*^ |  |  |
| Phenol | >99 | Sigma-Aldrich | Fungi^#^ |  |  |
| 3-Phenyl propanol | >99 | Sigma | Fungi^*^ |  |  |
| 2-Phenylethanol | >95 | Merck | Beetle/Fungi^*^ |  |  |
| 2-Phenylethyl acetate | >99 | Aldrich | Fungi^*^ |  |  |
| N-Phenylformamide | >99 | Alfa Aesar | Fungi^*^ |  |  |
| (+)-α-Pinene | >99 | Janssens chimica | Host |  |  |
| (*E*)-Pinocarveol | 67 | 2 | Fungi^#^ |  |  |
| (+)-(*E*)-Pinocarveol | 99 | 1 | Fungi^#^ |  |  |
| (±)-Pinocarvone | ~51^$^ | Synergy Corp. | Beetle/fungi^#^ |  |  |
| Propyl propionate | >99 | Acros | Fungi^*^ |  |  |
| Salicylaldehyde | >99 | Aldrich | Fungi^#^ |  |  |
| Styrene | 99 | Fluka | Fungi^#^ |  |  |
| Terpinolene | 97 | Fluka | Host |  |  |
| (+)-*trans*-4-Thujanol | >99 | Acros | Host/ Fungi^#^ |  |  |
| (-)-Terpinen-4-ol | >99 | Acros | Fungi^#^ |  |  |
| (+)-Terpinen-4-ol | >99 | Acros | Fungi^#^ |  |  |
| (-)-α-Terpineol | >99 | Fluka | Host/ Fungi^#^ |  |  |
| (+)-α-Terpineol | >99 | Fluka | Host/ Fungi^#^ |  |  |
| Thymol methyl ether | >99 | Sigma-Aldrich | Host |  |  |
| Toluene | >99 | Aldrich | Fungi^#^ |  |  |
| Vanillin | >99 | Sigma | Fungi^#^ |  |  |
| (-)-*cis*-Verbenol | 95 | Borregaard | Beetle |  |  |
| (+)-*trans*-Verbenol | 92 | SCM | Beetle |  |  |
| (-)-*trans*-Verbenol | 97 | Sci Tech | Beetle |  |  |
| (-)-Verbenone | >99 | Fluka | Beetle/fungi^#^ |  |  |
| 4-Vinyl anisole | >99 | Sigma-Aldrich | Fungi^#^ |  |  |
| ^1^Code to numbers: (1) Gift of Wittko Francke, University of Hamburg, Hamburg, Germany, (2) gift of Gunnar Bergström, University of Göteborg, Göteborg, Sweden, (3) synthesized in this study (4) gift of Rikard Unelius, Linnaeus University, Kalmar, Sweden. | | | | | |
| ^#^Fungal metabolite of host tree volatile | |  |  |  |  |
| ^*^Fungal volatile produced *de novo* | |  |  |  |  |
| ^$^None of the other components were active | |  |  |  |  |
| NA= Not applicable |  |  |  |  |  |
