# Supplementary material for: Conifer-killing bark beetles locate fungal symbionts by detecting volatile fungal metabolites of host tree resin monoterpenes
Source: PLoS Biol. 2023 Feb 21;21(2):e3001887. doi: 10.1371/journal.pbio.3001887 (PMC9943021; doi:10.1371/journal.pbio.3001887)
Supplement: S2 Table — Analyses were conducted using GC-FID. Compounds were identified by GC–MS analyses run in parallel. Compounds with significant P values are highlighted in bold. The data underlying this Table can be found at https://doi.org/10.6084/m9.figshare.21692156.v1. (DOCX) [file pbio.3001887.s017.docx]

***Table S2*:** Emission of volatile organic compounds identified from the headspace collection of fresh spruce bark four days after inoculation with different fungi. Analyses were conducted using GC-FID. Compounds were identified by GC-MS analyses run in parallel. Compounds with significant *P* values are highlighted in **bold.** The data underlying this Table can be found at https://doi.org/10.6084/m9.figshare.21692156.v1

|  | **RT^#^** | ***F^$^*** | ***P^$^*** | **Emission rate at 4 days post-inoculation (pg mg dry weight of bark^-1^ h^-1^)** | | | | | |
| --- | --- | --- | --- | --- | --- | --- | --- | --- | --- |
|  |  |  |  | **Uninfected** | ***E. polonica*** | ***G. penicillata*** | ***L. europhioides*** | ***O. bicolor*** | ***O. piceae*** |
| ***Monoterpenes*** |  |  |  |  |  |  |  |  |  |
| **Santene** | 5.70 | 7.97 | **<0.001** | 2.91±0.32^bc^ | 2.59±0.14^c^ | 2.75±0.22^bc^ | 4.66±0.51^ab^ | 5.41±0.7^a^ | 5.81±0.92^a^ |
| Tricyclene | 6.53 | 2.46 | 0.073 | 7.22±1.49 | 8.92±1.64 | 8.89±0.54 | 20.08±8.49 | 15.1±2.26 | 19.11±2.9 |
| **α-Thujene** | 6.68 | 4.25 | **0.01** | 4.8±0.86^b^ | 6.4±1.04^ab^ | 8.42±1.06^ab^ | 17.1±6.1^ab^ | 12.32±1.52^ab^ | 14.16±2^a^ |
| **α-Pinene** | 6.81 | 3.94 | **0.014** | 1308±236^b^ | 1942±424^ab^ | 2036±211^ab^ | 3733±1029^ab^ | 3471±531^ab^ | 4059±634^a^ |
| Camphene | 7.15 | 2.13 | 0.109 | 60.04±15.24 | 73.63±16.8 | 62.09±6.11 | 131±51.59 | 123±19.32 | 160±26.84 |
| ***o*-Cymene** | 7.70 | 3.3 | **0.027** | 2.87±1.53^a^ | 2.55±0.69^b^ | 7.76±1.61^ab^ | 5.61±1.42^ab^ | 5.08±0.8^ab^ | 7.55±1.37^ab^ |
| Sabinene | 7.76 | 2.26 | 0.134 | 20.63±5.01 | 33.89±5.82 | 24.28±3.88 | 13.02±7.63 | ND | ND |
| **β-Pinene** | 7.83 | 4.36 | **0.009** | 1871±313^b^ | 2885±566^ab^ | 3180±586^ab^ | 4696±681^ab^ | 5427±706^a^ | 5711±855^a^ |
| **Myrcene** | 8.20 | 7.57 | **<0.001** | 45.1±4.6^c^ | 70.94±12.72^bc^ | 99.5±30.6^abc^ | 189±27.84^a^ | 181±28^a^ | 150±17.58^ab^ |
| **α-Phellandrene** | 8.50 | 14.74 | **<0.001** | 4.36±0.46^c^ | 4.78±0.25^c^ | 7±0.75^bc^ | 12.53±1.58^a^ | 10.15±0.83^ab^ | 7.92±0.59^ab^ |
| Δ3-Carene | 8.64 | 1.89 | 0.146 | 83.99±20.9 | 116±24.87 | 182±23.94 | 211±48.86 | 114±11.09 | 155±15.23 |
| **α-Terpinene** | 8.80 | 2.97 | **0.042** | 3.24±0.21^b^ | 3.74±0.63^ab^ | 3.66±1.51^ab^ | 10.84±3.25^a^ | 4.84±0.54^ab^ | 6.09±0.96^ab^ |
| ***p*-Cymene** | 9.00 | 4.45 | **0.008** | 32.3±7.18^c^ | 47.09±9.09^bc^ | 66.61±13.95^abc^ | 73.87±9.06a^bc^ | 91.71±9.27^ab^ | 111±18.52^a^ |
| **β-Phellandrene** | 9.10 | 3.59 | **0.02** | 501±77.5^b^ | 773±161^ab^ | 726±159^ab^ | 1390±255^ab^ | 1395±196^a^ | 1161±152^ab^ |
| **γ-Terpinene** | 9.84 | 3.08 | **0.035** | 3.76±0.39^b^ | 3.93±1.23^ab^ | 4.04±0.98^ab^ | 8.95±1.41^a^ | 6.03±0.82^ab^ | 6.17±0.83^ab^ |
| ***Spiroketals/ others*** |  |  |  |  |  |  |  |  |  |
| ***endo*-1,3-Dimethyl-2,9-dioxabicyclo[3.3.1]nonane** | 9.36 | 8.16 | **<0.001** | ND | 1.85±1.17^c^ | 145±41.82^a^ | 82.7±10.5^abc^ | 126±16.49^ab^ | 34.67±13.58^bc^ |
| ***exo*-1,3-Dimethyl-2,9-dioxabicyclo[3.3.1]nonane** | 10.77 | 12.22 | **<0.001** | ND | 4.11±1.15^c^ | 144±32.23^ab^ | 203±28.94^a^ | 241±33.79^a^ | 72.88±20.94^bc^ |
| Nonanal | 10.95 | 0.55 | 0.738 | 8.99±1.07 | 7.12±0.62 | 8.19±0.47 | 9.33±0.75 | 8.95±1.23 | 9.07±1.31 |
| ***Oxygenated monoterpenes*** |  |  |  |  |  |  |  |  |  |
| ***trans*-4-Thujanol** | 10.06 | 4.93 | **0.005** | 1.85±0.35^b^ | 2.43±0.49^b^ | 7.69±2.85^ab^ | 11.61±1.43^a^ | 9.23±1.48^ab^ | 9.62±1.55^ab^ |
| **Linalool oxide** | 10.19 | 8.05 | **<0.001** | 2.66±0.36^c^ | 3.9±0.62^bc^ | 10.53±1.41^abc^ | 20.19±4.05^a^ | 22.98±3.45^a^ | 16.58±3.39^ab^ |
| **Terpinolene/Fenchone/Cymenene^1^** | 10.57 | 7.97 | **<0.001** | 12.88±2.68^d^ | 17.49±2.31^cd^ | 24.6±3.34^bcd^ | 54.18±10.15^a^ | 47.3±5.36^ab^ | 40.2±4.69^abc^ |
| **Fenchol** | 11.17 | 13.78 | **<0.001** | 4.32±1.52^cd^ | 0.58±0.38^d^ | 5.77±1.04^bcd^ | 9.86±1.42^bc^ | 14.38±1.85^ab^ | 20.73±3.02^a^ |
| **β-Thujone** | 11.26 | 8.81 | **<0.001** | 0.41±0.35^c^ | 1.72±0.22^bc^ | 2.79±0.23^bc^ | 2.45±0.83^bc^ | 7.21±1.34^a^ | 4.45±0.47^ab^ |
| Unknown monoterpene #1 | 11.48 | 1.55 | 0.224 | 0.64±0.33 | 1.25±0.38 | 83.05±18.84 | 19.92±2.83 | 4.5±0.61 | 37.48±15.23 |
| ***trans*-Pinocarveol** | 11.78 | 10.75 | **<0.001** | 22.38±5.09^d^ | 48.71±7.24^cd^ | 83.4±9.73^bcd^ | 96.03±15.7^abc^ | 182.7±24.65^a^ | 144.18±27.28^ab^ |
| **Camphor** | 11.92 | 77.48 | **<0.001** | 26.32±3.4^c^ | 96.46±18.78^c^ | 1280±140^b^ | 1565±249^ab^ | 2537±256^a^ | 859±98.63^b^ |
| **Isoborneol** | 12.22 | 8.94 | **0.002** | ND | ND | 9.94±1.06^b^ | 11±1.37^b^ | 13.7±1.51^b^ | 30.24±6.16^a^ |
| **Pinocamphone** | 12.30 | 5.85 | **0.002** | 93.16±16.52^b^ | 147±24.64^ab^ | 165±7.67^ab^ | 260±53.62^a^ | 281±28^a^ | 287±35.54^a^ |
| Pinocarvone | 12.36 | 0.62 | 0.46 | ND | ND | ND | ND | 55.9±5.39 | 67.82±11.1 |
| ***endo*-Borneol** | 12.43 | 36.28 | **<0.001** | 13.15±4.94^d^ | 80.66±12.15^cd^ | 225±27.93^bc^ | 375±50.6^b^ | 175±30.35^bcd^ | 166±5396.75^a^ |
| **Isopinocamphone** | 12.63 | 14.57 | **<0.001** | 41.9±9.38^d^ | 88.28±22.45^cd^ | 137±7.53^cd^ | 453±100.9^a^ | 197±21.96^bcd^ | 368±52.31^ab^ |
| **Terpinen-4-ol** | 12.70 | 10.72 | **<0.001** | ND | 7.09±1.87^c^ | 94.91±55.52^ab^ | 22.17±4.43^bc^ | 101±17.52^ab^ | 136±39.66^a^ |
| *p*-Cymene-8-ol | 12.88 | 2.08 | 0.115 | 3.32±1.18 | 7.56±1.46 | 23.89±7.32 | 43.53±7.31 | 55.52±11.76 | 31.03±10.76 |
| α-Terpineol | 12.95 | 1.65 | 0.23 | ND | ND | 5.2±1.09 | 16.2±3.45 | 13.14±4.4 | 12.85±2.78 |
| **Verbenone** | 13.18 | 6.37 | **<0.001** | 41.73±6.51^b^ | 62.7±4.64^b^ | 95.64±8.62^b^ | 126±18.92^ab^ | 216±34.26^a^ | 127±36.35^ab^ |
| **Thymol methyl ether** | 14.22 | 7.73 | **<0.001** | 3.12±0.63^c^ | 8.22±1.19^bc^ | 20.85±2.69^abc^ | 36.23±4.1^ab^ | 47.37±7.55^a^ | 44.02±12.19^a^ |
| ***p*-Menth-2-en-7-ol** | 14.55 | 5.78 | **0.005** | ND | 12.82±5.13^c^ | 32.37±5.65^abc^ | 52.91±5.65^ab^ | 59.88±12.28^a^ | 15.15±8.26^bc^ |
| **Myrtanol** | 14.58 | 15.66 | **<0.001** | 6.43±0.63^c^ | 10.1±1^c^ | 17.05±2.62^bc^ | 38.95±5.71^ab^ | 61.37±12.71^a^ | 72.98±19.9^a^ |
| **Bornyl acetate** | 15.20 | 15.11 | **<0.001** | 14.09±2.77^bc^ | 53.18±5.36^a^ | 44.41±6.59^a^ | 23.09±1.55^ab^ | 7.6±0.41^c^ | 51.85±11.15^a^ |
| Thymol | 15.30 | 0.99 | 0.453 | 0.54±0.27 | 5.87±1.24 | 13.97±3.84 | 45.19±6.78 | 10.41±2.14 | 13.99±4.86 |
| ***Sesquiterpenes*** |  |  |  |  |  |  |  |  |  |
| α-Longipinene | 16.63 | 2.15 | 0.106 | 10.62±1.17 | 14.7±1.98 | 13.65±3.15 | 28.01±7.48 | 21.66±2.12 | 17.53±3.11 |
| Unknown SqT #1 | 16.69 |  |  | ND | ND | ND | ND | ND | 11.98±2.39 |
| Cyclosativene | 17.08 | 2.21 | 0.099 | 6.05±0.93 | 8.48±0.82 | 7.27±1.41 | 12.64±2.86 | 10.39±0.89 | 6.42±0.97 |
| **α-Cubebene** | 17.17 | 2.99 | **0.039** | 2.65±0.36^a^ | 3.38±0.33^a^ | 2.73±0.6^a^ | 8.12±2.57^a^ | 6.4±0.53^a^ | 3.36±0.66^a^ |
| 6-Protoilludene | 17.26 |  |  | ND | ND | ND | ND | ND | 188.97±39.53 |
| Gurjunene | 17.48 | 2.23 | 0.096 | 5.48±1.25 | 6.27±0.8 | 4.09±1.37 | 10.09±1.28 | 7.64±1.25 | 8.72±1.57 |
| Isolongifolene | 17.81 | 2.28 | 0.09 | 39.35±5.05 | 59.72±8.29 | 49.94±9.95 | 86.56±20.9 | 71.7±6.33 | 40.86±7.04 |
| α-Cedrene | 17.95 | 2.75 | **0.051** | 3.57±0.59^ab^ | 5.1±0.65^ab^ | 4.68±0.71^ab^ | 8.35±2.16^a^ | 5.41±0.38^ab^ | 3.26±0.57^b^ |
| (*E*)-β-Caryophyllene | 18.10 | 1.87 | 0.151 | 5.56±0.9 | 7.83±0.93 | 9.22±1.19 | 16.3±4.94 | 10.42±0.83 | 11.29±2.17 |
| (*E*)-β-Caryophyllene (fungus) | 18.48 | 1.09 | 0.336 | ND | ND | 6.08±1.64 | 8.62±1.33 | ND | ND |
| Δ-Cadinene | 18.71 | 1.22 | 0.341 | 1.95±0.49 | 3.28±0.32 | 2.14±0.34 | 3.54±0.59 | 2.91±0.13 | 2.63±0.8 |
| **Humulene/(*E*)-β-Farnesene^1^** | 18.81 | 3.94 | **0.014** | 4.06±0.67^b^ | 5.38±0.46^ab^ | 7.99±0.97^ab^ | 9.49±1.31^a^ | 9.87±0.57^a^ | 6.36±1.58^ab^ |
| Amorphene | 19.25 | 2.34 | 0.084 | 9.48±1.23 | 12.4±1.42 | 10.5±1.35 | 25.18±9.14 | 21.88±1.79 | 11.47±2.73 |
| Cubenene | 19.35 | 1.68 | 0.19 | 22.25±1.77 | 34.05±3.73 | 33.1±7.12 | 75.84±30.03 | 49.04±5.76 | 31.45±8.37 |
| α-Muurolene | 19.72 | 1.72 | 0.18 | 10.17±1.69 | 15.65±2.01 | 11.35±1.17 | 22.48±7.08 | 17.14±1.27 | 9.74±2.5 |
| **Unknown SqT #2** | 20.09 | 3.33 | **0.027** | 1.8±0.27^ab^ | 1.53±0.11^ab^ | 1.54±0.04^ab^ | 2.3±0.15^a^ | 2.2±0.21^ab^ | 1.14±0.34^b^ |
| Δ-Cadinene | 20.17 | 2.7 | 0.054 | 30.38±2.83 | 53.94±7.58 | 42.88±4.92 | 88.63±23.85 | 80.15±6.76 | 50.83±14.2 |
| **Germacrene B** | 20.84 | 3.07 | **0.035** | 2.91±0.58^a^ | 5.81±0.82^a^ | 5.52±0.85^a^ | 5.24±0.69^a^ | 3.19±0.43^a^ | 2.67±0.81^a^ |
| Caryophyllene oxide | 21.94 | 4 | 0.093 | ND | ND | 24.06±6.27 | 9.16±1.76 | ND | ND |
|  |  |  |  |  |  |  |  |  |  |

^#^- Estimated retention time using the method developed for GC-FID described in the materials and methods section of this manuscript

***^$^*** Significant differences between species are denoted by small letters (ANOVA, followed by Tukey’s test, *P<0.05)*

^1^-Compounds co-eluted from the GC column.

Unknown monoterpene #1: M^+^ = *m/z* 148

Unknown SqT (sesquiterpene) #1: M^+^ = *m/z* 204.2

Unknown SqT (sesquiterpene) #2: M^+^ = *m/z* 20
