# Supplementary material for: Conifer-killing bark beetles locate fungal symbionts by detecting volatile fungal metabolites of host tree resin monoterpenes
Source: PLoS Biol. 2023 Feb 21;21(2):e3001887. doi: 10.1371/journal.pbio.3001887 (PMC9943021; doi:10.1371/journal.pbio.3001887)
Supplement: S3 Table — Data from the control uninfected treatment are presented here. Data for fungal treatments are given in S3–S6 Tables. Volatiles were collected on polydimethylsiloxane tubes for 2 h and were subjected to GC–MS analysis (see Materials and methods section for details). ND, not detected, NA, not analyzed, TR, trace amounts (<500 TIC counts). The data underlying this Table can be found at https://doi.org/10.6084/m9.figshare.21692156.v1. (DOCX) [file pbio.3001887.s018.docx]

***Table S3***. Relative amounts (mean ± SE, n=3) of volatiles from uninoculated bark detected after various time periods (4, 8, 12 and 18 days) from the beginning of an experiment with fungal inoculation. Data from the control uninfected treatment are presented here. Data for fungal treatments are given in Tables S3-S6. Volatiles were collected on polydimethylsiloxane tubes for 2 hours and were subjected to GC-MS analysis (see materials and methods section for details). ND=not detected, NA=not analyzed, TR= trace amounts (<500 TIC counts). The data underlying this Table can be found at https://doi.org/10.6084/m9.figshare.21692156.v1

| ***Compounds*** | **RT^#^** | ***F^$^*** | ***P^$^*** | **Uninoculated bark peak area (*10^4^ TIC counts)** | | | |
| --- | --- | --- | --- | --- | --- | --- | --- |
|  |  |  |  | **4d** | **8d** | **12d** | **18d** |
| ***Aliphatics*** |  |  |  |  |  |  |  |
| 2-Butanone | 1.85 | - | - | ND | ND | ND | 2.86±0 |
| 2-Methyl-3-buten-2-ol | 1.93 | - | - | ND | ND | ND | 0.08±0 |
| Ethyl acetate | 1.95 | - | - | ND | ND | ND | ND |
| Isobutanol | 2.40 | - | - | ND | ND | ND | ND |
| Isopropyl acetate | 2.33 | - | - | ND | ND | ND | ND |
| **Acetoin** | 2.85 | 19.32 | **0.005** | 6.57±0.11(a) | 6.49±1.05(a) | 4.27±0.8(ab) | 0.09±0.05(b) |
| Ethyl propanoate | 2.88 | - | - | ND | 1.6±0.15 | ND | ND |
| 3-Methyl-1-butanol | 3.24 | - | - | ND | ND | 0.62±0.11 | 0.72 |
| Ethyl isobutyrate | 3.69 | - | - | ND | 0.07±0 | ND | ND |
| Isobutyl acetate | 3.99 | 1.16 | 0.341 | ND | 0.08±0.01 | 0.1±0.01 | ND |
| 2,3-Butanediol | 4.17 | - | - | ND | ND | ND | ND |
| Ethyl butanoate | 4.55 | - | - | ND | ND | 0.05±0 | ND |
| Ethyl but-2-enoate | 5.60 | - | - | ND | ND | ND | ND |
| Ethyl 2-methylbutyrate | 5.75 | - | - | ND | ND | ND | ND |
| 1-Hexanol | 6.25 | - | - | ND | ND | 0.14±0 | 0.07±0 |
| 3-Methyl-1-butyl acetate | 6.46 | - | - | ND | 10.78±0.54 | 0.04±0 | ND |
| Isopentyl-2-methylbutanoate | 12.47 | - | - | ND | ND | ND | ND |
| Isoamyl valerate | 12.60 | - | - | ND | ND | ND | TR |
| **Sum** |  | 10.45 | **0.006** | 4.37±2.19(b) | 16.84±2.26(a) | 3.45±1.91(b) | 1.98±1.93(b) |
| ***Aromatics*** |  |  |  |  |  |  |  |
| 2-Phenylethyl alcohol | 12.79 | - | - | ND | ND | ND | ND |
| 2-Phenylethyl acetate | 16.39 | - | - | ND | 0.07±0 | ND | ND |
| Citronellyl acetate | 18.58 | - | - | ND | ND | ND | ND |
| Sum |  | 9.71 | **0.02** | 0 | 0.06±0.01 | 0 | 0 |
| ***Spiroketals*** |  |  |  |  |  |  |  |
| *endo-*1,3-dimethyl-2,9-dioxabicyclo[3.3.1]nonane | 10.81 | - | - | ND | ND | ND | ND |
| *trans*-Conophthorin | 11.29 | - | - | ND | ND | ND | 0.03±0 |
| Brevicomin | 11.64 | - | - | ND | ND | ND | ND |
| *exo-*1,3-dimethyl-2,9-dioxabicyclo[3.3.1]nonane | 12.37 | - | - | ND | ND | 0.06±0.01 | 0.14 |
| Sum |  |  |  | 0 | 0 | 0.03±0.02 | 0.08±0 |
| ***Monoterpenes*** |  |  |  |  |  |  |  |
| Santene | 6.61 | 0.94 | 0.357 | 0.34±0.11 | 0.09±0.03 | 0.09±0.03 | 0.14±0 |
| **Tricyclene** | 7.67 | 6.94 | **0.027** | 2.57±0.35(a) | 0.58±0.14(ab) | 0.23±0.07(ab) | 0.41±0.11(b) |
| α-Thujene | 7.76 | 3.36 | 0.1 | 0.73±0.09 | 0.12±0.03 | 0.04±0.01 | 0.19±0.06 |
| **α-Pinene** | 7.94 | 8.77 | **0.016** | 465±89.61(a) | 122±29.58(ab) | 57.75±14.54(ab) | 72.2±14.92(b) |
| **Camphene** | 8.34 | 9.33 | **0.014** | 7.67±1.38(a) | 2.4±0.5(ab) | 1.06±0.23(ab) | 1.44±0.1(b) |
| **Verbenene** | 8.51 | 12.18 | **0.007** | 0.35±0.08(a) | 0.08±0.02(ab) | 0.05±0.01(b) | 0.04±0(b) |
| Sabinene | 9.50 | - | - | ND | ND | ND | ND |
| **β-Pinene** | 9.13 | 17.75 | **0.002** | 866±148(a) | 188±51.19(ab) | 76.37±25.05(b) | 68.05±10.8(b) |
| **β-Myrcene** | 9.54 | 6.44 | **0.035** | 7.48±1.31(a) | 1.62±0.63(a) | 1.05±0.36(a) | 1.08±0.04(a) |
| **α-Phellandrene** | 9.88 | 10.6 | **0.01** | 0.61±0.1(a) | 0.27±0.09(ab) | 0.03±0.03(ab) | 0.18±0(b) |
| α-Terpinene | 10.21 | - | - | 0.1±0 | ND | ND | ND |
| *p*-Cymene | 10.43 | 4.88 | 0.054 | 10.29±3.01 | 4.27±1.4 | 2.56±0.73 | 2.7±0.85 |
| **Limonene** | 10.51 | 7.9 | **0.02** | 26.08±5.15(a) | 6.91±1.85(ab) | 3.11±0.88(b) | 4.11±0.69(ab) |
| **β-Phellandrene** | 10.55 | 6.1 | **0.036** | 56.99±9.75(a) | 18.3±5.48(ab) | 7.48±2.11(ab) | 13.6±0.94(b) |
| γ-Terpinene | 11.37 | - | - | ND | ND | ND | ND |
| α-Terpinolene | 12.16 | - | - | ND | ND | ND | 0.09±0 |
| *p*-Cymenene | 12.19 | 0 | 0.983 | 0.1±0.02 | 0.06±0.02 | 0.1±0 | 0.09±0.05 |
| Sum |  | 11.56 | **0.004** | 1443±307(a) | 344±105(ab) | 149±46.9(b) | 164±38.2(b) |
| ***Oxygenated monoterpenes*** |  |  |  |  |  |  |  |
| **1,8-Cineole** | 10.61 | 10.71 | **0.011** | 6.27±1.55(a) | 2.8±0.94(ab) | 0.58±0.22(ab) | 0.02±0(b) |
| Linalool oxide | 11.73 | - | - | ND | ND | ND | TR |
| Fenchone | 12.15 | 0 | 0.961 | 0.54±0.28 | 0.68±0.27 | 0.39±0.19 | 0.59±0.15 |
| *trans*-4-Thujanol | 12.42 | - | - | ND | ND | ND | ND |
| *exo*-Fenchol | 12.82 | 0.03 | 0.875 | 0.09±0.03 | 0.06±0.02 | 0.05±0.02 | 0.09±0.05 |
| β-Thujone | 12.93 | - | - | 0.09±0.01 | TR | ND | ND |
| *p*-Isopropylcyclohexanol | 13.41 | - | - | ND | ND | ND | ND |
| *trans*-Pinocarveol | 13.48 | 1.36 | 0.274 | 0.27±0 | 0.08±0.01 | 0.27±0.03 | 0.18±0.05 |
| Camphor | 13.63 | 1.07 | 0.329 | 1.34±1.02 | 1.34±0.98 | 1.09±0.74 | 2.45±0.28 |
| Camphene hydrate | 13.73 | 0.49 | 0.71 | 0.22±0.06 | 0.2±0.06 | 0.16±0.05 | 0.08±0.01 |
| Pinocamphone | 14.43 | 0.46 | 0.513 | 2.71±0.87 | 1.43±0.7 | 0.93±0.4 | 0.92±0.22 |
| Pinocarvone | 14.10 | - | - | ND | ND | ND | ND |
| *endo*-Borneol | 14.18 | 2.29 | 0.164 | 0.48±0.16 | 1.2±0.72 | 2.85±1.26 | 2.2±0.53 |
| 3-Thujene-2-one | 14.34 | - | - | ND | ND | 0.02±0 | ND |
| Isopinocamphone | 14.40 | 0.18 | 0.681 | 1.3±0.68(a) | 0.91±0.54(a) | 1.27±0.62(a) | 1.36±0.02(a) |
| Terpinen-4-ol | 14.46 | 1.58 | 0.249 | 0.24±0.15(a) | 0.07±0.01(a) | 0.04±0(a) | 0.04±0.01(a) |
| ***p*-Cymene-8-ol** | 14.65 | 8.95 | **0.03** | 0.03±0(a) | 0.02±0(a) | 0.43±0.06(a) | 0.15±0.09(a) |
| α-Terpineol | 14.79 | 0.73 | 0.416 | 0.67±0.34 | 0.29±0.14 | 0.27±0.04 | 0.37±0.22 |
| Myrtenol | 14.94 | - | - | ND | ND | 0.4±0.2 | 0.32±0.2 |
| Verbenone | 15.28 | - | - | ND | ND | ND | ND |
| 2-Hydroxycineole | 15.58 | - | - | ND | ND | TR | TR |
| Thymol methyl ether | 15.85 | 0.66 | 0.436 | 1.03±0.21 | 0.67±0.23 | 0.54±0.22 | 0.76±0.17 |
| Myrtanol isomer1 | 16.73 | - | - | ND | ND | ND | ND |
| Myrtanol isomer2 | 16.30 | - | - | ND | ND | ND | ND |
| *p*-Menth-2-en-7-ol | 16.42 | - | - | ND | ND | ND | ND |
| Myrtanol isomer3 | 16.48 | - | - | ND | ND | ND | ND |
| Myrtenyl acetate isomer1 | 17.43 | - | - | ND | ND | 0.87±0 | ND |
| Myrtenyl acetate isomer2 | 18.13 | - | - | ND | ND | ND | ND |
| Sum |  |  |  | 14.95±5.07 | 9.41±4.06 | 8.92±4.23 | 9.58±0.44 |
| ***Sesquiterpenes*** |  |  |  |  |  |  |  |
| **α-Longipinene** | 18.63 | 12.26 | **0.007** | 0.2±0.03(a) | 0.09±0.02(ab) | 0.03±0(b) | 0.04±0.02(b) |
| **Longicyclene** | 19.10 | 10.45 | **0.012** | 0.26±0.04(a) | 0.07±0.01(a) | 0.03±0.01(a) | 0.06±0(a) |
| **Longifolene** | 19.88 | 6.48 | **0.031** | 2.07±0.32(a) | 1.07±0.22(a) | 0.42±0.11(a) | 0.74±0.46(a) |
| **(*E*)-β-Caryophyllene** | 20.17 | 20.58 | **0.001** | 8.59±0.44(a) | 4.01±0.47(ab) | 1.47±0.26(b) | 1.61±1.02(b) |
| (*E*)-β-Caryophyllene (fungus) | 20.56 | - | - | ND | ND | ND | ND |
| (*E*)-β-Farnesene | 20.84 | - | - | ND | ND | ND | ND |
| **Humulene** | 20.90 | 18.59 | **0.002** | 3.1±0.36(a) | 1.6±0.24(ab) | 0.67±0.15(b) | 0.57±0.32(b) |
| Caryophyllene oxide | 23.56 | - | - | ND | ND | ND | ND |
| Sum |  | 18.74 | **0.001** | 14.22±1.15(a) | 6.83±1.05(b) | 2.61±0.63(b) | 2.99±2.6(b) |

^#^- Estimated retention time from GC-MS

***^$^-***Significant differences between time points are denoted by small letters (ANOVA, followed by Tukey’s test, *P<0.05)*
