# Supplementary material for: Conifer-killing bark beetles locate fungal symbionts by detecting volatile fungal metabolites of host tree resin monoterpenes
Source: PLoS Biol. 2023 Feb 21;21(2):e3001887. doi: 10.1371/journal.pbio.3001887 (PMC9943021; doi:10.1371/journal.pbio.3001887)
Supplement: S4 Table — Volatiles were collected on polydimethylsiloxane tubes for 2 h and were subjected to GC–MS analysis (see Materials and methods section for details). ND, not detected, NA, not analyzed, TR, trace amounts (<500 TIC counts). The data underlying this Table can be found at https://doi.org/10.6084/m9.figshare.21692156.v1. (DOCX) [file pbio.3001887.s019.docx]

***Table S4***. Relative amounts (mean ± SE, N=4-5) of volatiles detected at various time periods after inoculation of fresh spruce bark with *E. polonica* (4, 8, and 12 days). Volatiles were collected on polydimethylsiloxane tubes for 2 hours and were subjected to GC-MS analysis (see materials and methods section for details). ND=not detected, NA=not analyzed, TR= trace amounts (<500 TIC counts). The data underlying this Table can be found at https://doi.org/10.6084/m9.figshare.21692156.v1

| ***Compounds*** | **RT^#^** | **F*^$^*** | **P*^$^*** | ***E. polonica* peak area (*10^4^ TIC counts)** | | |
| --- | --- | --- | --- | --- | --- | --- |
|  |  |  |  | **4d** | **8d** | **12d** |
| ***Aliphatics*** |  | | | | | |
| 2-Butanone | 1.85 | - | - | NA | NA | NA |
| 2-Methyl-3-buten-2-ol | 1.93 | - | - | NA | NA | NA |
| Ethyl acetate | 1.95 | - | - | NA | NA | NA |
| Isobutanol | 2.40 | 0.89 | 0.388 | TR | 0.14±0.03 | 0.26±0.08 |
| **Isopropyl acetate** | 2.33 | 27.91 | **<0.001** | 0.05±0.01(b) | 1.17±0.33(b) | 3.43±0.32(a) |
| **Acetoin** | 2.85 | 9.89 | **0.009** | 1.48±0.89(b) | 3.59±1.71(ab) | 10.7±4.04(a) |
| **Ethyl propanoate** | 2.88 | 16.04 | **<0.001** | 3.08±0.72(b) | 18.11±2.91(a) | 12.51±6.25(a) |
| **3-Methyl-1-butanol** | 3.24 | 18.87 | **<0.001** | 1.29±0.32(b) | 5.16±0.96(a) | 5.83±0.91(a) |
| **Ethyl isobutyrate** | 3.69 | 15.63 | **<0.001** | 0.38±0.18(b) | 1.45±0.46(ab) | 3.22±0.04(a) |
| **Isobutyl acetate** | 3.99 | 5.18 | **0.044** | 0.2±0.05(a) | 2.64±0.52(a) | 3.12±1.18(a) |
| 2,3-Butanediol | 4.17 | - | - | ND | ND | 0.05±0.01 |
| Ethyl butanoate | 4.55 | 0.9 | 0.366 | 0.54±0.16 | 10.41±2.08 | 4.22±1.49 |
| Ethyl but-2-enoate | 5.60 | 1.08 | 0.329 | 0.1±0.03 | 1.1±0.24 | 0.6±0.2 |
| Ethyl 2-methylbutyrate | 5.75 | 2.44 | 0.179 | 0.09±0.02 | 0.24±0.09 | ND |
| 1-Hexanol | 6.25 | - | - | ND | ND | ND |
| **3-Methyl-1-butyl acetate** | 6.46 | 12.04 | **0.005** | 22.15±4.34(a) | 14.24±1.55(ab) | 5.73±1.55(b) |
| Isopentyl-2-methylbutanoate | 12.47 | 0.16 | 0.858 | 0.45±0.25 | 0.23±0.11 | 0.15±0.06 |
| Isoamyl valerate | 12.60 | 1.43 | 0.289 | 1.33±0.69 | 0.49±0.28 | 0.18±0.11 |
| Sum |  | 5.13 | **0.02** | 30.64±4.16(b) | 58.91±5.67(a) | 45.26±9.16(ab) |
| ***Aromatics*** |  | | | | | |
| 2-Phenylethyl alcohol | 12.79 | - | - | ND | ND | 0.07±0.01 |
| 2-Phenylethyl acetate | 16.39 | 0.59 | 0.461 | 0.21±0.05 | 0.38±0.11 | 0.29±0.04 |
| Citronellyl acetate | 18.58 | - | - | 0.85±0.18 | ND | 0.06±0.02 |
| Sum |  |  |  | 0.89±0.29 | 0.44±0.18 | 0.23±0.12 |
| ***Spiroketals*** |  | | | | | |
| *endo-*1,3-dimethyl-2,9-dioxabicyclo[3.3.1]nonane | 10.81 | - | - | ND | ND | ND |
| *trans*-Conophthorin | 11.29 | - | - | ND | ND | ND |
| Brevicomin | 11.64 | - | - | ND | ND | ND |
| *exo-*1,3-dimethyl-2,9-dioxabicyclo[3.3.1]nonane | 12.37 | - | - | ND | ND | TR |
| ***Monoterpenes*** |  | | | | | |
| **Santene** | 6.61 | 5.28 | **0.042** | 1.09±0.24(a) | 0.78±0.22(a) | 0.37±0.07(a) |
| **Tricyclene** | 7.67 | 11.03 | **0.007** | 3.55±0.49(a) | 2.46±0.91(ab) | 0.53±0.08(b) |
| **α-Thujene** | 7.76 | 16.15 | **<0.001** | 2.37±0.65(a) | 1.54±0.91(ab) | 0.22±0.11(b) |
| **α-Pinene** | 7.94 | 11.41 | **0.006** | 665±92.58(a) | 444±161.5(ab) | 114±15.67(b) |
| **Camphene** | 8.34 | 24.68 | **<0.001** | 10.86±1.25(a) | 8.37±3.08(a) | 2.04±0.28(b) |
| **Verbenene** | 8.51 | 13.88 | **<0.001** | 0.43±0.06(a) | 0.28±0.09(ab) | 0.07±0.01(b) |
| Sabinene | 9.50 | 1.66 | 0.245 | 0.81±0.41 | 0.37±0.21 | ND |
| **β-Pinene** | 9.13 | 16.24 | **<0.001** | 1186±133(a) | 728±274(ab) | 131±24.02(b) |
| **β-Myrcene** | 9.54 | 11.86 | **0.005** | 32.48±10.99(a) | 21.12±12.68(ab) | 3.02±1.47(b) |
| α-Phellandrene | 9.88 | 1.05 | 0.353 | 1.56±0.45 | 0.79±0.25 | ND |
| α-Terpinene | 10.21 | 1.6 | 0.253 | 0.32±0.13 | 0.35±0.15 | ND |
| ***p*-Cymene** | 10.43 | 9.23 | **0.011** | 18.83±3.69(a) | 11.63±3.88(ab) | 3.93±0.7(b) |
| **Limonene** | 10.51 | 22.34 | **<0.001** | 55.65±10.3(a) | 36.45±16.17(a) | 7.61±1.44(b) |
| **β-Phellandrene** | 10.55 | 13.93 | **<0.001** | 151±36.63(a) | 104±55.09(ab) | 19.93±7.27(b) |
| γ-Terpinene | 11.37 | - | - | 0.92±0.31 | ND | ND |
| α-Terpinolene | 12.16 | 1.29 | 0.308 | 1.07±0.27 | 0.82±0.33 | ND |
| *p*-Cymenene | 12.19 | 0.76 | 0.402 | 0.33±0.08 | 0.26±0.09 | 0.23±0.03 |
| Sum |  | 5.88 | **0.02** | 2128±310(a) | 1360±610(ab) | 283±53.8(b) |
| ***Oxygenatedmonoterpenes*** | | | | | | |
| **1,8-Cineole** | 10.61 | 8.66 | **0.013** | 4.45±0.7(a) | 3.19±0.61(ab) | 1.69±0.44(b) |
| Linalool oxide | 11.73 | - | - | TR | TR | ND |
| Fenchone | 12.15 | 1.68 | 0.222 | 0.52±0.11 | 0.48±0.18 | 0.25±0.1 |
| *trans*-4-Thujanol | 12.42 | - | - | ND | ND | ND |
| ***exo*-Fenchol** | 12.82 | 9.49 | **0.012** | 0.15±0.07(a) | 0.22±0.02(a) | 0.4±0.04(a) |
| Thujone | 12.93 | - | - | 0.04±0 | ND | ND |
| *p*-Isopropylcyclohexanol | 13.41 | - | - | ND | ND | 0.06±0.01 |
| *trans*-Pinocarveol | 13.48 | 3.02 | 0.11 | 0.37±0.16 | 0.49±0.09 | 0.63±0.11 |
| Camphor | 13.63 | 0.11 | 0.751 | 0.21±0.08 | 0.16±0.03 | 0.13±0.03 |
| Camphene hydrate | 13.73 | 0.43 | 0.663 | 0.1±0.05 | 0.12±0.01 | 0.16±0.03 |
| Pinocamphone | 14.43 | 3.39 | 0.093 | 2.5±0.45 | 2.2±0.23 | 1.45±0.09 |
| Pinocarvone | 14.10 | - | - | ND | ND | ND |
| *endo*-Borneol | 14.18 | 1.03 | 0.333 | 3.21±0.6 | 3.26±0.58 | 4.22±0.69 |
| 3-Thujene-2-one | 14.34 | - | - | ND | ND | ND |
| Isopinocamphone | 14.40 | 0.09 | 0.766 | 1.11±0.33 | 0.9±0.05 | 1.03±0.19 |
| Terpinen-4-ol | 14.46 | 0.28 | 0.608 | 0.97±0.43 | 0.64±0.23 | 0.78±0.19 |
| ***p*-Cymene-8-ol** | 14.65 | 29.44 | **<0.001** | 0.15±0.05 | 0.41±0.05 | 1.07±0.26 |
| α-Terpineol | 14.79 | 0.34 | 0.574 | 1.49±0.56 | 1.45±0.11 | 1.86±0.26 |
| **Myrtenol** | 14.94 | 41.22 | **<0.001** | ND | 0.65±0.12(b) | 2.72±0.35(a) |
| Verbenone | 15.28 | - | - | ND | ND | ND |
| 2-Hydroxycineole | 15.58 | - | - | ND | TR | TR |
| **Thymol methyl ether** | 15.85 | 5.65 | **0.037** | 2.37±0.76(a) | 1.52±0.28(a) | 0.83±0.14(a) |
| Myrtanol isomer1 | 16.73 | - | - | ND | ND | ND |
| Myrtanol isomer2 | 16.30 | - | - | ND | ND | ND |
| *p*-Menth-2-en-7-ol | 16.42 | - | - | ND | ND | ND |
| Myrtanol isomer3 | 16.48 | - | - | ND | ND | ND |
| Myrtenyl acetate isomer1 | 17.43 | 0.02 | 0.907 | 0.93±0.34 | 0.83±0.32 | ND |
| Myrtenyl acetate isomer2 | 18.13 | 0.39 | 0.549 | 0.79±0.22 | 0.43±0.1 | 0.68±0.15 |
| Sum |  |  |  | 19.09±3.98 | 16.59±1.06 | 17.66±1.42 |
| ***Sesquiterpenes*** |  | | | | | |
| **α-Longipinene** | 18.63 | 8.67 | **0.013** | 0.57±0.2(a) | 0.19±0.05(ab) | 0.09±0.04(b) |
| **Longicyclene** | 19.10 | 6.52 | **0.027** | 0.64±0.23(a) | 0.23±0.06(a) | 0.11±0.05(a) |
| **Longifolene** | 19.88 | 10.03 | **0.009** | 4.45±1.3(a) | 1.74±0.31(ab) | 1.08±0.26(b) |
| **(*E*)-β-Caryophyllene** | 20.17 | 20.48 | **<0.001** | 12.86±3.85(a) | 4.01±0.21(b) | 2.28±0.47(b) |
| (*E*)-β-Caryophyllene (fungus) | 20.56 | - | - | ND | ND | ND |
| **(*E*)-β-Farnesene** | 20.84 | 4.6 | **0.099** | 0.78±0.15 | 0.16±0.02 | ND |
| **Humulene** | 20.90 | 12.25 | **0.005** | 4.45±1.26(a) | 1.64±0.15(ab) | 0.99±0.1(b) |
| Caryophyllene oxide | 23.56 | - | - | ND | ND | ND |
| Sum |  | 7.44 | **0.01** | 23.58±7.81(a) | 7.89±0.82(ab) | 4.56±1.03(b) |

^#^- Estimated retention time from GC-MS

***^$^-***Significant differences between time points are denoted by small letters (ANOVA, followed by Tukey’s test, *P<0.05)*
