# Supplementary material for: Conifer-killing bark beetles locate fungal symbionts by detecting volatile fungal metabolites of host tree resin monoterpenes
Source: PLoS Biol. 2023 Feb 21;21(2):e3001887. doi: 10.1371/journal.pbio.3001887 (PMC9943021; doi:10.1371/journal.pbio.3001887)
Supplement: S5 Table — Volatiles were collected on polydimethylsiloxane tubes for 2 h and were subjected to GC–MS analysis (see Materials and methods section for details). ND, not detected, NA, not analyzed, TR, trace amounts (<500 TIC counts). The data underlying this Table can be found at https://doi.org/10.6084/m9.figshare.21692156.v1. (DOCX) [file pbio.3001887.s020.docx]

***Table S5***. Relative amounts (mean ± SE, N=5) of volatiles detected at various time periods after inoculation of fresh spruce bark with *G. penicillata* (4, 8, 12 and 18 days). Volatiles were collected on polydimethylsiloxane tubes for 2 hours and were subjected to GC-MS analysis (see materials and methods section for details). ND=not detected, NA=not analyzed, TR= trace amounts (<500 TIC counts). The data underlying this Table can be found at https://doi.org/10.6084/m9.figshare.21692156.v1

| ***Compounds*** | **RT^#^** | **F*^$^*** | **P*^$^*** | **G. penicillata peak area (*10^4^ TIC counts)** | | | |
| --- | --- | --- | --- | --- | --- | --- | --- |
|  |  |  |  | **4d** | **8d** | **12d** | **18d** |
| ***Aliphatics*** |  | | | | | | |
| 2-Butanone | 1.85 | 0.01 | 0.914 | 4.02±0.94 | 3.8±0.81 | 6.83±3.55 | 1±0.2 |
| **2-Methyl-3-buten-2-ol** | 1.93 | 12.6 | **0.004** | 0.1±0.04(b) | 0.2±0.04(b) | 0.57±0.14(ab) | 0.86±0.18(a) |
| Ethyl acetate | 1.95 | 0.67 | 0.425 | 4.33±0.73 | 9.41±2.76 | 9.39±2.35 | 6.16±3.02 |
| Isobutanol | 2.40 | 0.81 | 0.386 | 0.51±0.07 | 1.11±0.19 | 1.24±0.03 | 0.66±0.28 |
| Isopropyl acetate | 2.33 | - | - | ND | ND | ND | ND |
| Acetoin | 2.85 | 0.6 | 0.449 | 5.21±2.25 | 5.06±0.54 | 10.21±3.15 | 8.16±5.12 |
| Ethyl propanoate | 2.88 | 1.97 | 0.184 | 1.79±0.6 | 0.3±0.11 | 0.25±0.09 | 0.34±0.13 |
| 3-Methyl-1-butanol | 3.24 | 0.03 | 0.871 | 4.52±0.79 | 12.8±0.37 | 8.47±2.56 | 5.11±2.46 |
| Ethyl isobutyrate | 3.69 | 2.57 | 0.184 | 0.77±0.13 | 0.35±0.08 | ND | ND |
| Isobutyl acetate | 3.99 | 0.07 | 0.8 | 0.29±0.07 | 1.19±0.37 | 0.93±0.42 | 0.65±0.35 |
| 2,3-Butanediol | 4.17 | 1.11 | 0.323 | ND | 0.09±0.01 | 0.23±0.03 | 0.16±0.04 |
| Ethyl butanoate | 4.55 | - | - | 0.49±0.21 | ND | ND | ND |
| Ethyl but-2-enoate | 5.60 | 0.14 | 0.721 | 0.25±0.06 | 0.38±0.03 | 0.5±0.07 | 0.29±0 |
| Ethyl 2-methylbutyrate | 5.75 | - | - | 0.09±0.02 | ND | ND | ND |
| 1-Hexanol | 6.25 | 0.7 | 0.422 | 0.19±0.06 | 0.78±0.28 | 0.83±0.46 | ND |
| **3-Methyl-1-butyl acetate** | 6.46 | 6.19 | **0.027** | 0.43±0.12(a) | 1.55±0.49(a) | 1.28±0.19(a) | 1.34±0.14(a) |
| Isopentyl-2-methylbutanoate | 12.47 | 0.6 | 0.594 | 0.13±0.04 | 0.08±0.02 | 0.05±0.01 | ND |
| Isoamyl valerate | 12.60 | 1.28 | 0.338 | 0.37±0.11 | 0.17±0.07 | 0.11±0.03 | 0.08±0.03 |
| Sum |  |  |  | 23.77±2.89 | 33.4±5.36 | 37±5.29 | 16.5±6.96 |
| ***Aromatics*** |  | | | | | | |
| 2-Phenylethyl alcohol | 12.79 | 2.19 | 0.167 | ND | 0.73±0.11 | 0.94±0.17 | 1.19±0.25 |
| 2-Phenylethyl acetate | 16.39 | 0.33 | 0.577 | 0.19±0.05 | 0.34±0.11 | 0.07±0.02 | ND |
| Citronellyl acetate | 18.58 | - | - | ND | ND | ND | ND |
| Sum |  |  |  | 0.19±0.06 | 0.91±0.21 | 0.98±0.19 | 0.93±0.44 |
| ***Spiroketals*** |  | | | | | | |
| *endo-*1,3-dimethyl-2,9-dioxabicyclo[3.3.1]nonane | 10.81 | 0.4 | 0.537 | 0.2±0.07 | 0.12±0.04 | 0.07±0.01 | 0.11±0.02 |
| *trans*-Conophthorin | 11.29 | 1.9 | 0.191 | 0.03±0 | 0.9±0.46 | 0.84±0.37 | 0.75±0.32 |
| Brevicomin | 11.64 |  |  | ND | ND | ND | ND |
| ***exo-*1,3-dimethyl-2,9-dioxabicyclo[3.3.1]nonane** | 12.37 | 7.1 | **0.017** | 1.91±0.48(a) | 1.02±0.28(a) | 0.54±0.1(a) | 0.52±0.22(a) |
| Sum |  |  |  | 2.17±0.76 | 2.01±0.41 | 1.44±0.48 | 0.57±0.23 |
| ***Monoterpenes*** |  | | | | | | |
| Santene | 6.61 | 1.83 | 0.194 | 0.96±0.25 | 0.54±0.18 | 0.42±0.09 | 0.49±0.11 |
| **Tricyclene** | 7.67 | 25.75 | **<0.001** | 3.31±0.88(a) | 0.9±0.31(ab) | 0.4±0.11(b) | 0.27±0.09(b) |
| **α-Thujene** | 7.76 | 26.45 | **<0.001** | 5.41±2.65(a) | 1.33±0.6(ab) | 0.43±0.16(b) | 0.18±0.06(b) |
| **α-Pinene** | 7.94 | 42.14 | **<0.001** | 683±205(a) | 193±69(ab) | 82.4±23.45(ab) | 36.17±10(c) |
| **Camphene** | 8.34 | 20.99 | **<0.001** | 11.08±2.91(a) | 3.01±0.89(ab) | 1.35±0.45(b) | 1.16±0.44(b) |
| **Verbenene** | 8.51 | 14.53 | **0.001** | 0.79±0.45(a) | 0.14±0.05(ab) | 0.06±0.01(b) | 0.05±0.01(b) |
| Sabinene | 9.50 |  |  | 0.32±0.03 | ND | ND | ND |
| **β-Pinene** | 9.13 | 136.93 | **<0.001** | 800±108(a) | 159±19.35(b) | 45.92±9.33(c) | 13.28±4.35(d) |
| **β-Myrcene** | 9.54 | 140.32 | **<0.001** | 22.45±4.99(a) | 6.26±1.06(b) | 1.98±0.23(c) | 0.77±0.07© |
| **Unknown** | 9.85 | 7.3 | **0.003** | 3.57±0.95(a) | 0.27±0.07(b) | 0.28±0.18(b) | 0.91±0.34(ab) |
| **α-Phellandrene** | 9.88 | 13.53 | **0.002** | 1.82±0.53(a) | 0.8±0.35(ab) | 0.28±0.13(b) | 0.27±0.14(b) |
| **α-Terpinene** | 10.21 | 9.77 | **0.007** | 0.6±0.26(a) | 0.25±0.11(ab) | 0.09±0.02(ab) | 0.07±0.02(b) |
| *p*-Cymene | 10.43 | 2.2 | 0.156 | 42.27±20.53 | 24.38±11.35 | 13.31±5.88 | 16.3±6.77 |
| **Limonene** | 10.51 | 10.33 | **0.005** | 56.38±19.04(a) | 19.68±7.25(ab) | 10.11±3.55(b) | 10.27±3.71(b) |
| **β-Phellandrene** | 10.55 | 25.35 | **<0.001** | 180±51.67(a) | 70.17±28.06(ab) | 25.49±9.81(b) | 17.26±6.77(b) |
| **1,8-Cineole** | 10.61 | 40.56 | **<0.001** | 7.18±1.06(a) | 3.57±0.45(ab) | 1.38±0.3(bc) | 0.79±0.23(c) |
| **γ-Terpinene** | 11.37 | 11.51 | **0.004** | 2.43±1.15(a) | 1.32±0.4(ab) | 0.3±0.08(b) | 0.33±0.08(ab) |
| **Sum** |  | 11.55 | **<0.001** | 1503±292(a) | 997±528(ab) | 183±49.9(bc) | 101±35.4(c) |
| ***Oxygenated monoterpenes*** |  | | | | | | |
| **Linalool oxide** | 11.73 | 13.25 | **0.002** | 0.19±0.06(b) | 0.4±0.08(ab) | 1.06±0.34(a) | 1.38±0.38(a) |
| **Fenchone** | 12.15 | 10.96 | **0.004** | 2.2±1.04(b) | 2.63±1.33(b) | 10.74±5.68(b) | 24.42±4.58(a) |
| α-Terpinolene | 12.16 | 1.34 | 0.263 | 6.06±4.09 | 2.8±1.43 | 1.02±0.29 | 1.61±0.52 |
| *p*-Cymenene | 12.19 | 0.72 | 0.407 | 2.07±1.22 | 2.14±0.85 | 2.92±1.77 | 5.07±3 |
| *trans*-4-Thujanol | 12.42 | - | - | ND | ND | ND | TR |
| *exo*-Fenchol | 12.82 | 2.7 | 0.119 | 0.67±0.45 | 1.44±0.55 | 1.78±0.36 | 1.42±0.63 |
| Thujone | 12.93 | 2.8 | 0.123 | 0.11±0.03 | 0.07±0.02 | 0.05±0 | 0.05±0.01 |
| *p*-Isopropylcyclohexanol | 13.41 | 4.1 | 0.064 | 0.32±0.12 | 0.23±0.09 | 0.43±0.17 | 1.11±0.4 |
| ***trans*-Pinocarveol** | 13.48 | 7.18 | **0.016** | 0.59±0.2(a) | 1.44±0.29(a) | 2.68±0.48(a) | 2.36±0.66(a) |
| **Camphor** | 13.63 | 13.06 | **0.002** | 21.83±6.85(b) | 21.02±7.15(b) | 74.89±32.59(ab) | 148±6.25(a) |
| Camphene hydrate | 13.73 | 0.78 | 0.524 | 0.25±0.14 | 0.38±0.14 | 0.27±0.08 | 0.33±0.07 |
| Pinocamphone | 14.43 | 0.13 | 0.727 | 4.52±0.61 | 3.51±0.66 | 3.15±0.82 | 4.07±0.91 |
| Pinocarvone | 14.10 | - | - | ND | ND | ND | ND |
| endo-Borneol | 14.18 | 1.29 | 0.275 | 4.34±0.61 | 9.52±1.35 | 11.67±4.18 | 6.46±2.68 |
| 3-Thujene-2-one | 14.34 | - | - | ND | ND | ND | ND |
| Isopinocamphone | 14.40 | 3.72 | 0.071 | 3.12±1.07(b) | 2.97±1.01(ab) | 5.43±2.12(a) | 10.59±2.84(a) |
| Terpinen-4-ol | 14.46 | 0.59 | 0.455 | 23.53±16.33(a) | 56.28±22.27(a) | 56.52±19.3(a) | 47.27±14.19(a) |
| ***p*-Cymene-8-ol** | 14.65 | 12.45 | **0.003** | 1.02±0.51(b) | 2.52±0.63(ab) | 2.99±0.48(ab) | 3.84±0.52(a) |
| α-Terpineol | 14.79 | 3.96 | 0.063 | 7.6±2.93 | 12.84±4.13 | 20.09±5.84 | 20.15±4.63 |
| **Myrtenol** | 14.94 | 16.37 | **0.001** | 4.42±1.22(b) | 15.32±2.87(a) | 24.84±5.61(a) | 35.57±10.83(a) |
| Verbenone | 15.28 | 0.18 | 0.679 | ND | 0.04±0.01 | 0.05±0 | 0.03±0.01 |
| **2-Hydroxycineole** | 15.58 | 10.48 | **0.008** | 0.05±0.01(b) | 0.11±0.01(ab) | 0.18±0.05(ab) | 0.25±0.03(a) |
| **Thymol methyl ether** | 15.85 | 7.33 | **0.015** | 2.88±0.76(a) | 2.04±0.67(a) | 1.25±0.46(a) | 1.09±0.43(a) |
| **Myrtanol isomer1** | 16.73 | 14.04 | **0.005** | ND | 0.05±0.01(b) | 0.16±0.06(ab) | 0.28±0.06(a) |
| **Myrtanol isomer2** | 16.30 | 18.09 | **0.001** | 0.04±0.01(c) | 0.1±0.03(bc) | 0.38±0.14(ab) | 0.79±0.18(a) |
| *p*-Menth-2-en-7-ol | 16.42 | - | - | ND | TR | TR | TR |
| **Myrtanol isomer3** | 16.48 | 10.12 | **0.011** | ND | 0.19±0.05(b) | 0.61±0.13(ab) | 1.03±0.36(a) |
| **Myrtenyl acetate isomer1** | 17.43 | 8.37 | **0.02** | 1.9±0.46(a) | 0.13±0.03(a) | 0.27±0.14(a) | ND |
| Myrtenyl acetate isomer2 | 18.13 | - | - | ND | ND | 0.05±0 | ND |
| **Sum** |  | 5.51 | **0.01** | 53.6±8.02(b) | 151±37.9(ab) | 220±69.1(ab) | 332±37.9(a) |
| ***Sesquiterpenes*** |  | | | | | | |
| α-Longipinene | 18.63 | 3.23 | 0.097 | 0.39±0.16 | 0.15±0.08 | 0.1±0.04 | 0.11±0.04 |
| **Longicyclene** | 19.10 | 9.4 | **0.007** | 0.44±0.16(a) | 0.23±0.11(a) | 0.11±0.06(a) | 0.09±0.05(a) |
| **Longifolene** | 19.88 | 9.65 | **0.006** | 2.96±0.86(a) | 1.69±0.73(ab) | 0.85±0.48(ab) | 0.69±0.41(b) |
| **(*E*)-β-Caryophyllene** | 20.17 | 18.57 | **<0.001** | 9.7±2.05(a) | 5.52±1.62(ab) | 2.2±0.94(b) | 1.87±0.84(b) |
| (*E*)-β-Caryophyllene (fungus) | 20.56 | 0 | 0.992 | ND | 0.51±0.15 | 0.44±0.15 | 0.8±0.43 |
| **(*E*)-β-Farnesene** | 20.84 | 12.11 | **0.01** | 0.36±0.05(a) | 0.12±0.02(b) | ND | ND |
| **Humulene** | 20.90 | 10.57 | **0.005** | 3±0.5(a) | 1.94±0.59(ab) | 1.05±0.41(ab) | 0.97±0.39(b) |
| Caryophyllene oxide | 23.56 | - | - | ND | 0.7±0.12 | 0.66±0.12 | 0.86±0.18 |
| Sum |  |  |  | 13.4±2.74 | 14.8±4.85 | 5.28±2.17 | 9.97±1.84 |

^#^- Estimated retention time from GC-MS

***^$^-***Significant differences between time points are denoted by small letters (ANOVA, followed by Tukey’s test, *P<0.05)*
