# Supplementary material for: Conifer-killing bark beetles locate fungal symbionts by detecting volatile fungal metabolites of host tree resin monoterpenes
Source: PLoS Biol. 2023 Feb 21;21(2):e3001887. doi: 10.1371/journal.pbio.3001887 (PMC9943021; doi:10.1371/journal.pbio.3001887)
Supplement: S6 Table — Volatiles were collected on polydimethylsiloxane tubes for 2 h and were subjected to GC–MS analysis (see Materials and methods section for details). ND, not detected, NA, not analyzed, TR, trace amounts (<500 TIC counts). The data underlying this Table can be found at https://doi.org/10.6084/m9.figshare.21692156.v1. (DOCX) [file pbio.3001887.s021.docx]

***Table S6.*** Relative amounts (mean ± SE, N=5) of volatiles detected at various time periods after inoculation of fresh spruce bark with *L. europhioides* (4, 8, 12 and 18 days). Volatiles were collected on polydimethylsiloxane tubes for 2 hours and were subjected to GC-MS analysis (see materials and methods section for details). ND=not detected, NA=not analyzed, TR= trace amounts (<500 TIC counts). The data underlying this Table can be found at https://doi.org/10.6084/m9.figshare.21692156.v1

| ***Compounds*** | **RT^#^** | **F*^$^*** | **P*^$^*** | ***L. europhioides* peak area (*10^4^ TIC counts)** | | | |
| --- | --- | --- | --- | --- | --- | --- | --- |
|  |  |  |  | **4d** | **8d** | **12d** | **18d** |
| ***Aliphatics*** |  | | | | | | |
| **2-Butanone** | 1.85 | 5.86 | **0.027** | 3.06±0.69(a) | NA | 2.27±1.13(a) | 3.04±0.65(a) |
| **2-Methyl-3-buten-2-ol** | 1.93 | 42.57 | **<0.001** | 0.59±0.09(b) | 4.35±2.05(a) | 11.13±3.29(a) | 13.82±2.62(a) |
| **Ethyl acetate** | 1.95 | 14.65 | **0.002** | 3.38±0.79(a) | 0.57±0.34(ab) | 0.21±0.11(b) | 0.23±0.14(b) |
| Isobutanol | 2.40 | 1.64 | 0.227 | 2.5±0.23(ab) | 5.94±1.52(a) | 1.13±0.39(b) | TR |
| Isopropyl acetate | 2.33 | - | - | ND | ND | ND | ND |
| Acetoin | 2.85 | 4.48 | 0.051 | 12.69±3.21 | 8.92±2.51 | 7.86±3.25 | 2.39±1.2 |
| Ethyl propanoate | 2.88 | 0.03 | 0.877 | 0.27±0.16 | TR | ND | 0.13±0.03 |
| **3-Methyl-1-butanol** | 3.24 | 13.94 | **0.002** | 14.04±1.12(a) | 20.8±1.32(ab) | 8.66±3.5(bc) | 1.02±0.3 (c) |
| Ethyl isobutyrate | 3.69 | - | - | 0.62±0.3 | ND | ND | ND |
| Isobutyl acetate | 3.99 | 2.11 | 0.18 | 0.54±0.14 | 0.38±0.04 | 0.26±0.06 | ND |
| 2,3-Butanediol | 4.17 | 0.44 | 0.52 | 0.11±0 | 0.09±0.03 | 0.16±0.06 | 0.15±0.05 |
| Ethyl butanoate | 4.55 | - | - | ND | ND | ND | ND |
| Ethyl but-2-enoate | 5.60 | 0.05 | 0.831 | 0.78±0.09 | 1.4±0.21 | 0.54±0.23 | ND |
| Ethyl 2-methylbutyrate | 5.75 | - | - | ND | ND | ND | ND |
| **1-Hexanol** | 6.25 | 8.81 | **0.021** | 1.07±0.17(b) | 1.95±0.18(a) | ND | ND |
| 3-Methyl-1-butyl acetate | 6.46 | 4.26 | 0.061 | 0.16±0.03(b) | 0.24±0.04(b) | 0.2±0.1(ab) | 1.19±0.11(a) |
| Isopentyl-2-methylbutanoate | 12.47 | - | - | 1.08±0.46 | 0.71±0.3 | ND | ND |
| Isoamyl valerate | 12.60 | 0.13 | 0.88 | 1.02±0.8 | 1.12±0.58 | ND | ND |
| Sum |  |  |  | 40.9±6.61 | 42.59±4.1 | 27.61±7.66 | 20.93±2.82 |
| ***Aromatics*** |  | | | | | | |
| 2-Phenylethyl alcohol | 12.79 | 1.13 | 0.303 | 0.61±0.11 | 1.64±0.3 | 1.2±0.14 | 1.22±0.18 |
| 2-Phenylethyl acetate | 16.39 | - | - | ND | ND | ND | ND |
| Citronellyl acetate | 18.58 | - | - | ND | ND | ND | ND |
| **Sum** |  | 3.88 | **0.03** | 0.61±0.12(b) | 1.64±0.33(a) | 1.19±0.18(ab) | 1.22±0.2(ab) |
| ***Spiroketals*** |  | | | | | | |
| *endo-*1,3-dimethyl-2,9-dioxabicyclo[3.3.1]nonane | 10.81 | 4.06 | 0.061 | 1.31±0.24 | 1.02±0.2 | 0.78±0.12 | 0.75±0.12 |
| ***trans*-Conophthorin** | 11.29 | 69.34 | **<0.001** | 0.03±0(c) | 0.05±0(b) | 0.06±0.01(b) | 0.15±0.02(a) |
| Brevicomin | 11.64 | - | - | TR | TR | ND | TR |
| ***exo-*1,3-dimethyl-2,9-dioxabicyclo[3.3.1]nonane** | 12.37 | 3.77 | **0.07** | 2.28±0.37(a) | 1.77±0.29(a) | 1.19±0.04(a) | 1.52±0.12(a) |
| **Sum** |  | 4 | **0.02** | 3.61±0.49(a) | 2.86±0.43(ab) | 1.63±0.43(b) | 2.44±0.15(ab) |
| ***Monoterpenes*** |  | | | | | | |
| Santene | 6.61 | 1.83 | 0.194 | 0.97±0.27 | 0.93±0.17 | 0.48±0.16 | 0.46±0.05 |
| **Tricyclene** | 7.67 | 48.16 | **<0.001** | 3.15±0.46(a) | 0.89±0.26(b) | 0.31±0.12(bc) | 0.14±0.03(c) |
| **α-Thujene** | 7.76 | 21.98 | **<0.001** | 3.17±1.01(a) | 0.73±0.33(ab) | 0.18±0.11(b) | 0.11±0.06(b) |
| **α-Pinene** | 7.94 | 56.3 | **<0.001** | 633±111(a) | 189±46.47(b) | 60.23±18.76(bc) | 33±7.3(c) |
| **Camphene** | 8.34 | 49.67 | **<0.001** | 10.4±1.71(a) | 3.7±1.03(b) | 1.33±0.41(bc) | 0.72±0.19(c) |
| **Verbenene** | 8.51 | 22.45 | **<0.001** | 0.44±0.05(a) | 0.15±0.05(ab) | 0.06±0.02(b) | 0.05±0.01(b) |
| Sabinene | 9.50 | - | - | 0.22±0.07 | ND | ND | ND |
| **β-Pinene** | 9.13 | 89.32 | **<0.001** | 907±161(a) | 208±58.89(b) | 45.89±13.38(c) | 14.74±3.6(c) |
| **β-Myrcene** | 9.54 | 22.04 | **<0.001** | 28.05±11.54(a) | 10.09±4.59(ab) | 1.68±0.96(bc) | 1.28±0.9(c) |
| **Unknown** | 9.85 | 4.56 | **0.02** | 3.92±1.26(a) | 1.88±0.63(ab) | 0.66±0.26(ab) | 0.36±0.14(b) |
| **α-Phellandrene** | 9.88 | 4.94 | **0.05** | 1.34±0.4(a) | 0.87±0.27(a) | 0.12±0.04(a) | ND |
| α-Terpinene | 10.21 | 3 | 0.118 | 0.32±0.1 | 0.28±0.13 | 0.05±0.01 | ND |
| ***p*-Cymene** | 10.43 | 18.43 | **<0.001** | 30.27±6.76(a) | 11.94±2.98(ab) | 4.54±1.74(b) | 4.15±1.55(b) |
| **Limonene** | 10.51 | 27.95 | **<0.001** | 39.56±12.8(a) | 17.23±6.19(ab) | 4.67±2.18(bc) | 2.66±1.37(c) |
| **β-Phellandrene** | 10.55 | 22.57 | **<0.001** | 169±63.07(a) | 70.29±30.68(ab) | 13.38±6.65(bc) | 10.7±7.03(c) |
| γ-Terpinene | 11.37 | 3.2 | 0.099 | 1.25±0.34(a) | 0.99±0.45(ab) | 0.28±0.17(b) | 0.39±0.17(ab) |
| α-Terpinolene | 12.16 | 2.39 | 0.141 | 2.84±1.09 | 3.41±1.7 | 1.17±0.73 | 1.37±0.9 |
| *p*-Cymenene | 12.19 | 0.56 | 0.464 | 1.44±0.44 | 1.38±0.28 | 1.25±0.5 | 1.92±0.59 |
| **Sum** |  | 12.19 | **<0.001** | 1828±409(a) | 517±168(b) | 134±49.1(b) | 69.5±24.5(b) |
| ***Oxygenated monoterpenes*** |  | | | | | | |
| **1,8-Cineole** | 10.61 | 15.84 | **0.001** | 3.43±0.9(a) | 1.92±0.75(ab) | 0.35±0.04(b) | 0.72±0.4(b) |
| **Linalool oxide** | 11.73 | 4.17 | **0.058** | 0.3±0.08(a) | 0.81±0.16(a) | 0.6±0.08(a) | 0.97±0.31(a) |
| **Fenchone** | 12.15 | 13.12 | **0.002** | 1.97±0.47(b) | 2.15±0.43(b) | 4.99±1.82(b) | 16.14±2.56(a) |
| *trans*-4-Thujanol | 12.42 | - | - | ND | ND | ND | TR |
| ***exo*-Fenchol** | 12.82 | 4.99 | **0.04** | 1.47±0.27(b) | 3.13±0.56(ab) | 3.03±0.79(ab) | 3.21±0.32(a) |
| Thujone | 12.93 | 1.77 | 0.21 | 0.06±0.02 | 0.05±0.02 | 0.03±0 | 0.03±0 |
| *p*-Isopropylcyclohexanol | 13.41 | 0.08 | 0.785 | 0.48±0.12 | 0.9±0.13 | 0.94±0.27 | 1.04±0.68 |
| ***trans*-Pinocarveol** | 13.48 | 14.4 | **0.003** | 1.08±0.08(a) | 1.27±0.18(a) | 0.05±0.01(b) | ND |
| **Camphor** | 13.63 | 69.66 | **<0.001** | 10.13±1.79(c) | 57.6±6.22(b) | 73.08±17.48(ab) | 110±11.07(a) |
| Camphene hydrate | 13.73 | 1.26 | 0.324 | 0.41±0.09 | 0.74±0.14 | 0.42±0.15 | 0.61±0.11 |
| Pinocamphone | 14.43 | 0.83 | 0.375 | 4.84±0.4 | 4.44±0.41 | 3.58±0.87 | 6.1±0.56 |
| Pinocarvone | 14.10 | - | - | ND | ND | ND | ND |
| ***endo*-Borneol** | 14.18 | 94.37 | **<0.001** | 22.06±1.38(a) | 18.23±4.28(a) | 1.46±0.27(b) | 0.25±0.03(c) |
| 3-Thujene-2-one | 14.34 | 2.37 | 0.143 | 0.36±0.06 | 0.78±0.15 | 0.63±0.05 | 0.7±0.06 |
| **Isopinocamphone** | 14.40 | 29.4 | **<0.001** | 5.84±0.87(b) | 16.38±1.86(ab) | 22.14±2.88(a) | 25.91±3.26(a) |
| Terpinen-4-ol | 14.46 | 0.24 | 0.632 | 1.37±0.63 | 7.5±3.74 | 0.96±0.33 | ND |
| ***p*-Cymene-8-ol** | 14.65 | 5.46 | **0.033** | 1.34±0.3(a) | 1.87±0.32(a) | 1.72±0.5(a) | 2.71±0.57(a) |
| α-Terpineol | 14.79 | 0.35 | 0.563 | 10.21±1.21 | 16.17±3.82 | 13.92±4.02 | 7.96±1.35 |
| Myrtenol | 14.94 | 1.03 | 0.325 | 11.75±1.51 | 23.25±3.76 | 19.22±5.36 | 18.48±3.96 |
| Verbenone | 15.28 | 2.05 | 0.176 | 0.15±0.04 | 0.14±0.06 | 0.15±0.03 | 0.33±0.14 |
| **2-Hydroxycineole** | 15.58 | 16.43 | **0.001** | 0.1±0.01(b) | 0.35±0.05(a) | 0.3±0.04(ab) | 0.48±0.03(a) |
| **Thymol methyl ether** | 15.85 | 6.37 | **0.022** | 2.79±0.94(a) | 2.25±0.94(a) | 0.57±0.18(a) | 1.2±0.72(a) |
| **Myrtanol isomer1** | 16.73 | 12.26 | **0.005** | ND | 0.1±0.02(b) | 0.25±0.05(ab) | 0.38±0.08(a) |
| **Myrtanol isomer2** | 16.30 | 59.93 | **<0.001** | 0.09±0.02(b) | 0.28±0.06(b) | 0.68±0.06(a) | 1.02±0.16(a) |
| *p*-Menth-2-en-7-ol | 16.42 | - | - | TR | TR | TR | 0.05±0 |
| **Myrtanol isomer3** | 16.48 | 51.63 | **<0.001** | 0.13±0.03(c) | 0.48±0.11(bc) | 1.06±0.1(ab) | 1.62±0.25(a) |
| Myrtenyl acetate isomer1 | 17.43 | - | - | 0.52±0.21 | ND | ND | ND |
| Myrtenyl acetate isomer2 | 18.13 | - | - | ND | ND | ND | ND |
| **Sum** |  | 4 | **0.02** | 80.74±7.25(b) | 160±24.3(ab) | 144±36.5(ab) | 200±15.1(a) |
| ***Sesquiterpenes*** |  | | | | | | |
| **α-Longipinene** | 18.63 | 8.58 | **0.012** | 0.52±0.24(a) | 0.32±0.15(a) | 0.05±0.01(a) | 0.07±0.03(a) |
| **Longicyclene** | 19.10 | 4.61 | **0.049** | 0.62±0.28(a) | 0.37±0.19(a) | 0.07±0.02(a) | 0.16±0.08(a) |
| **Longifolene** | 19.88 | 6.88 | **0.018** | 3.54±1.3(a) | 2.91±1.3(a) | 0.58±0.23(a) | 1.09±0.65(a) |
| **(*E*)-β-Caryophyllene** | 20.17 | 7.18 | **0.016** | 11.02±3.28(a) | 8.75±3.2(a) | 2.15±0.73(a) | 3.1±1.52(a) |
| (*E*)-β-Caryophyllene (fungus) | 20.56 | 2.36 | 0.144 | 3.09±0.51 | 6.9±1.21 | 9.33±1.7 | 6.37±1.45 |
| (*E*)-β-Farnesene | 20.84 | 1.76 | 0.212 | 0.34±0.07 | 0.43±0.1 | 0.16±0.02 | 0.18±0.03 |
| Humulene | 20.90 | 3.98 | 0.062 | 3.77±0.94 | 3.2±1.11 | 1.06±0.35 | 1.67±0.79 |
| Caryophyllene oxide | 23.56 | - | - | ND | 0.25±0.05 | ND | ND |
| Sum |  |  |  | 22.89±7.16 | 22.86±7.44 | 11.39±3.71 | 12.5±4.95 |

^#^- Estimated retention time from GC-MS

***^$^-***Significant differences between time points are denoted by small letters (ANOVA, followed by Tukey’s test, *P<0.05)*
