# Supplementary material for: Conifer-killing bark beetles locate fungal symbionts by detecting volatile fungal metabolites of host tree resin monoterpenes
Source: PLoS Biol. 2023 Feb 21;21(2):e3001887. doi: 10.1371/journal.pbio.3001887 (PMC9943021; doi:10.1371/journal.pbio.3001887)
Supplement: S7 Table — Volatiles were collected on polydimethylsiloxane tubes for 2 h and were subjected to GC–MS analysis (see Materials and methods section for details). ND, not detected, NA, not analyzed, TR, trace amounts (<500 TIC counts). The data underlying this Table can be found at https://doi.org/10.6084/m9.figshare.21692156.v1. (DOCX) [file pbio.3001887.s022.docx]

***Table S7***. Relative amounts (mean ± SE, N=5) of volatiles detected at various time periods after inoculation of fresh spruce bark with *O. bicolor* (4, 8, 12 and 18 days). Volatiles were collected on polydimethylsiloxane tubes for 2 hours and were subjected to GC-MS analysis (see materials and methods section for details). ND=not detected, NA=not analyzed, TR= trace amounts (<500 TIC counts). The data underlying this Table can be found at https://doi.org/10.6084/m9.figshare.21692156.v1

| ***Compounds*** | **RT^#^** | **F*^$^*** | **P*^$^*** | ***O. bicolor* peak area (*10^4^ TIC counts)** | | | |
| --- | --- | --- | --- | --- | --- | --- | --- |
|  |  |  |  | **4d** | **8d** | **12d** | **18d** |
| ***Aliphatics*** |  | | | | | | |
| 2-Butanone | 1.85 | 0.25 | 0.621 | 3.05±0.82 | 2.58±0.33 | 2.83±0.74 | 2.82±1.2 |
| 2-Methyl-3-buten-2-ol | 1.93 | - | - | ND | ND | ND | ND |
| **Ethyl acetate** | 1.95 | 1.2 | 0.291 | 0.81±0.29 | 1.43±0.15 | 1.76±0.48 | 1.68±0.49 |
| **Isobutanol** | 2.40 | 13.83 | **0.002** | 0.5±0.119(b) | 1.92±0.2(a) | 2.37±0.3(a) | 1.34±0.51(a) |
| Isopropyl acetate | 2.33 | - | - | ND | ND | ND | ND |
| **Acetoin** | 2.85 | 26.35 | **<0.001** | 6.06±1.7(b) | 6.18±1.55(b) | 12.23±1.9(ab) | 25.51±4.6(a) |
| Ethyl propanoate | 2.88 | 3.64 | 0.079 | 1.17±0.45 | 0.51±0.25 | 0.2±0.07 | 0.08±0.01 |
| **3-Methyl-1-butanol** | 3.24 | 26.82 | **<0.001** | 7.49±0.85(b) | 18.51±0.81(a) | 26.51±1.21(a) | 27.05±3.47(a) |
| Ethyl isobutyrate | 3.69 | 1.21 | 0.3 | 1.23±0.39 | 2.23±0.86 | 0.29±0.09 | ND |
| **Isobutyl acetate** | 3.99 | 8.12 | **0.019** | ND | 0.17±0.01(b) | 0.17±0.03(b) | 0.46±0.03(a) |
| 2,3-Butanediol | 4.17 | 2.51 | 0.137 | 0.05±0.01 | 0.29±0.1 | 0.16±0.03 | 0.32±0.09 |
| Ethyl butanoate | 4.55 | 3.46 | 0.09 | 0.28±0.08 | 0.26±0.09 | 0.1±0.02 | 0.08±0.02 |
| Ethyl but-2-enoate | 5.60 | 0.74 | 0.409 | 0.3±0.05 | 0.6±0.22 | 0.25±0.02 | 0.21±0.01 |
| Ethyl 2-methylbutyrate | 5.75 | 0.45 | 0.527 | 0.18±0.07 | 0.27±0.08 | ND | ND |
| 1-Hexanol | 6.25 | 0.33 | 0.575 | 0.25±0.06 | 0.56±0.05 | 0.32±0.15 | 0.11±0.04 |
| **3-Methyl-1-butyl acetate** | 6.46 | 12.07 | **0.003** | 0.06±0.01(b) | 0.19±0.03(ab) | 0.2±0.03(a) | 0.35±0.12(a) |
| Isopentyl-2-methylbutanoate | 12.47 | 0.7 | 0.583 | 0.13±0.04 | 0.05±0.01 | 0.08±0.02 | 0.1±0.04 |
| Isoamyl valerate | 12.60 | 0.86 | 0.5 | 0.41±0.14 | 0.31±0.12 | 0.19±0.07 | 0.23±0.1 |
| **Sum** |  | 9.49 | **<0.001** | 21.65±2.04(c) | 35.06±3.25(bc) | 46.99±3.37(ab) | 58.75±8.75(a) |
| ***Aromatics*** |  | | | | | | |
| 2-Phenylethyl alcohol | 12.79 | 0.97 | 0.341 | 0.34±0.09 | 0.55±0.12 | 0.64±0.1 | 0.58±0.15 |
| 2-Phenylethyl acetate | 16.39 | - | - | ND | ND | ND | ND |
| Citronellyl acetate | 18.58 | - | - | ND | ND | ND | ND |
| Sum |  |  |  | 0.2±0.11 | 0.55±0.13 | 0.65±0.11 | 0.58±0.16 |
| ***Spiroketals*** |  | | | | | | |
| *endo-*1,3-dimethyl-2,9-dioxabicyclo[3.3.1]nonane | 10.81 | 1.65 | 0.217 | 0.3±0.08 | 0.47±0.09 | 0.48±0.08 | 0.46±0.06 |
| *trans*-Conophthorin | 11.29 | - | - | ND | ND | ND | ND |
| Brevicomin | 11.64 | - | - | ND | ND | TR | TR |
| *exo-*1,3-dimethyl-2,9-dioxabicyclo[3.3.1]nonane | 12.37 | 3.63 | 0.074 | 0.41±0.07 | 1.01±0.17 | 0.85±0.12 | 0.91±0.13 |
| **Sum** |  | 16.58 | **<0.001** | 0.7±0.07(b) | 1.48±0.1(a) | 1.22±0.01(a) | 1.38±0.09(a) |
| ***Monoterpenes*** |  | | | | | | |
| **Santene** | 6.61 | 19.19 | **<0.001** | 1.08±0.29(a) | 0.72±0.08(ab) | 0.41±0.06(b) | 0.36±0.02(b) |
| **Tricyclene** | 7.67 | 48.9 | **<0.001** | 4.35±0.82(a) | 1.69±0.26(a) | 0.59±0.19(b) | 0.35±0.07(b) |
| **α-Thujene** | 7.76 | 38.7 | **<0.001** | 2.15±0.61(a) | 0.73±0.16(ab) | 0.25±0.12(bc) | 0.12±0.05(c) |
| **α-Pinene** | 7.94 | 71.46 | **<0.001** | 744±118(a) | 293±35.35(b) | 120±25.59(c) | 75.79±11.16(c) |
| **Camphene** | 8.34 | 60.9 | **<0.001** | 14.64±2.78(a) | 5.56±1.03(a) | 2.07±0.62(b) | 1.07±0.22(b) |
| Verbenene | 8.51 | 3.44 | 0.088 | 0.77±0.29 | 0.62±0.24 | 0.37±0.18 | 0.19±0.07 |
| Sabinene | 9.50 | 0.67 | 0.46 | 0.73±0.3 | 0.13±0.07 | ND | ND |
| **β-Pinene** | 9.13 | 94.71 | **<0.001** | 1162±173(a) | 423±62.9(b) | 135±32.81(c) | 68.84±15.77(c) |
| **β-Myrcene** | 9.54 | 37.17 | **<0.001** | 24.6±6.54(a) | 11.2±1.83(ab) | 3.75±1.29(bc) | 1.88±0.89(c) |
| Unknown | 9.85 | 0.53 | 0.664 | 1.93±0.61 | 2.65±0.62 | 2.07±0.5 | 1.72±0.46 |
| **α-Phellandrene** | 9.88 | 16.52 | **0.002** | 1.6±0.4(a) | 0.74±0.19(ab) | 0.43±0.18(ab) | 0.2±0.05(b) |
| **α-Terpinene** | 10.21 | 12.61 | **0.005** | 0.4±0.169(a) | 0.16±0.04(ab) | 0.08±0.02(ab) | 0.03±0(b) |
| ***p*-Cymene** | 10.43 | 39.19 | **<0.001** | 26.89±5.3(a) | 11.6±2.53(ab) | 4.87±1.45(bc) | 3.04±0.52(c) |
| **Limonene** | 10.51 | 44.23 | **<0.001** | 51.15±10.26(a) | 21.07±3.56(a) | 7.58±2.15(b) | 4.79±0.94(b) |
| **β-Phellandrene** | 10.55 | 46.17 | **<0.001** | 152.±39.71(a) | 65.86±8.5(a) | 22.77±8.59(b) | 11.91±4.2(b) |
| γ-Terpinene | 11.37 | 0.86 | 0.422 | 1.36±0.36 | 0.67±0.01 | ND | ND |
| **α-Terpinolene** | 12.16 | 33.82 | **<0.001** | 1.05±0.23(a) | 0.73±0.12(a) | 0.21±0.06(b) | 0.16±0.04(b) |
| *p*-Cymenene | 12.19 | 0.37 | 0.551 | 0.59±0.15 | 0.57±0.11 | 0.57±0.13 | 0.88±0.36 |
| **Sum** |  | 35.36 | **<0.001** | 2177±384(a) | 835±123(b) | 297±81.3(c) | 168±36.1(c) |
| ***Oxygenated monoterpenes*** |  | | | | | | |
| **1,8-Cineole** | 10.61 | 47.05 | **<0.001** | 6.93±1.31(a) | 4.3±0.64(a) | 1.7±0.33(b) | 0.79±0.14(b) |
| Linalool oxide | 11.73 | 1.88 | 0.188 | 0.13±0.03 | 0.31±0.08 | 0.4±0.15 | 0.39±0.15 |
| **Fenchone** | 12.15 | 10.96 | **0.004** | 1.64±0.35(b) | 1.76±0.59(b) | 1.36±0.37(ab) | 2.26±1.07(a) |
| *trans*-4-Thujanol | 12.42 | - | - | ND | ND | ND | ND |
| ***exo*-Fenchol** | 12.82 | 33.56 | **<0.001** | 0.27±0.06(c) | 0.51±0.1(bc) | 0.68±0.08(ab) | 1.63±0.38(a) |
| **Thujone** | 12.93 | 6.79 | **0.022** | 0.24±0.08(a) | 0.12±0.05(a) | 0.06±0.02(a) | 0.04±0(a) |
| *p*-Isopropylcyclohexanol | 13.41 | 2.47 | 0.142 | ND | 0.15±0.03 | 0.19±0.06 | 0.33±0.1 |
| ***trans*-Pinocarveol** | 13.48 | 19.16 | **<0.001** | 0.41±0.11(b) | 0.85±0.22(b) | 1.29±0.34(ab) | 2.06±0.31(a) |
| **Camphor** | 13.63 | 12 | **0.003** | 10.28±1.51(b) | 14.12±1.78(ab) | 16.41±1.98(ab) | 30.9±8.3(a) |
| Camphene hydrate | 13.73 | 2.57 | 0.093 | 0.18±0.03 | 0.25±0.05 | 0.31±0.05 | 0.35±0.03 |
| Pinocamphone | 14.43 | 0.13 | 0.727 | 4.81±0.62 | 4.11±0.73 | 3±0.56 | 3.03±0.61 |
| **Pinocarvone** | 14.10 | 14.48 | **0.003** | 0.29±0.04(a) | 0.22±0.08(ab) | 0.05±0.01(ab) | 0.03±0.01(b) |
| *endo*-Borneol | 14.18 | 1.29 | 0.275 | 1.77±0.41 | 3.34±0.51 | 4.18±0.61 | 9.9±1.13 |
| 3-Thujene-2-one | 14.34 | - | - | ND | ND | ND | ND |
| Isopinocamphone | 14.40 | 1.84 | 0.193 | 3.08±0.95 | 4.14±1.56 | 4.57±1.32 | 6.33±2.07 |
| Terpinen-4-ol | 14.46 | 0 | 0.988 | 1.18±0.13 | 1.51±0.34 | 1.37±0.32 | 1.5±0.38 |
| ***p*-Cymene-8-ol** | 14.65 | 178.59 | **<0.001** | 0.36±0.05(d) | 0.8±0.06(c) | 1.33±0.12(b) | 1.97±0.09(a) |
| **α-Terpineol** | 14.79 | 9.08 | **0.008** | 2.97±0.43(b) | 3.77±0.11(ab) | 3.97±0.51(ab) | 5.09±0.6(a) |
| **Myrtenol** | 14.94 | 53.54 | **<0.001** | 0.32±0.08(c) | 2±0.49(b) | 4.44±0.99(ab) | 9.5±2.02(a) |
| Verbenone | 15.28 | 2.85 | 0.126 | 0.19±0.02 | 0.22±0.01 | 0.45±0.13 | 0.35±0.07 |
| 2-Hydroxycineole | 15.58 | 1.04 | 0.332 | ND | 0.09±0 | 0.1±0.03 | 0.15±0.05 |
| **Thymol methyl ether** | 15.85 | 18.59 | **<0.001** | 2.23±0.36(a) | 1.84±0.27(ab) | 1.03±0.25(bc) | 0.83±0.14(c) |
| Myrtanol isomer1 | 16.73 | - | - | ND | ND | TR | TR |
| Myrtanol isomer2 | 16.30 | - | - | TR | TR | TR | 0.1±0.03 |
| *p*-Menth-2-en-7-ol | 16.42 | - | - | ND | ND | ND | ND |
| Myrtanol isomer3 | 16.48 | 2 | 0.2 | ND | 0.05±0(a) | 0.14±0.03(ab) | 0.28±0.11(b) |
| **Myrtenyl acetate isomer1** | 17.43 | 5.73 | **0.048** | 1.15±0.49 | 0.34±0.01 | 0.1±0.02 | ND |
| Myrtenyl acetate isomer2 | 18.13 | - | - | ND | ND | ND | ND |
| **Sum** |  | 3.53 | **0.04** | 38.18±3.77(c) | 44.45±4.7(bc) | 46.84±6.69(bc) | 77.67±16.76(a) |
| ***Sesquiterpenes*** |  | | | | | | |
| **α-Longipinene** | 18.63 | 49.99 | **<0.001** | 0.46±0.11(a) | 0.22±0.06(a) | 0.08±0.03(b) | 0.04±0(b) |
| **Longicyclene** | 19.10 | 40.08 | **<0.001** | 0.52±0.12(a) | 0.28±0.07(a) | 0.09±0.04(b) | 0.05±0(b) |
| Longifolene | 19.88 | 17.97 | 0.001 | 3.23±0.92(a) | 2.23±0.42(ab) | 1.1±0.36(bc) | 0.8±0.19(c) |
| **(*E*)-β-Caryophyllene** | 20.17 | 30.57 | **<0.001** | 10.3±2.52(a) | 6.87±1.39(ab) | 2.95±0.85(bc) | 1.94±0.46(c) |
| (*E*)-β-Caryophyllene (fungus) | 20.56 | - | - | ND | ND | ND | ND |
| (*E*)-β-Farnesene | 20.84 | 3.44 | 0.106 | 0.58±0.16 | 0.48±0.05 | 0.16±0.05 | ND |
| **Humulene** | 20.90 | 18.26 | **0.001** | 3.94±0.86(a) | 2.23±0.41(ab) | 1.38±0.48(b) | 1.02±0.26(b) |
| Caryophyllene oxide | 23.56 | - | - | ND | ND | ND | ND |
| **Sum** |  | 9.12 | **0.001** | 18.91±5.21(a) | 12.06±2.75(ab) | 5.67±2.02(bc) | 3.86±1.02(c) |

^#^- Estimated retention time from GC-MS

***^$^-***Significant differences between time points are denoted by small letters (ANOVA, followed by Tukey’s test, *P<0.05*
