# Supplementary material for: Conifer-killing bark beetles locate fungal symbionts by detecting volatile fungal metabolites of host tree resin monoterpenes
Source: PLoS Biol. 2023 Feb 21;21(2):e3001887. doi: 10.1371/journal.pbio.3001887 (PMC9943021; doi:10.1371/journal.pbio.3001887)
Supplement: S8 Table — $The purity of each compound was calculated from GC–MS analysis. (DOCX) [file pbio.3001887.s023.docx]

| **Compounds** | **Purity^$^** | | **CAS number** | | **Composition (V/V %)** | |
| --- | --- | --- | --- | --- | --- | --- |
| Tricyclene | >99 | 508-32-7 | | 0.33 | |  |
| α-Thujene | 80 | 2867-05-2 | | 0.27 | |  |
| (-)-α-Pinene | >99 | 80-56-8 | | 24.35 | |  |
| (+)-α-Pinene | >99 | 80-56-8 | | 28.86 | |  |
| (-)-Camphene | >99 | 79-92-5 | | 0.98 | |  |
| (+)-Camphene | >99 | 79-92-5 | | 0.33 | |  |
| (-)-Sabinene | 76 | 3387-41-5 | | 0.38 | |  |
| (+)-Sabinene | 76 | 3387-41-5 | | 1.85 | |  |
| (-)-β-Pinene | >99 | 127-91-3 | | 34.18 | |  |
| Myrcene | 93 | 123-35-3 | | 2.93 | |  |
| α-Phellandrene | >99 | 99-83-2 | | 0.16 | |  |
| *delta*-3-Carene | >99 | 13466-78-9 | | 1.47 | |  |
| α-Terpinene | 92 | 99-86-5 | | 0.16 | |  |
| *p*-Cymene | >99 | 99-87-6 | | 0.33 | |  |
| (-)-Limonene | >99 | 5989-27 | | 0.82 | |  |
| (+)-Limonene | >99 | 5989-27 | | 0.82 | |  |
| 1,8-Cineole | >99 | 470-82-6 | | 0.16 | |  |
| γ-Terpinene | 97 | 99-86-5 | | 0.33 | |  |
| Terpinolene | >99 | 586-62-9 | | 0.65 | |  |
| (-)-Bornyl acetate | >99 | 76-49-3 | | 0.65 | |  |

***Table S8*:** Composition of synthetic monoterpene mixture used in bioassays. ^$^ The purity of each compound was calculated from GC-MS analysis.
