# Supplementary material for: Conifer-killing bark beetles locate fungal symbionts by detecting volatile fungal metabolites of host tree resin monoterpenes
Source: PLoS Biol. 2023 Feb 21;21(2):e3001887. doi: 10.1371/journal.pbio.3001887 (PMC9943021; doi:10.1371/journal.pbio.3001887)
Supplement: S9 Table — Wash, supernatant from beetles immersed in 0.05% Triton X in 500 μL PBS buffer (pH 7.4); lysate, crushed beetles in 500 μL PBS buffer (pH 7.4); NP, not present. The data underlying this Table can be found at https://doi.org/10.6084/m9.figshare.21692156.v1. (DOCX) [file pbio.3001887.s024.docx]

|  |  | Colony forming units (CFUs)/mL | | | | | |
| --- | --- | --- | --- | --- | --- | --- | --- |
| Beetle treatment | Sample type | Potato dextrose agar | | | Luria agar | | |
|  |  | *Bacteria* | *Yeast* | *Ophiostomatoid fungi* | *Bacteria* | *Yeast* | *Ophiosotmatoid fungi* |
| Unaltered | Wash | NP | 6083 ± 2655 | NP | NP | 6166 ± 2565 | NP |
|  | Lysate | 1167 ± 385 | 500 ± 236 | 250 ± 156 | 2167 ± 481 | 917 ± 478 | 83 ± 76 |
| Fungus-free (FF) | Wash | NP | NP | NP | NP | NP | NP |
|  | Lysate | >10^5 | 2100 ± 1565 | NP | >10^5 | NP | NP |
| *G. penicillata*-reinoculated in FF | Wash | 900 ± 415 | NP | 340 ± 154 | 900 ± 698 | NP | 300 ± 179 |
|  | Lysate | 62000 ± 33367 | NP | 100 ± 75 | 39000 ± 26015 | NP | 100 ± 89 |

***Table S9*:** Average colony forming units (CFUs/mL) from untreated, fungus-free, and fungus-free *G. penicillata*-reinoculated *I. typographus* bark beetles (*n* = 5 or 6 beetles). Wash, supernatant from beetles immersed in 0.05 % Triton X in 500 µL PBS buffer pH 7.4; lysate, crushed beetles in 500 µL PBS buffer pH 7.4; NP, not present The data underlying this Table can be found at https://doi.org/10.6084/m9.figshare.21692156.v1
