# Supplementary material for: Conifer-killing bark beetles locate fungal symbionts by detecting volatile fungal metabolites of host tree resin monoterpenes
Source: PLoS Biol. 2023 Feb 21;21(2):e3001887. doi: 10.1371/journal.pbio.3001887 (PMC9943021; doi:10.1371/journal.pbio.3001887)
Supplement: S10 Table — Approximately 300 mg of bark samples were dissolved in 1 mL PBS buffer solution, and dilutions were plated on PDA. NP, not present. $Only one gallery sample was tested due to low sample availability. (DOCX) [file pbio.3001887.s025.docx]

| Treatments | Colony forming units (CFUs)/mL | | | |
| --- | --- | --- | --- | --- |
|  | *Bacteria* | *Yeasts* | *Ophiostomatoid fungi* | *Molds/other fungi* |
| Fungus-free | >10^6 | NP | NP | NP |
| Fungus-free | >10^6 | 20 | NP | 2000 (red mold), 60 (dark green mold) |
| Fungus-free | >10^6 | 30 | NP | 40 (dark green mold) |
| Fungus-free | 70 | NP | NP | NP |
| Fungus-free | NP | 210 | NP | 140 (dark green mold), 3000 (red mold) |
| Unaltered | NP | 27000 | 70 | NP |
| Unaltered | 9000 | 55000 | 10 | NP |
| Unaltered | 21000 | 3000 | 40 | NP |
| Unaltered | NP | NP | 10 | 1200 (red mold) |
| Unaltered | NP | 21000 | 20 | NP |
| Unaltered | NP | 13000 | 10 | NP |
| Unaltered | >10^6 | 272000 | NP | NP |
| *G. penicillata*-reinoculated FF^$^ | NP | NP | 100 | NP |

***Table S10*:** Colony forming units (CFUs/mL) obtained from bark beetle gallery samples infested by fungus-free beetles, and fungus-free beetles reinoculated with *G. penicillata*, and untreated control beetles. Approximately 300 mg of bark samples were dissolved in 1 mL PBS buffer solution and dilutions were plated on PDA. NP, not present;

^$^ Only one gallery sample was tested due to low sample availability.
