# Supplementary material for: Conifer-killing bark beetles locate fungal symbionts by detecting volatile fungal metabolites of host tree resin monoterpenes
Source: PLoS Biol. 2023 Feb 21;21(2):e3001887. doi: 10.1371/journal.pbio.3001887 (PMC9943021; doi:10.1371/journal.pbio.3001887)
Supplement: S2 Method — (DOCX) [file pbio.3001887.s027.docx]

**2. Analysis of *G. penicillata* and *Trichoderma sp.* headspace volatiles**

Headspace volatiles from *G. penicillata* and *Trichoderma sp.* grown on PDA amended with terpene mix were collected using SPME fiber (coating-50/30 µm DVB/CAR/PDMS; core/assembly type-StableFlex/ stainless steel (1 cm), Supelco). Briefly, 1 ml of PDA containing 0.1 mg/ml of mix of terpenes was poured in 15 ml glass test tubes and allowed to solidify before *G. penicillata* and *Trichoderma* sp. were inoculated. Control tubes contained fungus-free PDA plus terpenes. After 5 d post inoculation, headspace volatiles were collected for 15 mins from each test tube using SPME fiber and were manually injected immediately in the GC inlet. The fibers were conditioned prior to headspace collection and cleaned in between each collection following the manufacturer’s protocol. GC-MS analysis was carried out in Agilent 7890A GC system coupled with Agilent 5975C mass detector. Injection program was configured in splitless mode at 250°C. Non-polar HP-5MS GC column (30m *250 µm* 0.25 µm) was used to separate compounds and helium was used as a carrier gas. The oven temperature started at 45°C and held for 1 min and then increased to 250°C at the rate of 10°C min^-1^ and finally held for 5 min. Compounds were identified by comparing their mass spectra with NIST reference library and later by matching their retention time and mass spectra to those of authentic standards.
