# Supplementary material for: Conifer-killing bark beetles locate fungal symbionts by detecting volatile fungal metabolites of host tree resin monoterpenes
Source: PLoS Biol. 2023 Feb 21;21(2):e3001887. doi: 10.1371/journal.pbio.3001887 (PMC9943021; doi:10.1371/journal.pbio.3001887)
Supplement: S3 Method — (DOCX) [file pbio.3001887.s028.docx]

**3. Analysis of beetle metabolites of host tree monoterpenes**

For identification of terpene metabolites produced by adult bark beetles, the beetle fumigation experiment was carried out using a synthetic terpene mixture in 20 ml scintillation vials. Briefly, two males were placed in clean 20 ml glass vials and 2 µl of terpene mixture was added to a clean 23 mm circular filter paper (Whatmann no. 3) that was secured tightly under the screw cap. Beetles were not in the direct contact with terpenes, but they were exposed to terpene vapors. Control treatments were dead beetles (freeze-killed) exposed to terpene vapors and live beetles without terpene vapors in glass vials. Each treatment was replicated 8 times. Terpene metabolites from beetles were extracted as follows: four beetles from each treatment were placed in 4 ml glass vials containing 1 ml of heptane and crushed with a clean plastic pestle. Then the slurry was centrifuged at 4000 rpm for 5 min and supernatant was transferred to a clean 1.5 ml glass vial. Since the beetle extract contained a high concentration of fatty acids, the extract was fractionated to separate fatty acids from polar compounds. A glass capillary was packed with 400 mg of Florosil^®^ (Sigma-Aldrich) and conditioned with 2 ml heptane. The beetle extract was concentrated to 100 µL under N_2_ air and added to the column. Then the vial that contained the beetle extract was rinsed twice with 100 µL to remove the left-over extract and added to the same column. The column was first washed with 2 mL heptane to remove all the hydrocarbons. The second wash was performed with 2 ml heptane + 20% acetone to collect all oxygenated derivatives that include oxygenated terpenoids. The final wash was done with 2 mL heptane + 50 % acetone to remove left-over polar compounds and conditioned again with 2 ml heptane before the next beetle extract was loaded for fractionation. Each fraction was analyzed in GC-MS to identify terpenoids produced by beetles and the concentration in each fraction was quantified using external calibration curves. The GC program was the same as described for headspace fungal volatiles and the injection volume was set at 2 µL.
