# Supplementary material for: Conifer-killing bark beetles locate fungal symbionts by detecting volatile fungal metabolites of host tree resin monoterpenes
Source: PLoS Biol. 2023 Feb 21;21(2):e3001887. doi: 10.1371/journal.pbio.3001887 (PMC9943021; doi:10.1371/journal.pbio.3001887)
Supplement: Attachment — Submitted filename: Response to reviewers.docx [file pbio.3001887.s029.docx]

Dear Dr Gershenzon,

Thank you for submitting your manuscript entitled "Bark beetles locate fungal symbionts by detecting volatile fungal metabolites of host tree resin monoterpenes" for review as a Research Article by PLOS Biology. As with all papers reviewed by the journal, yours was assessed and discussed by the PLOS Biology editors, by an academic editor with relevant expertise and in this case by three independent reviewers.

Based on the reviews, I regret that we will not be able to accept this manuscript for publication in the journal. I hope that you will find the reviewers' feedback helpful as you consider how to proceed with this work.

The rationale behind this decision is that while all of the reviewers think that the research question is of interest, they also raise some serious concerns. Specifically, you'll see that reviewer #2 is critical of the paper, recommending that we reject it outright; his/her main issue seems to be that the oxygenated monoterpenes analysed here could be generated by multiple sources, including the insects themselves, and that you present no evidence that fungi are a major contributor. Indeed, reviewer #3 two major points are somewhat related to this (that the timing is wrong for fungi, and that other players could produce these chemicals).

The Academic Editor agrees with these concerns, saying "I specifically agree that although reviewer #3 is more complimentary than reviewer #2, the issue of which fungi are doing what and the ultimate source of the compounds should be identified, and there is currently some ambiguity (based on timing / the fact that beetles could be the source of some of the compounds)," and stressing that this issue would need to be addressed with additional data. Thus we think that your study would require extensive additional work to achieve the level needed for PLOS Biology.

At this point, we can't know whether new data would support your conclusions, and as producing the data would require significant effort, we cannot in good faith invite resubmission. However, if you are willing to take on the extra work involved and comprehensively address the reviewers' concerns - and I must emphasize that this would be your choice - we would be willing to consider an extensively revised version as a new submission. As such, the article would receive a new number and submission date and we would evaluate it against any work published in the interim that might undermine the novelty. Although we would try our best to engage the same academic editor and reviewers, please be aware that this isn't always possible. Also, if the new experiments and data introduce new areas that require review we might need to consult additional/new reviewers. The new data would all need to be evaluated as if a first submission and further revisions might prove necessary.

We appreciate that the scale of the requested additional work is significant and you may well prefer to pursue publication of this work elsewhere. At this point, your manuscript is no longer under active consideration at PLOS Biology.

Please do feel free to contact me if you would like to discuss next steps for your manuscript. We might also be able to facilitate a faster consideration at one of the other PLOS journals; if this is of interest to you, please let me know.

If you do make the choice to revise and re-submit to PLOS Biology, you should provide a point-by-point response to the reviewers' comments when re-submitting. Please include this as a separate file, labelled 'Response to Reviewers'. Please also provide the tracking number of this manuscript [PBIOLOGY-D-21-01909R1] in the 'previous interaction' section of the online submission form and in your cover letter when re-submitting.

Sincerely,

Roli Roberts

Roland Roberts
Senior Editor
PLOS Biology
rroberts@plos.org

*Dear Dr. Roberts,*

*Thank you very much for giving us the opportunity to resubmit our revised manuscript titled “Bark beetles locate fungal symbionts by detecting volatile fungal metabolites of host tree resin monoterpenes”* [PBIOLOGY-D-21-01909R1] *for review as a research article in PLoS Biology. We thank the reviewers and the editors for the critical reading of our manuscript and for providing insightful comments. We have now performed additional chemical analysis and insect behavioral assays that we believe address the major concerns of the reviewers and strengthen the manuscript. Some of the highlights from our revision include:*

*1. We compared the oxygenated monoterpene profile of bark galleries constructed by fungus-free beetles, fungus-free beetles reinoculated with a symbiotic fungus, untreated control beetles and untreated bark without beetles. These results showed that removal of symbiotic fungi significantly reduced the concentrations of the oxygenated monoterpenes that are behaviorally important to beetles and strengthen our original findings that symbiotic fungi are the main source of these compounds emitted from beetle galleries.*

*2. Comparison of the volatile profiles of a symbiotic versus a saprophytic fungus growing in the presence of a similar host terpene mixture showed that the profile of the symbiotic fungus is distinct from that of the saprophyte, both in terms of volatile composition and quantity. While adult beetles were attracted to the volatiles of the symbiotic fungus, they exhibited an indifferent response to the volatile profile of the saprophytic fungus, showing that volatiles are specifically exploited to find symbionts.*

*3. Analysis of volatiles produced by male beetles when fumigated with vapors of a similar host terpene mixture used in 2 showed that oxygenated monoterpenes produced by beetles are different from those produced by fungi.*

*4. We expanded of our discussion on the role of fungal volatile emission in the bark beetle life cycle by assessing its significance to beetles in different population phases. Also, we synthesized the results of the current study with previous studies on microbial production of insect semiochemicals.*

*We also noted reviewer 2’s suggestion that a major source of oxygenated monoterpenes might be the spruce tree itself. The reviewer believes that bark beetle host trees could produce these volatiles but only in response to fungal growth and beetle damage in the bark. Thus, they appear to be fungal or insect metabolites, but could arise from the tree. Although we managed to successfully remove fungi from beetles, the logs in which the beetles were infested contained too many saprophytes and endophytes to allow a rigorous test of this hypothesis. One way to determine whether the oxygenated monoterpenes are produced by the tree is to search for tree genes and enzymes that produce these compounds in response to fungal infection. We plan to carry out these investigations, but they are quite extensive and beyond the scope of the present manuscript.*

*The text in the revised manuscript and response letter is marked in orange and purple respectively.*

*Thank you again for your consideration of our revised manuscript. Please feel free to contact us if you have any questions.*

*Best regards,*

*Jonathan Gershenzon and Dineshkumar Kandasamy*

REVIEWERS' COMMENTS:

Reviewer #1:

The authors present a detailed study of the fungal symbiont G. penicillate, bark beetles, and their host plant. In the study, the authors provide results from numerous behavioral bioassays using bark beetles and the identified compounds from G. penicillate inoculated agar treatments among others.

Overall, the paper is very interesting and well-researched; however, there are some items that detract from its overall impact and readability.

- the abstract is poorly worded. Please revise for clarity, direct wording, and follow the format of problem, impact of problem, what the research studied, results of research, and impact of results

We thank the reviewer for pointing out deficiencies in the abstract. The format and wording of the abstract have now been extensively revised, and we hope that this has improved its clarity.

- please delineate the differences of this report and two recent and related publications authored by some of the present manuscript's authors
o Tanin, Kandasamy, and Krokene. Frontiers Microbiol, 2021, 12:695167 (cited)
o Zhao, Axelsson, Krokene, Karlson. J Chem Ecol, 2015, 41:848-852 (not cited)

The paper of Tanin et al. (2021) investigated the response of *I. typographus* to fungi vectored by other species of bark beetles, not the native ones studied in this work. The study of Zhao et al. (2015) reported about the fact that *I. typographus* fungal symbionts can sometimes produce the bark beetle aggregation pheromone, 2-methyl -3-buten-2-ol, whereas fungal monoterpene oxidation products are the focus of this work. We now cite both papers in our discussion (lines 391-395 and lines 432-434) to call attention to these results in relation to the present work.

- the experimental is light on some important procedures

o were control blank runs (same set up sans treatment) to subtract profiles from treatments (e.g., solvent-, medium-, container volatiles)?

Yes, appropriate controls were run during each experiment to identify the volatile peaks arising from the containers, solvent and uninfected medium that were trapped on both solid adsorbent and PDMS tubes. These were then excluded from the analysis. This information has now been added to the Methods section in lines 657-658

o RI values and comparison to literature?

We did not calculate formal RI values in identifying compounds, but instead compared their raw retention times as well as mass spectral data to those of authentic standards and those in reference libraries. This was done for the analyses of both adsorbent-trapped and PDMS tube-trapped volatiles.

o the same two bullets for the PDMS tubes

Please see the answers above

- for the synthesized compounds
o were the NMR results compared to any known literature?
o is this the first report for these compounds? If so, they would require significantly more physical and spectroscopic information
o include 1D and 2D in supplemental?

Both of these compounds have been previously reported in the literature, and the NMR results were compared to the published data. We have now included the NMR data for both compounds in supplemental figures (Figs. S13 and S14) since the 2D-NMR data have not been reported before. We also cited the previous reports in the Methods section (lines 715 and 736).

- in the bioassays were 50/50 control experiments performed (two controls to ensure 50% to each side)?

Yes, this experiment was done and the choice was indeed found to be around 50 % for each side. This result has now been added to the Methods section in lines 773-775.

- L719: what about male-female differences in the pheromone mixture experiments (fig 5)

We thank the reviewer for bringing this to our attention. We now explain why we tested males and females separately in the Methods section (lines 798-800) and report on the male-female differences in the Results section (lines 303-305) and elaborate in the Discussion section (lines 409-424).

Other minor items:
- perhaps place the genus of bark beetle early on

This has now been moved to the first sentence of the abstract.

- L22: how overcome tree defenses

We have briefly mentioned that beetles overcome tree defenses by mass attacks (line 25).

- L39: vague statement. What does "tunneling on diets" mean?

We have now rephrased this statement.

- L41: what kind of cues? Diet? Attraction? Increased consumption? Inefficient sentence. Perhaps state it more directly and clearly

The text has now been revised to indicate these are attraction cues.

- the manuscript seems overlong and could use some trimming. Perhaps re-visit the manuscript and make it more concise in all sections. In particular, the material and methods section could be toned down the introductory/non-results statements at the beginning of each sub-section

We have now shortened the manuscript in places, in particular by removing introductory statements in the results and methods sections.

- L63: redundant sentence. Airborne chemical cues are volatile organic compounds

The text has now been changed.

- L66: define insect success

The text has now been changed

- L79: please reword. The first part of the sentence says bark beetles kill spruce trees due to global warming and management practices. Move the last part of the sentence forward

The text has now been moved to lines 77-79 to make the point more clearly.

- L109: don't need numbers

These have now been removed

- L135: 4 d is a long interval post-inoculation. Were headspace volatiles collected soon after inoculation to see if there were any differences? Some microbes start producing within hours of inoculation

We did not measure headspace volatiles in fungus-infected bark at shorter time intervals due to difficulties in consistently detecting the low concentrations present. However, previous studies have detected fungal volatiles within 1 d post inoculation (Zhao et al., 2015 and 2019), and we now cite this work in the Discussion section (lines 431-434).

- L185: except?

We have now corrected this word.

- general - what is a free-living fungal symbiont. Perhaps describe for a broad audience

We have now explained this in more detail to make it clearer and in part replaced “free-living symbiont” with “ectosymbiont”.

Reviewer #2:

Although the manuscript PBIOLOGY-D-21-01909R1 addresses an important topic and provides some interesting and publishable data on insect-fungal symbioses, the work is not of sufficient novelty, completeness, or general interest to merit publication in Plos Biology.

The paper fails to mention that many of the fungi that bark beetles inoculate into galleries when they attack are not beneficial, but in fact are deleterious (this is particularly true of the blue-staining fungi) and can prevent brood development. Most fungi transported by bark beetles are phoronts (some of which are transported by mites phoretic on the beetles), and the mere fact that they are introduced through beetle mining is no indication of whether the fungi are mutualistic or not. Typically, the deleterious fungi grow more rapidly in the host than the beneficials, hence it would seem far more likely that fungal odors encountered by beetles would be repellant than attractive. These considerations are very important to development of the authors' hypothesis (and are some of the most basic aspects of bark beetle-fungus associations) but are not mentioned. For their thesis to be plausible, the authors would need to show that beneficial fungi produce a different blend of volatile compounds when in contact with host terpenes than do the deleterious species.

We thank the reviewer for bringing up this comment on the role of blue stain fungi to bark beetles and the fact that many may not be beneficial. We see the situation as follows:

1. There are multiple lines of evidence to show that blue-stain fungi are beneficial to their vectors in various bark beetle species. A well-known example is the blue-stain fungus *G. clavigera*, associated with the mountain pine beetle, *D. ponderosae*, which is a nutritional mutualist of this beetle (*Six and Paine 1998*).
2. There is at this point only a single fungus, *Ophiostoma minus*, a fungal associate of southern pine beetle *Dendroctonus frontalis*, that is reported to be deleterious to larval development. In fact, a recent study showed that *O. minus* may actually be beneficial to a secondary bark beetle, *Hylurgops reticulatus,* suggesting that the nature of association between blue-stain fungi and the bark beetle is context specific (*Six DL & James JE 2020*).
3. In our study system, while the symbiotic fungal associates of *I. typographus* have long been suggested to provide multiple benefits to beetles (Solheim 1992), there has not been much research to prove this using more modern tools, and we consider the question to be still open.

We appreciate the reviewer’s suggestion to check if beneficial fungi produce different blends of volatiles than deleterious species when in contact with host tree terpenes. Thus, we looked for a non-beneficial fungal species in our system to test and noted a saprophytic *Trichoderma sp.* in our galleries that was present when the quality of the bark was poor and was detrimental to the performance of developing larvae (unpublished data). In comparing this *Trichoderma sp.* to *G. penicillata*, the symbiotic fungus we used elsewhere in the manuscript, we found different blends of oxygenated monoterpenes to be produced when in contact with spruce monoterpenes (Fig. S9). Moreover, in bioassays, only the symbiotic fungus was attractive to beetles whereas adult beetles were unresponsive or slightly repelled by the saprophyte (Fig. S1). These new results are now mentioned in the text on lines 181-185 and on line 125. They show (at least for this two species comparison) that beneficial fungi convert host tree terpenes to a different blend of volatile compounds than a saprophyte, and that beetles are able to distinguish between the blends and seek out the beneficial fungus.

Although they acknowledge it, the authors seem to ignore the significance of the fact that oxygenated monoterpenes have many different sources within the environment of a bark beetle invading a host. Bark beetles themselves produce most of the oxygenated compounds identified in this study as "fungal metabolites" when exposed to host-produced hydrocarbon monoterpenes.

We were pleased that the reviewer commented on our discussion about the existence of multiple potential sources of oxygenated monoterpenes in this bark beetle-fungus-host tree system. However, we were not sure about the reviewer’s claim that “bark beetles produce most of the oxygenated compounds identified in this study as fungal metabolites”. *Ips typographus* bark beetles have indeed been shown to make oxygenated monoterpenes when in contact with host tree monoterpenes, but the major compounds reported to be made, *cis-* and *trans*-verbenol, verbenone, myrtanal, and myrtenol (*Birgersson et al. 1983; Ramakrishnan et al. 2022*), do not overlap with the major oxygenated monoterpenes we identified to be formed from host tree monoterpenes by fungal symbionts. To ensure that our fungal metabolites had not been simply missed in earlier studies on the beetles, we incubated male *I. typographus* beetles with vapor from a synthetic mixture of host tree (*Picea abies*) monoterpenes. Following the incubation, we extracted terpenes from the whole insect and analyzed them using GC-MS to identify the terpene metabolites produced by beetles. Our results generally agree with the existing literature on the terpene metabolites produced by beetles and showed that only a very few compounds, such as verbenone and borneol, are actually common products of both beetle and fungal metabolism of host tree terpenes. (And, since these were found primarily in extracts of dead beetles, and we also detected them in media without fungi and beetles, they are likely auto-oxidation products.) The major compounds present in the extracts of live beetles were not produced at all by the fungi we studied. Thus, we conclude that fungi and not beetles are indeed responsible for the monoterpene oxidation products identified in this study. Results from this additional experiment are included in Fig. S12 (and below) and are discussed in the text on lines 539-542.

***Figure:*** *Concentration of oxygenated monoterpenes derived from live and dead males following incubation with a host-derived terpene mixture; (a) trans-verbenol, (b) cis-verbenol, (c) borneol, (d) myrtanal, (e) myrtenol, (f) verbenone, (g) trans-myrtanol, (h) perillaldehyde, (i) nopinone, and (j) pinocarvone produced by live and dead male I. typographus fumigated with spruce monoterpene mix.*

Oxygenated monoterpenes are detoxification products of hydrocarbon monoterpenes and are produced by a wide variety of organisms through the action of cytochrome P450 enzymes. Hence, a behavioral response to these compounds could reflect an ecological interaction with many possible organisms in the environment. Because of the multiple possible natural sources, there is no basis for arguing that beetle attraction to these oxygenated monoterpenes is an adaptive response for locating fungal growth.

The reviewer is certainly correct that many different organisms in the bark beetle gallery could be metabolizing monoterpenes, perhaps many by P450s, and the spectra of products could be similar. However, this is not what our data show. The profiles of monoterpene oxidation products are distinct among beetles, symbiotic fungi, and a saprophytic fungus. We agree with the reviewer that a single oxygenated monoterpene could have multiple sources, but the blend of oxygenated monoterpenes produced by each species of fungus and *I. typographus* beetles seems chemically unique. Furthermore, our bioassays showed that the volatile profile can be recognized and distinguished by bark beetles and that only the volatile mixture of symbiotic fungi was attractive to beetles. We have now expanded our discussion section in lines 384-388 to amplify this point.

Furthermore, the possibility that compounds produced from spruce bark inoculated with fungi might result from a generalized defensive response of the host tissue is not considered. That is, it is possible that these same oxygenated monoterpenes would be generated from beetle-attacked host tissue in the absence of the fungi. Although mechanically damaged host tissue (the excised bark) may not produce the compounds, chemical or physical elicitors from the beetles could cause defensive reactions in the tissue that may result in the very same compounds.

We thank the reviewer for pointing out that oxygenated monoterpenes may originate with the host tree, in particular as a defense response to bark beetles or fungi. We think this an entirely plausible notion and have discussed this possibility in lines 498-510. But, it is challenging to test this hypothesis. To learn more about the biochemical capabilities of the tree during bark beetle attack in the absence of the symbiotic fungus, we reared beetles without fungus using a semi-artificial diet. However, when these fungus-free beetles were allowed to colonize fresh spruce logs, their galleries contained no symbionts, but a plethora of saprophytes or endophytes. Hence, the oxygenated monoterpenes identified in these samples, as shown in Fig. 3, very likely originated from these fungal contaminants (S10 table) and not from the response of the tree to beetle tunneling. We also tried to sterilize logs by heat treatment, but this resulted in loss or oxidation of host tree terpenes and beetles rejected these heat-treated logs. Therefore, we think that the only feasible option to answer this question is to search for the biosynthetic machinery at the genetic level that could be involved in the production of oxygenated monoterpenes in phloem tissues in response to fungi, We hope to do this in the future, but the work is clearly beyond the scope of the present manuscript. Interestingly, a recent study showed that treating bark of healthy *P. abies* trees with methyl jasmonate (no fungi involved) resulted in a significant increase in the production of monoterpenes and oxygenated monoterpene such as 1,8-cineole (*Schiebe et al., 2019*). However, these authors did not detect any of the oxygenated monoterpenes identified in this work as fungal metabolites, suggesting that whatever oxygenated monoterpenes are made by the host tree, the profile does not significantly overlap with those produced by symbiotic fungi. We have now added some of these comments to the discussion in lines 515 and the following.

Evidence that fungi might be one agent contributing to the blend of behaviorally-active, oxygenated monoterpenes associated with bark beetle attacks has been published numerous times before. Hence the hypothesis proposed here is not novel or original, or - as far as I can tell - worthy of renewed or increased interest based on the authors' findings.

We were somewhat surprised to read the reviewer’s assertion that evidence for fungal contribution to the blend of oxygenated monoterpenes associated with bark beetle attack had been published before. We searched the literature again, but could find only one paper on the topic, *Leufvén et al., 1987* (cited already in the manuscript), which showed that yeasts associated with *I. typographus* produced some of oxygenated monoterpenes such as terpinen-4-ol, and α-terpineol reported in this study. However, the precursors for these metabolites were not identified. Additionally, none of the fungus-produced oxygenated monoterpenes identified in this study or by *Leufvén et al* were shown to be behaviorally active in earlier studies on *I. typographus* except for verbenone. Thus, we conclude that ours is indeed the first study to show that symbiotic ophiostomatoid fungi are the main source of *I. typographus*-attractive oxygenated monoterpenes in *Picea abies* bark.

Something lacking from all such studies, including this one, is evidence from controlled experiments that fungi are contributing significantly to the quantities of oxygenated monoterpenes arising from beetle attacks on natural host substrate. That is, Koch's postulates have yet to be fulfilled for the proposed interaction. This would require comparisons of odors from attacks on host tissue by unaltered beetles, axenically reared beetles, and axenically reared beetles with fungal propagules added artificially.

We thank the reviewer for setting a clear standard of proof to demonstrate the contribution of symbiotic fungi to oxygenated monoterpenes upon bark beetle attack. As explained above, we tried to reach this standard after reading these comments. We experimentally manipulated beetles to remove the fungi and reinoculated fungus-free beetles with a specific fungus (*G. penicillata*) in their natural substrate, comparing the volatile profile of bark samples colonized by these beetles with that of untreated beetles. This new experiment shows that the removal of blue stain fungi resulted in significant reduction in important oxygenated compounds, providing convincing evidence that ophiostomatoid fungi are the main source of oxygenated monoterpenes. Additionally, we found that the composition and quantity of volatiles varied depending on the identity of the microbial community and its size. Among fungi, symbionts produce higher amounts of oxygenated monoterpenes, when compared to saprophytes and fungus-free bark. These results are presented in Fig. 3 and in lines 189-222.

L115-130, Fig. 1 This set of experiments alone provides no evidence to support the hypothesis that the fungus is producing novel, attractive volatile compounds when in the presence of host substrate (Statement L124-126). An essential control experiment is missing. The authors did not test whether spruce bark agar alone was more attractive than PDA, and the results of Fig. D could be explained by a greater attractiveness of SBA over PDA. Hence the data are irrelevant.

We have now performed the missing “essential control” suggested by the reviewer. When we carried out the choice assay with beetles between empty PDA and SBA, we found a strong attraction to SBA as expected since SBA is the host substrate for this beetle. We agree with the reviewer that Fig. 1D could indeed be explained by the greater attractiveness of SBS vs. PDA, which we have now confirmed. However, given that SBA+fungus is more attractive than SBA alone (Fig. 1C), it seems more reasonable to conclude that the fungus is producing attractive compounds, and is releasing attractive volatiles to a greater extent on SBA than PDA (Fig. 1D).

L301-331 This set of experiments is not relevant to the hypothesis that the fungus is producing novel, attractive volatile compounds when in the presence of host substrate (Statement L328-331). They demonstrate that fungal growth increases mining into media but fail to show that there is a statistically significant interaction between presence of the fungus and the presence of any of the three tested monoterpenes. The statistical model apparently did not include this interaction (Table 1). Furthermore, compounds stimulating tunneling could be non-volatile, so the experiment cannot support or refute the core thesis of the paper.

We completely agree with the reviewer’s interpretation about what stimulates beetle tunneling into the diet. Using multiple logistic regression, we showed that the presence of the fungus increases tunneling probability into the diet. Yet, we were unable to generate a significant model by considering the interaction between the presence of fungus and the presence of any of the three tested monoterpenes shown in Table 1. We also failed to see a significant effect on the interaction between the fungus and monoterpenes in a multiple regression analysis. However, we could clearly show that the presence of the fungus in a beta-pinene enriched diet resulted in significantly longer tunnel length (Fig. 6B). Thus, the interaction of beta-pinene and the fungus had a positive effect on tunneling, but the presence of the fungus increases tunneling probability on its own. We thus fully agree with the reviewer that both volatile and non-volatile cues could stimulate the tunneling behavior of beetles.

L131-171 As I discussed earlier, odors produced by live bark colonized by fungi could be a response by the plant tissue to invasion rather than from metabolism by the fungi.

We agree with the reviewer that this is a plausible hypothesis, but as previously mentioned proving it is difficult. We can readily show that the fungus produces oxygenated monoterpenes from some pure monoterpene hydrocarbons without the tree, but it might be impossible to rule this out unequivocally for the tree as well. If the tree would require fungal presence for stimulating oxygenated monoterpene biosynthesis, it might be impossible to decide which organism was actually responsible without identifying the specific genes (from the plant or fungal genome). This is clearly a long-term project, though we have already embarked on it. One argument for the fungus versus the tree as a source of oxygenated monoterpenes is our comparison of the volatile profiles of various fungal species upon *P. abies* infection (Figs. S2, S3 S9). We found species-specific VOC profiles for the different fungi when they colonized spruce bark. These seem more likely to occur if the compounds were made by the fungus. If these compounds were produced by the tree as a defense response, one might expect a very similar response to each of these ophiostomatoid fungi. We have now added some of these points to the discussion (lines 515 and following).

The authors indicate that oxygenated monoterpenes "reach(ed) a maximum" at 18 d, however, this was the last date of their sampling. Published evidence indicates that concentrations of oxygenated monoterpenes associated bark beetle-infested (and dead, uninfested) conifer tissue continue to increase after 18 d. This is significant since the bark quickly becomes unsuitable substrate for bark beetle reproduction following the initiation of bark beetle attacks. This unsuitability is associated with the rapid colonization of the bark by deleterious bluestain fungi, which is completed typically in 3-4 wks following tree colonization. Hence, the temporal pattern of increase of oxygenated monoterpenes observed by the authors and other studies does not correspond with the period of greatest host suitability for bark beetles, but rather the opposite.

We agree with the reviewer that the timing of volatile emission during fungal infestation is important for understanding its influence on bark beetle behavior, and that emission is likely to continue to increase after 18 days of fungal growth in intact trees. We measured volatiles only up to 18 days since after that time, the bark plugs we used were completely colonized by fungi. We have now rephrased the sentence on line 141 clarifying that the “18 d maximum increase” was during a laboratory experiment on detached bark plugs, and the time course may not strictly apply to the time course of emission from an intact tree, which might continue to increase for a longer time.

We do not feel that blue-stain fungi are as detrimental to bark beetles as the reviewer suggests since most of the published data runs contrary to this thesis. Instead, we prefer the concept that the roles of blue-stain fungi are contextual and vary with the beetle species (Six and Klepzig, 2021). Nevertheless, in support of the reviewer’s thesis, we do show that oxygenated monoterpenes repel bark beetles at high concentrations (Figs. 5B, 5F) and thus high densities of fungi in the bark could signal detrimental conditions for the beetle. We speculate that these are an indicator of crowding. A high emission could be recognized by incoming conspecifics beetles to avoid bark sections already densely colonized by beetles. Although we feel that blue-stain fungi may still benefit *I. typographus* in various ways, we agree with the reviewer that the highest emission may not always correspond to the highest host tree suitability, at least for newly arriving beetles. We discuss these points now in lines 445-450.

Mass attack by aggressive bark beetle species is typically completed in days, not weeks. However, the authors note that their behavioral bioassay results suggest that positive responses occur only at intermediate concentrations of the oxygenated monoterpenes (note that the authors fail to measure natural concentrations, hence "high" and "intermediate" may have no relevance in nature). The authors note that bark beetle parasitoids appear to be attracted to oxygenated monoterpenes, but parasitoids seek beetle brood (the parasitoids' hosts) in the later stages of development, at which time new attacks have generally ceased.

The reviewer is correct that mass attack of aggressive bark beetle species can be completed in days, but this varies with the beetle population phase. The colonization of a host tree occurs within days during the epidemic population phase, when the beetle population is high. However, in the endemic phase, the colonization of a single tree typically takes several weeks depending on the beetle population in the vicinity (*Toffin et al., 2018*). In such a scenario, oxygenated monoterpenes produced by fungi introduced by early colonizers could assist the conspecifics to identify the host. We have now expanded our discussion in lines 424-436 to relate the effects of volatile emission to population phase. Our classification of high, intermediate, and low concentrations of oxygenated monoterpenes in dose response bioassays, as mentioned by the reviewer, are relative as this is a laboratory assay and does not perfectly reflect the natural concentrations in the bark. However, in Fig 5A,C,E and F we used natural concentrations of precursor monoterpenes in the diet colonized by fungi and tested the response of adult beetles to volatiles emitted from these media. We have now revised the text in lines 271 and 289 to indicate which concentrations are natural.

L172-194 The ability of bark beetle associated fungi to generate oxygenated monoterpenes from hydrocarbon monoterpenes in vitro has been demonstrated numerous times, and the capacity appears to not be limited to just bark beetle symbionts (e.g., fungal parasites in the genus Trichoderma, which invade the bark after beetle brood development is completed, can do this too). The authors interpret this result as if this were an in vivo study (L192 - 194), which is erroneous.

We completely agree with the reviewer that production of oxygenated monoterpenes from monoterpene hydrocarbons is not unique to ophiostomatoid fungi, and our additional experiment suggested that *Trichoderma sp.* can also produce some these compounds but with a different composition and quantity compared to the profiles produced by the ophiostomatoid fungi used in this study. As we show, *I. typographus* bark beetles were attracted to the volatile profiles of bark beetle-associated fungi, but were unresponsive to the volatile profile of the *Trichoderma* sp. we isolated. We have now expanded our results and discussion in lines 181-185, lines 187-188, lines 384-388 to include these new experiments.

L195 - 244 This is a very interesting, novel, and well-conducted component of the study. I would like to see it published as a separate manuscript, albeit in a journal that is narrower in scope. However, it provides no evidence that would particularly support the thesis of the paper. Presence of olfactory sensitivity to oxygenated monoterpenes is not surprising since many of these compounds are produced by the beetles themselves (some are demonstrated pheromones). Electroantennogram (whole antenna and SSR) studies do not distinguish repellants from attractants so functionality of the oxygenated monoterpenes in beetle biology is not illuminated. Furthermore, the very limited specificity (just three classes) of the ORNs for the dozens of oxygenated monoterpenes identified in the study would seem to suggest that there is little specificity in response to them (i.e., the beetles lack the capacity to readily distinguish among the compounds, and behavioral responses are likely relatively indiscriminate). It is noteworthy that I. typographus antenna were not exceptionally sensitive to the oxygenated monoterpenes that appeared to stand out as a product of G. penicillata (e.g., camphor).

We thank the reviewer for the positive comments and interest in this experiment. Combining our previous study (*Kandasamy et al., 2019*) with the current results, we have now detected 9 classes of olfactory sensory neurons that respond chiefly to oxygenated monoterpenes: *cis*-verbenol, (-)-ipsenol, (+)-ipsdienol, (-)-ipsdienol, amitinol, isopinocamphone, 4-thujanol, verbenone and 1,8-cineole. The reviewer is correct that many of these compounds are produced by the beetles themselves. Based on knowledge of the sources of these compounds, from the literature and our data, the first 5 are produced primarily from beetles, the second 3 are fungal products (described in this manuscript) and the last is a host tree product. The different OSN classes appear to be specifically tuned to detect odorants from different ecological sources. However, the picture may be more complex since some of the fungus-produced oxygenated monoterpenes elicit secondary responses from other OSN classes that respond primarily to host and other compounds. We do not share the reviewer’s opinion that *I. typographus* lacks specificity for fungus-produced oxygenated monoterpenes merely because it has only three OSN types to detect most of these odorants. Specificity is not necessarily determined by individual OSNs. It has been shown previously that insects process the olfactory information from their environment through combinatorial code since a single olfactory neuron often can be activated by several to many odorants and a single odorant can often activate multiple OSNs. Moreover, information from the peripheral sensilla is further processed in the higher brain centers and the valence of a compound or mixture depends on which olfactory circuit(s) a given odorant(s) can activate. We have now elaborated on some of these points in the discussion (lines 566 and following).

The generality of antennal sensitivity to oxygenated monoterpenes fits well with the oft-cited hypothesis that these compounds - which are well known to increase in concentration as host conifers decay and become unsuitable - are indicators of host unsuitability for flying beetles. The capacity of many of these compounds to reduce bark beetle responses to aggregation attractant support this hypothesis. Hence there are multiple plausible hypotheses for why I. typographus possess olfactory sensitivity to oxygenated monoterpenes, and there is no strong argument for favoring fungal interactions in particular.

The reviewer is correct that some of the oxygenated monoterpenes do indicate unsuitability to adult *I. typographus* bark beetles. Verbenone, ipsenol, 1,8-cineole, and 4-thujanol when combined with pheromones, have been shown to inhibit the attraction of *I. typographus* to its pheromone (*Bakke 1981; Andersson et al., 2010; Jirošová et al., 2021*). On the other hand, *cis*-verbenol and camphor have been shown to improve the attraction of dispersing beetles in field trapping experiments (*Bakke 1976; Kalinová et al., 2014*). Furthermore, the colonization success of dispersing beetles has been shown to be positively correlated with the increasing concentration of oxygenated monoterpenes in trap trees (*Schiebe et al., 2012*). Therefore, the oxygenated monoterpenes found in conifer bark, fungi and beetles may have varying roles for *I. typographus* depending on their structures, stage of attack and the olfactory sensitivity of OSNs. Since we showed the profiles of oxygenated monoterpenes are distinct among fungal symbionts, saprophytes, and bark beetles, we suggest that bark beetles possess different classes of OSNs to detect compounds from multiple ecological sources including fungi and can distinguish symbionts versus saprophytes based on their volatile profiles. We have now incorporated these points in the discussion in lines 454 and following, and 578 and following.

L245-281 The results of these tests are uncompelling. Responses to the oxygenated monoterpenes were not consistent among compounds or doses. Given the sheer number of contrasts, the apparent absence of an adjustment for experiment-wise error, and the relatively high P-values for most significant responses, the few significant effects detected are likely spurious and are suggestive at best.

We thank the reviewer for pointing out a potential problem with results of Fig.4. We have shown that two of the four individual oxygenated monoterpenes tested were attractive to beetles. However, the mixture of these metabolites produced from spruce resin precursors is strongly attractive when presented in the composition ¸ and at the dose produced by each fungus. The idea to test for experiment-wise error is a good suggestion, given the number of contrasts made. However, we could not perform a Bonferroni test to make such corrections here as we only performed a single pairwise non-parametric Wilcoxon’s test (choice of beetles between control and treatment) for each experiment.

Since the above are more than sufficient reasons for rejection of the paper, I made no efforts to critique other aspects of the work.

We would like to thank the reviewer for spending time to review our manuscript. The critical comments have prompted us to conduct more experiments, which we feel make our overall manuscript more convincing.

Reviewer #3:

The manuscript 'Bark beetles locate fungal symbionts by detecting volatile fungal metabolites of host tree resin monoterpenes …' makes significant contributions to our understanding of bark beetle-fungal-conifer interactions, chemical ecology, and insect behavior. The topic is appropriate for and of interest to the readers of PLOS Biology.

The paper's greatest strength is its broad range and diversity of assays - chemical, behavioral, electrophysiological. These experiments are arranged in a very complementary fashion, and each experiment is conducted well with no major weak link among them.

Before this is acceptable for publication, a few changes need to be made. There are two major needs:

1. The ecological context of the role fungi play in aggregation needs to be better articulated. It appears, but is not clear whether, the authors are interpreting these bioassay results to mean metabolism of host terpenes by beetle-introduced fungi contribute to the mass attack behavior by which bark beetles overwhelm tree defenses. If so, the chronology of fungal culturing in lines 554-558 does not support this. Beetle mass attacks must occur very quickly in order for their numbers to negate rapidly induced defenses. The time frame required for fungal establishment described in lines 554-558 is much longer. So a context appropriate to this time course should be established. Perhaps rather than facilitating mass attacks on healthy trees during outbreaks these findings are more relevant during the long intervals between outbreaks when beetle persistence is especially precarious. During these periods I. typographus develop mostly in windthrown trees or trees experiencing severe drought. Under those conditions I. typographus can arrive more gradually and still succeed. Here the introduction and growth of fungi might more easily contribute to further successful beetle arrival. Or other bark beetle species carrying some of these same or similar fungi might cohabit these stressed or windthrown trees (e.g., Rouault 2006). There is substantial overlap among fungal species carried by co-occurring bark beetles in severely stressed trees.

We thank the reviewer for this very insightful comment on the timing of fungal colonization in relation to beetle attack. We agree that the results from our bioassays may be more relevant to an endemic beetle population where the colonization of a whole tree takes months. However, even during epidemics, fungal volatiles could be perceived by incoming beetles within a few days of introduction of fungi by early colonizers. Although we did not measure fungal volatiles prior to 4 days after inoculation, previous studies have shown that fungi emit volatiles from the first day of attack (*Zhao et al., 2015, 2019*). Therefore, the role of fungal volatiles for epidemic beetle populations cannot be ruled out. However, for both endemic and epidemic populations, fungal volatiles from established galleries could be perceived as an aversive signal to redirect beetles to uncolonized sections of the bark or to a new tree. We have now expanded our discussion to include these points in lines 424-436. To get more insight into the excellent question raised by the reviewer, we are currently performing field experiments to discover which life stage of the fungus is attractive or repulsive to beetles using fungus cultures of different ages together with pheromones as baits in traps. Such experiments (now partially published, with the results mentioned in lines 413-416) will provide more insight into the timing of fungal volatiles and their effect on the behavior of beetles at different population phases.

2. The manuscript describes 'microbes' throughout, yet no mention is made that bacteria associated with bark beetles have already been shown to metabolize conifer terpenes into bark beetle pheromones (e.g., Xu et al. 2015, 2016; Brand et al. 1975, Byers & Wood 1981). Also, beetle-associated bacteria have been shown to influence interactions between beetle-associated fungi and conifer monoterpenes (e.g., Adams et al. 2009). These features should be incorporated into the description of prior knowledge and interpretation of ecological context.

We thank the reviewer for pointing out our omission of this topic. We agree with the reviewer that there is extensive research on the role of bacteria in the metabolism of host monoterpenes, and we have now included these studies in the discussion lines 529-533.

There are also several additional issues:
1. In Figure 4, the treatments are continuous variables but they are presented and analyzed as categorical values. These are essentially dose-response curves. So it is better to analyze these data with some form of global analysis, such as Preference vs. dose.

We thank the reviewer for pointing out potential problems with the statistical analysis of bioassay data in Fig. 4. However, each treatment in figure 4 was a separate experiment, and therefore, we treated each as a categorical variable and performed a paired-sample non-parametric test to test the choice of beetles between the two options.

2. In a number of cases the broad term 'bark beetle' is used where the more specific term 'I. typographus' would be more appropriate. Bark beetles are a very large and diverse group and vary substantially in their host relationships, signaling behavior, and symbioses. In some cases the term 'bark beetle' is fine, but particularly in the Results, Figures, Figure captions, etc. where the data are referring to these particular experiments 'I. typographus' is better.

We completely agree with the reviewer and apologize for our oversight. We have now tried to specify at every opportunity that this study focuses only on *I. typographus*.

3. The bioassay methods described in sections 1.3 and 1.10 have been developed previously by several other lab groups elsewhere, so it would be good to cite those.

We appreciate the fact that some of these bioassay methods have been developed in other research groups before we employed them and have now added a reference to the text (line 802).

4. The manuscript is well-written overall, but the Results section contains substantial repetition of Methods. This is rather distracting and adds unnecessary length. Specifically, every subsection of the Results begins by deviating from the experimental results and adding such redundancy: e.g., Line 117, 132, 174-178, 182, 246-249, 283-288, etc., etc.). Please delete.

We have now removed the introductory sentences at the beginning of each subsection of the Results section, and insured that this information is contained in the Methods section.

5. Similar to above, the Results section contains substantial overlap with the Discussion. Conclusions such as 'This indicates that', etc. should be removed from the Results. Some examples are Line 124, 192-194, 240-244, 278-281, etc.

We have now removed the concluding sentences from each subsection of the Results and insured that this information is contained in the Discussion.

6. I'm not sure what is meant by Other factors … 'may determine the valence of these compounds.' How do these factors influence 'valence'? Please clarify.

We have now changed this sentence and added information on the specific factors that may influence the valence of compounds to beetles.

7. Lines 457-458. But one thing to consider here is that these trees also contain diterpenes, and diterpenes much more toxic to fungi than either oxygenated or nonoxygenated monoterpenes.

The reviewer is absolutely correct in pointing out that diterpenes could be more toxic to fungi than other terpenes. We have now mentioned this fact in the Discussion following line 492.

I also have two suggestions that the authors may feel free to implement or discard as they see fit.
1. Fig. 2 is somewhat overwhelming. There is a lot of good information here, and I fear some of it will be lost on readers because they cannot digest it all at once. Consider splitting this into several figures, based on the type of data.
2. Consider a summary table in the Discussion showing the different effects of the different fungi. Yes, that would be repetitive of what is shown across individual experiments but I think a table like this could really highlight what I see as this paper's greatest strength, the integration of different categories of fungal - beetle - chemical interactions. Such a table could allow readers to see at a glance which fungal species are playing which roles.

We have now split Fig.2 into two separate figures as suggested by the reviewer.
