# Supplementary material for: Conifer-killing bark beetles locate fungal symbionts by detecting volatile fungal metabolites of host tree resin monoterpenes
Source: PLoS Biol. 2023 Feb 21;21(2):e3001887. doi: 10.1371/journal.pbio.3001887 (PMC9943021; doi:10.1371/journal.pbio.3001887)
Supplement: Attachment — Submitted filename: Second revision Response letter to reviewers.docx [file pbio.3001887.s031.docx]

Dear Dr Gershenzon,

Thank you for your patience while we considered your revised manuscript "Bark beetles locate fungal symbionts by detecting volatile fungal metabolites of host tree resin monoterpenes" for publication as a Research Article at PLOS Biology. This revised version of your manuscript has been evaluated by the PLOS Biology editors, the Academic Editor, and the three original reviewers.

Based on the reviews, we are likely to accept this manuscript for publication, provided you satisfactorily address the remaining points raised by the reviewers. Please also make sure to address the following data and other policy-related requests.

IMPORTANT:

a) Please make the title more explicit; we also think that it would be helpful to emphasise the importance of the study species - overall, we suggest something like "Conifer-decimating bark beetles locate fungal symbionts by detecting volatile odorants generated by fungal metabolism of tree resin monoterpenes"

We have now revised the title to emphasize all the study species.

b) Please attend to the remaining requests from reviewers #2 and #3. Most of these relate to toning down the strength of the claims, and although reviewer #2's comments are lengthy, s/he is overall very positive, and I believe that the requests are mostly textual and/or presentational in nature.

Please see our point-by-point response to the reviewers below.

c) Please address my Data Policy requests below; specifically, we need you to supply the numerical values underlying Figs 1BCD, 2ABCDE, 3A, 4B, 5BCD, 6ABCDEF, 7AB, 8BCD, S1, S2, S3, S4, S5, S6, S7, S8, S9, S10ABC, S11, S12ABCDEFGHIJ, S13BCDEF, S14BCDEF, either as a supplementary data file or as a permanent DOI’d deposition.

The raw data for all the figures and tables can be found at https://doi.org/10.6084/m9.figshare.21692156.v1

d) Please cite the location of the data clearly in all relevant main and supplementary Figure legends, e.g. “The data underlying this Figure can be found in S1 Data” or “The data underlying this Figure can be found in [https://doi.org/XXXX](https://doi.org/XXXX)”

We have cited the location of raw data in all main and supplementary figures and table legends.

As you address these items, please take this last chance to review your reference list to ensure that it is complete and correct. If you have cited papers that have been retracted, please include the rationale for doing so in the manuscript text, or remove these references and replace them with relevant current references. Any changes to the reference list should be mentioned in the cover letter that accompanies your revised manuscript.

We have removed one reference (Boone et al. 2013) and added seven new references (Keefover-Ring et al. 2016; Zhao et al. 2011; Cardoza et al. 2006; Zhao et al. 2010; Salom et al. 1991; Mikami et al. 1988; Krings and Berger 2010) as requested by the reviewers.

We expect to receive your revised manuscript within two weeks.

To submit your revision, please go to [https://www.editorialmanager.com/pbiology/](https://www.editorialmanager.com/pbiology/) and log in as an Author. Click the link labelled 'Submissions Needing Revision' to find your submission record. Your revised submission must include the following:

-  a cover letter that should detail your responses to any editorial requests, if applicable, and whether changes have been made to the reference list

-  a Response to Reviewers file that provides a detailed response to the reviewers' comments (if applicable)

-  a track-changes file indicating any changes that you have made to the manuscript.

NOTE: If Supporting Information files are included with your article, note that these are not copyedited and will be published as they are submitted. Please ensure that these files are legible and of high quality (at least 300 dpi) in an easily accessible file format. For this reason, please be aware that any references listed in an SI file will not be indexed. For more information, see our Supporting Information guidelines:

[https://journals.plos.org/plosbiology/s/supporting-information](https://journals.plos.org/plosbiology/s/supporting-information  )

*Published Peer Review History*

Please note that you may have the opportunity to make the peer review history publicly available. The record will include editor decision letters (with reviews) and your responses to reviewer comments. If eligible, we will contact you to opt in or out. Please see here for more details:

<https://blogs.plos.org/plos/2019/05/plos-journals-now-open-for-published-peer-review/>

*Press*

Should you, your institution's press office or the journal office choose to press release your paper, please ensure you have opted out of Early Article Posting on the submission form. We ask that you notify us as soon as possible if you or your institution is planning to press release the article.

*Protocols deposition*

To enhance the reproducibility of your results, we recommend that if applicable you deposit your laboratory protocols in protocols.io, where a protocol can be assigned its own identifier (DOI) such that it can be cited independently in the future. Additionally, PLOS ONE offers an option for publishing peer-reviewed Lab Protocol articles, which describe protocols hosted on protocols.io. Read more information on sharing protocols at [https://plos.org/protocols?utm_medium=editorial-email&utm_source=authorletters&utm_campaign=protocols](https://plos.org/protocols?utm_medium=editorial-email&utm_source=authorletters&utm_campaign=protocols)

Please do not hesitate to contact me should you have any questions.

Sincerely,

Roli Roberts

Roland Roberts, PhD

Senior Editor,

[rroberts@plos.org](mailto:rroberts@plos.org),

PLOS Biology

------------------------------------------------------------------------

DATA POLICY:

You may be aware of the PLOS Data Policy, which requires that all data be made available without restriction: [http://journals.plos.org/plosbiology/s/data-availability](http://journals.plos.org/plosbiology/s/data-availability). For more information, please also see this editorial: [http://dx.doi.org/10.1371/journal.pbio.1001797](http://dx.doi.org/10.1371/journal.pbio.1001797)

Note that we do not require all raw data. Rather, we ask that all individual quantitative observations that underlie the data summarized in the figures and results of your paper be made available in one of the following forms:

1) Supplementary files (e.g., excel). Please ensure that all data files are uploaded as 'Supporting Information' and are invariably referred to (in the manuscript, figure legends, and the Description field when uploading your files) using the following format verbatim: S1 Data, S2 Data, etc. Multiple panels of a single or even several figures can be included as multiple sheets in one excel file that is saved using exactly the following convention: S1_Data.xlsx (using an underscore).

2) Deposition in a publicly available repository. Please also provide the accession code or a reviewer link so that we may view your data before publication.

Regardless of the method selected, please ensure that you provide the individual numerical values that underlie the summary data displayed in the following figure panels as they are essential for readers to assess your analysis and to reproduce it: Figs 1BCD, 2ABCDE, 3A, 4B, 5BCD, 6ABCDEF, 7AB, 8BCD, S1, S2, S3, S4, S5, S6, S7, S8, S9, S10ABC, S11, S12ABCDEFGHIJ, S13BCDEF, S14BCDEF. NOTE: the numerical data provided should include all replicates AND the way in which the plotted mean and errors were derived (it should not present only the mean/average values).

IMPORTANT: Please also ensure that figure legends in your manuscript include information on where the underlying data can be found, and ensure your supplemental data file/s has a legend.

Please ensure that your Data Statement in the submission system accurately describes where your data can be found.

------------------------------------------------------------------------

SPECIES INDICATED IN THE ABSTRACT?

- Please note that per journal policy, the model system/species studied should be clearly stated in the abstract of your manuscript.

------------------------------------------------------------------------

BLOT AND GEL REPORTING REQUIREMENTS:

We require the original, uncropped and minimally adjusted images supporting all blot and gel results reported in an article's figures or Supporting Information files. We will require these files before a manuscript can be accepted so please prepare and upload them now. Please carefully read our guidelines for how to prepare and upload this data: [https://journals.plos.org/plosbiology/s/figures#loc-blot-and-gel-reporting-requirements](https://journals.plos.org/plosbiology/s/figures#loc-blot-and-gel-reporting-requirements)

------------------------------------------------------------------------

DATA NOT SHOWN?

- Please note that per journal policy, we do not allow the mention of "data not shown", "personal communication", "manuscript in preparation" or other references to data that is not publicly available or contained within this manuscript. Please either remove mention of these data or provide figures presenting the results and the data underlying the figure(s).

------------------------------------------------------------------------

REVIEWERS' COMMENTS:

Reviewer #1:

The authors have addressed all of my concerns and appear to have addressed the comments, suggestions, concerns, and suggested experiments of the other reviewers.

I enjoyed reading/reviewing the ms.

We thank the reviewer for their comments that undoubtedly improved the quality of our manuscript.

Reviewer #2:

The authors have done an amazing job of addressing my concerns with the paper.  I strongly recommend publication if the authors can address my further comments and recommendations below, and these can be addressed easily.

One of my original concerns remains - the authors appear to want to force their data into supporting an overly-simplified narrative regarding the chemical ecology of the beetle/symbiont relationship.  Several of the experiments do not support their conclusions but are presented as if they do (discussed below).  The paper focuses on attraction when clearly oxygenated monoterpenes can have both attractive and repellent effects (this is mentioned but appears to be ignored in the title and elsewhere). There is no conclusive support for the assertion that Ips typographus utilize fungus-produced oxygenated monoterpenes to locate host tissue colonized by beneficial fungi.  I do think something very interesting is going on in this system, but their data are not pointing to a single possible narrative.  If the authors amend the paper to interpret and present their excellent experiments in a way that considers all possible explanations and ramifications, I think this work will be an outstanding contribution.

We once again thank the reviewer for critically reading our manuscript and providing insightful comments that undoubtedly improved its quality. Please see our point-by-point responses below.

Here are my comments on the authors' responses to my criticisms:

1)	My criticism:  Deleterious fungal associates are common, particularly bluestain.  Authors' response: some bluestain fungi are beneficial.  My response: The authors are correct about the existence of beneficial bluestain fungi; this was my error.  However, my point stands that there is no general rule that fungal associates that are carried by the beetle can be assumed to be beneficial.  The bluestain O. minus is carried by and highly deleterious to brood development (larvae die) of both bark beetles Dendroctonus frontalis and D. brevicomis and the bluestain Ophiostoma ips is deleterious to Ips grandicollis and Ips calligraphus host colonization and brood development.  Ophiostoma ips appears to be beneficial to Ips pini, however, which suggests that generalizations within a fungal genus cannot be made.  Too few systems have been studied sufficiently to make a generalization about whether fungal associates are beneficial or deleterious.  The authors acknowledge as much and that the ecological relationships between Ips typographus and their fungal associates have not been studied sufficiently to draw conclusions.

2)	My criticism:  Deleterious fungi were not studied to see if they also produce oxygenated monoterpenes.  Authors' response:  A new study looked at a deleterious fungal species (Trichoderma) and found it to produce oxygenated monoterpenes but a different blend than produced by G. penicilliata.  In a behavioral bioassay, Trichoderma cultures were unattractive to beetles whereas cultures of a symbiont were.  My response:  I am extremely pleased that they did this experiment.  As a graduate student, I compared volatiles arising from symbionts of D. frontalis and Trichoderma harzianum (in my dissertation but unpublished) and also found different blends of oxygenated monoterpenes.  There is no question that Trichoderma is not a symbiont and my personal observations indicate that it is deleterious (the mycelium appears capable of infecting beetle brood pupae), although there is nothing I know of published on the topic.  Their choice of a "control" fungal species was perfect, I think.  However, their argument about the role of oxygenated monoterpenes being the compounds mediating attraction of bark beetles to fungal cultures hinges on the attraction being to specific blends of oxygenated monoterpenes.  This is a reasonable hypothesis, but they provide no evidence for it specifically.  Additionally, they provide no evidence that oxygenated monoterpenes are the semiochemicals mediating attraction of the beetles to fungal cultures.  The fungi produce other compounds, some of which are listed in their very complete tables in the supplementary materials.

We thank the reviewer for stimulating us to perform this experiment and also observed the deleterious effect of *Trichoderma*, especially on pupae. We encourage the reviewer to publish her/his old data!

## The reviewer is correct that, while we do show that *Trichoderma* cultures are not attractive to bark beetles and produce different oxygenated monoterpenes than the attractive symbiont *Grosmannia penicillata*, this does not prove that differences in oxygenated monoterpenes are responsible. However, we disagree that we have “no evidence that oxygenated monoterpenes are the semiochemicals mediating attraction of beetles to fungal cultures”. Emission of oxygenated monoterpenes is well-correlated with beetle attraction (Figs. 6A, 6C), and in pure form two individual oxygenated monoterpenes were attractive (Figs. 6B, 6D). Nevertheless, we have now rewritten this section as described below under point #7 as well as revised the text, in general, to make sure we do not overstate the case for oxygenated monoterpenes in attraction. We also emphasize that the entire volatile profile of fungi including other volatile groups listed in Tables S3-6 determines bark beetle attraction, not just oxygenated monoterpenes (lines 419-424,436-442-399). In addition, we now add that oxygenated monoterpenes could act as repellent cues to beetles in this system. We thank the reviewer for his/her continued critical focus. This has helped us to take a well-balanced view of our results.

3)	My criticism: Oxygenated monoterpenes have many different sources in the environment including the beetles, and therefore, as a class, they cannot be termed "fungal metabolites".  Authors' response (1):  Ips typographus do not produce the major oxygenated monoterpenes detected from fungal cultures.  My response:  This is largely accurate, and in the discussion the authors cover this topic well.  If the arguments stay limited to Ips typographus, there isn't a problem. If the discussion is expanded to other bark beetle species, there is substantial overlap between oxygenated monoterpenes of beetles and fungi.    Authors' response (2):  Other sources exist but the blends produced by the different sources differs, and the beetles are responding to different blends.  My response:  The authors have no evidence for the latter assertion. It would need support from tests showing that (1) synthetic blends of oxygenated monoterpenes produced by fungi are attractive to Ips

typographus, (2) the beetles distinguished among blends of oxygenated monoterpenes, (3) the beetles distinguished blends associated specifically with their fungal symbionts.   Their existing behavioral tests don't address these questions.

We agree with the reviewer that testing the response of beetles to synthetic blends of oxygenated monoterpenes is the only way to ensure that other compounds are not involved. Unfortunately, not all the oxygenated monoterpenes detected in our work are available for testing and we did not carry out experiments with synthetic blends, only single compounds. Of the individual oxygenated monoterpenes tested, two were attractive at specific concentrations and two showed no response at the concentrations tested. We have now revised the text to avoid the general claim that beetles are employing oxygenated monoterpene blends to distinguish among fungal types.

4)	My criticism:  The authors' "fungal metabolites" may not be produced by the fungi alone but possibly also reaction by the infested plant tissue to fungal infection.  Authors' response:  This is as real possibility, however, testing it is extremely challenging, and our own efforts were inconclusive.  My response:  I am a little confused about why they think this remains an open question, because their new experiment in which sterile and re-inoculated beetles are introduced into logs addresses this topic well.  This particular experiment is very informative and helpful to a range of important questions about bark beetle-host interactions, I and applaud the authors for doing this.  I am sorry there was not more replication.

We thank the reviewer for their appreciation of our experiment. However, we are still a bit hesitant to use the results to indicate that our “fungal metabolites” are definitely of fungal origin and do not arise from the tree itself. It is conceivable that the tree may produce such metabolites, but only when infected by fungi. This point is now mentioned in the discussion section, and we end with a call for more research.

5)	 My criticism:  The work is not original in stating that fungal development is responsible for oxygenated monoterpenes has been published.  Authors' response: "Thus, we conclude that ours is indeed the first study to show that symbiotic ophiostomatoid fungi are the main source of I. typographus-attractive oxygenated monoterpenes in Picea abies bark." My response:  In retrospect, I was excessively harsh in my criticism regarding originality.  My criticism was based on the fact that several studies have shown that levels of oxygenated monoterpenes - including the major ones identified in the authors' study - increase in the days and weeks following bark beetle attack, and authors have attributed this effect to fungal growth.  However, the latter was never demonstrated experimentally.   Demonstration that fungi (yeasts) can produce oxygenated monoterpenes had previously occurred only in Leufvén et al., 1987, as indicated by the authors.  A fair statement is that the hypothesis is not original, but research into the hypothesis has never before been so thorough (particularly in the revised version of the paper) and involved mycelium-producing fungi.  I have changed my mind, and I agree that the work is sufficiently original to merit publication.

We were glad to read the reviewer’s comments and have now revised the text to make sure we attributed the original hypothesis to previous works.

6)	 My criticism:    The authors fail to account for the fact that one of the experimental contrasts they use as evidence is not sufficiently controlled, as different media were used for experimental (SBA) and "blank" treatments (PDA), and the result was thus inappropriately used to argue that attraction was due to fungal inoculation in the experimental cultures.  Authors' response:  Additional tests indicate that the SBA alone is attractive but it is "more reasonable" to conclude that the response was to attractive compounds.  My response:  It is clear from Fig 1D that the spruce bark itself is attractive (important information), but none of the data  interpretation rests on experiments where effects of the medium and fungal treatment are conflated.  This is good now.

7)	My criticism:    Experiments do not demonstrate that fungal production of attractants/feeding stimulants increases in the presence of hydrocarbon monoterpenes from the host, a conclusion that would require demonstration of a statistical interaction between presence of monoterpene and fungus.  Authors' response:   There was no statistical evidence of an interaction, but "we could clearly show that the presence of the fungus in a beta-pinene enriched diet resulted in significantly longer tunnel length (Fig. 6B)". My response: This is still a significant problem. Figure 6A (not 6B, authors' typo) and 6C only show that the result in figure 1A (PDA cultures are more attractive with growth of G. penicillita) is also observed when the plates contain SPA with either beta-pinene or bornyl acetate.  The attraction can be attributed to the fungus alone, hence there is no evidence that presence of beta-pinene or bornyl acetate has a  role in changing the attractiveness of fungal plates.

Figures 6E and 6F supports the conclusion that the presence of beta-pinene and bornyl acetate have no effect (the combination of fungus and monoterpene is not more attractive than fungus alone).  Hence figures B and D are attempting to explain a phenomenon that was never demonstrated in the first place.

 This does not mean that the results of 6B and D aren't interesting - however.  I think that presentation of results that do not support the authors' thesis is important, but figures 6A, C, E and F provide no support to the statement that "Ips typographus is attracted to oxygenated monoterpenes produced by symbiotic fungi" (the title for Figure 6).   An additional problem with the title is that repellency was also observed.  The figure title and the included data in the figure must be changed.

 We understand how the reviewer sees Figs. 6A and 6C as not showing much more than Fig. 1. We now present these results differently, not as proving the attraction of specific volatiles, but as providing motivation for further experiments. Figs. 6A and 6C illustrate that beetle attraction is correlated with the presence of oxygenated monoterpenes (as shown in the inset of the chromatogram), and this motivated us to then test some of the pure oxygenated monoterpenes available (Fig. 6B, 6D). The results showed that two were attractive and two without effect at any tested concentration. Figs. 6E and 6F, Fig S11 allow us to exclude that the precursor monoterpene olefins are attractive, so we can then conclude that the derived oxygenated metabolites could represent attractive cues. We have also now revised the title of Fig. 6.

8)	My criticism:  It is possible that oxygenated monoterpenes could be produced by the host tissue also (second mention of this).  Authors' response:   This is a difficult topic to explore.  Also: "We found species-specific VOC profiles for the different fungi when they colonized spruce bark. These seem more likely to occur if the compounds were made by the fungus. If these compounds were produced by the tree as a defense response, one might expect a very similar response to each of these ophiostomatoid fungi."  My response:  I am not sure why production of species-specific oxygenated monoterpene blends by the fungi in vitro is relevant to this point.  I don't think the existence of variation by itself implies that the variation must have some ecological significance, and, in particular, mediation of host selection by bark beetles.  It opens the possibility, but is not an argument in support of it.

We understand the reviewer’s point and have now removed the speculation that specific volatile profiles from different fungi support fungal production of oxygenated monoterpenes.

9)	My criticism:  The authors claim that oxygenated terpene levels reached a maximum at 18 days is not supported by the data since a longer interval was not tested.  Also, it has been shown that concentrations of oxygenated monoterpenes continue to increase in host bark well after the bark is unsuitable for further colonization (no further attacks would be occurring).  Authors' response:   Text is changed to indicate that the study was the result of a laboratory experiment and that the fungi might also mediate rejection of exhausted host material.  My response:  Sounds good.  I agree that bluestain fungi are not always bad for bark beetles, but too few systems have been studied to make generalizations.

10)	My criticism: Mass attack by aggressive species of bark beetles happens before the associated fungi are established.  Authors Response:  True, but only during epidemics. When beetle populations are low, colonization occurs slowly, and thus trees with some fungal colonization are still be attacked.  My response:  This is true and is an excellent point.  However, the implication is that fungal activity can have little if any influence on host selection during epidemics, when bark beetles are inflicting their greatest harm.  The authors do address this in the discussion.

11)	My criticism: The olfaction studies are well-done and interesting but provide, at best, weak support for the thesis.  They suggest relatively limited olfactory specificity to oxygenated monoterpenes and relatively low olfactory sensitivity to those produced in greatest quantity by fungal cultures. Authors Response:  Nine classes of ORNs are sensitive to oxygenated monoterpenes.  My Response:  As the authors indicate, five are specific to beetle pheromones.  Of three ORN classes described as fungi-specific,  the verbenone group includes bark beetle pheromones (including cis-verbenol) and compounds with other origins (both trans-verbenol and verbenone can arise from autoxidation of alpha-pinene).  The isopinocamphone and trans-4-thujanol groups are harder to explain.  Authors Response:  Limited numbers of ORNs can nonetheless permit discrimination of compounds.  My Response:  I agree, it is hard to translate numbers or ORN classes into the capacity to distinguish compounds

or blends.

12)	My Criticism:  The presence of olfactory receptors to oxygenated monoterpenes is no argument in itself that these compounds mediate attraction of beetles to host trees colonized by fungal symbionts.  These compounds may also function in signaling unsuitability, and there is compelling evidence for this.  Authors' Response: "Since we showed the profiles of oxygenated monoterpenes are distinct among fungal symbionts, saprophytes, and bark beetles, we suggest that bark beetles possess different classes of OSNs to detect compounds from multiple ecological sources including fungi and can distinguish symbionts versus saprophytes based on their volatile profiles. We have now incorporated these points in the discussion in lines 454 and following, and 578 and following."  My Response:  Sounds good.

13)	 My Criticism: Results of the behavioral bioassays of response of beetles to individual monoterpenes is uncompelling due to the large number of tests with only two showing significance and at a single, intermediate dose.  Authors' Response:  However, the mixture of these metabolites produced from spruce resin precursors is strongly attractive when presented in the composition and at the dose produced by each fungus.  Reviewer Response:  Where is this experiment in the paper?

We refer to Figures 6A and 6C here, but as stated above, we accept the reviewer’s assertion that these experiments show attraction to the total fungal volatile mixture, and not to any specific compounds. We now present these data differently as showing how attraction is correlated with the presence of oxygenated monoterpenes in the gas chromatogram. We then used these results as the motivation to test individual oxygenated monoterpenes in pure form (Figs. 6B, 6D).

Authors' Response:  However, we could not perform a Bonferroni test to make such corrections here as we only performed a single pairwise non-parametric Wilcoxon's test (choice of beetles between control and treatment) for each experiment.  Reviewer Response: The Bonferroni correction can be applied to any number of tests in an experiment, although typically its use is limited to multiple comparisons of treatments in a single experiment.  The principal behind the test is still valid in other situations - the more tests that are run, the more likely that one will see a type 1 error, and this likelihood can be calculated by dividing the alpha level by the number of tests.  Although statistical practice does not mandate application of a Bonferroni correction to the authors' data, the principal nonetheless influences interpretation of the data and demands skepticism be given to the broad statement that  "Ips typographus is attracted to oxygenated monoterpenes produced by symbiotic fungi".  There were 21 separate tests looking at beetle response to individual oxygenated monoterpenes (figure 6B and D), hence the corrected alpha-level is 0.0023 which indicates that there was a high probability that the observed positive responses were the result of chance. The results are interesting but are not a sufficient basis for asserting a new paradigm for bark beetle-fungal interactions.  I think it can stay as-is.

We thank the reviewer for clarification and in fact, reviewer 3 also raised the same concern. We have now performed Bonferroni correction on all the bioassays shown in Figure 6B and D. However, we corrected the alpha level separately for each set of experiments focusing on a single monoterpene and not on all experiments as done by the reviewer as it risks increasing type II error. The adjusted significance level for 6B left panel (*trans*-4-thujanol) is α=0.007, 6B right panel (terpinen-4-ol) is α=0.012, and 6D (camphor) is α=0.012. The Bonferroni adjusted significance levels did not change our conclusion on the attraction of beetles to 100 μg *trans*-thujanol and 100 μg camphor as their P values are below Bonferroni adjusted significance levels. However, repulsion by 1 mg *trans*-4-thujanol and by 1 mg terpinen-4-ol is no longer significant. Thus, our data now demonstrate the attraction of walking beetles to *trans*-4-thujanol and camphor, and no effect with terpinen-4-ol and (+)-isopinocamphone and not repulsion as reported previously. We have now edited the Figure 6 title and the corresponding results section to account for these changes.

14)	My criticism:  The sum total of problems merits rejection.  Authors' Response:   We will revise the paper to address your concerns.  Reviewer response: I greatly appreciate the extensive work taken to address my concerns.

L23  Is 3-6 mm the true size range?

The size range reported in the recent literature is actually narrower; therefore, we changed the numbers.

L23  Insert "possible" before role.  The sentence implies the existence of such is already known or commonly hypothesized.

Actually, this is commonly hypothesized, although not in all references to *I. typographus* and other bark beetles, so we modified the text to “sometimes ascribed to two main factors…”.

L29-31  Change "We previously demonstrated…"  to "Evidence indicates…"

It has been changed although we left the word “previously” to ensure readers are aware that this result comes from past work and not the current study.

L29  The word "recognize" is too vague and suggests much more than the evidence support.  Be specific. (e.g.  "can distinguish cultures of").

It has been changed.

L29  There are no studies demonstrating that any of the fungi in this study were beneficial or not.  Statements to this effect must be removed.  They are symbionts of unknown ecological relationship. Remove "beneficial".

The reviewer is correct. The word “beneficial” has been deleted.

L35  One cannot say "converting" without experiments that would involve labelled precursor.  The oxygenated monoterpenes appear when the fungus is in the presence of resin monoterpenes.  That "conversion" is occurring is likely but not proven.  It is proven that hydrocarbon monoterpenes + fungal growth = appearance of oxygenated monoterpenes, but this is not a demonstration of metabolism, although it seems the most obvious hypothesis for the result.

Please see the response to the next point.

L34-38 The language here suggests that the pathway has been identified (generally one does not draw conclusions about metabolism without a pathway being demonstrated).  The wording should be changed to reflect that the data showed input of one compound resulted in the appearance of the other and no more than this.  De novo production has not been ruled out [Leufven et al (1988) found oxygenated monoterpenes appearing in their yeast cultures even though autoclaving of their media had appeared to have volatilized all hydrocarbon monoterpenes] although oxidation is the simplest explanation.

Another alternative explanation is that the fungi create an environment where spontaneous oxidation is more likely (e.g., by increasing exposure to oxygen or by providing surface area for oxidation to occur), although the appearance of species-specific blends is not consistent with these hypotheses.  Please state that the data "suggest", "imply" etc. a mechanism unless this mechanism has

been demonstrated explicitly.

On first glance, the metabolism of monoterpenes by *G. penicillata* would seem to be unproven without experiments tracing isotopic label from precursors to products. However, the observations we have made and the chemical properties of the products suggest that this bark beetle symbiotic fungus is indeed converting the supplied monoterpene hydrocarbons to oxygenated derivatives and de novo synthesis of oxygenated derivatives by the fungus can be ruled out.

In our previous publication (Kandasamy et al., 2019) we cultivated *G. penicillata* and the other fungi studied here under many conditions without spruce bark, spruce bark agar or supplemental monoterpenes. We detected many other volatile compounds, but never found any trace of oxygenated cyclic monoterpenes, like those with the pinane, thujane, bornane and *p*-menthane carbon skeletons reported here.

Moreover, fungi are only rarely reported to make cyclic monoterpenes, and there are no reports of compounds with thujane and bornane carbon skeletons. These are well-known in plants, especially conifers, but would be unprecedented in fungi. Thus, we conclude that the compounds we detected must be metabolites of host tree-derived compounds.

From previous reports on oxygenated monoterpenes and bark beetle-associated fungi, it seems clear that these are metabolites of host tree monoterpene hydrocarbons as well (Leufvén and Birgersson, 1987; Birgersson and Leufvén, 1988; Birgersson and Bergström, 1989; Pettersson and Boland, 2003). The exceptions, such as the claim that oxygenated monoterpenes are made *de novo* by yeast (Leufvén et al., 1988) seem hard to accept. Leufvén et al. state that autoclaving volatilized all monoterpene hydrocarbons, but in our repeated autoclaving of spruce bark we were never able to remove all monoterpenes. Thus, we are convinced that Leufvén et al. indeed had residual monoterpenes in their culture substrate, but were not able to detect them using the analytical methods available to them over 30 years ago.

We have now added these points to the discussion to support the fact that *G. penicillata* metabolizes spruce monoterpene hydrocarbons to oxygenated derivatives. Based on the strength of this case, we feel that the statements in the abstract can be left unqualified. Nevertheless, in the discussion we have admitted that we did not (yet) carry out experiments with isotopically-labeled traces- the typical “gold standard” for metabolic feeding studies. We thank the reviewer for her/his skepticism, which now prompted us to articulate these points in lines 369-399.

L38-39 There is NO evidence in the paper to support the statement that Trichoderma produced oxygenated monoterpenes in "non-attractive ratios"!  Trichoderma is likely non-beneficial but there is no evidence to this effect.  It is safe to say it is not a symbiont, or a non-symbiotic fungus that occupies the same habitat as the symbionts. I recommend the authors seek papers discussing Trichoderma's association with bark beetle killed trees.

We have reworded to emphasize that *Trichoderma* is not attractive to beetles, and we removed “non-attractive ratios” from this sentence.

L39 Delete "extensive"

It has been deleted.

L41 Delete "such as"; camphor and thujanol were the only ones.

It has been deleted.

L42 Change "olfactory" to "walking olfactometer".

It has been changed.

L47 Remove "essential" unless you can cite published papers that demonstrate that this is the case.  The ecological relationship is unknown, but could include mutualism, commensalism, and antagonism.  Since it is shown that oxygenated monoterpenes can be repellant to the bark beetles, I think it makes for a more interesting narrative to discuss the possibility that the different fungi producing the oxygenated monoterpenes could be deleterious or beneficial.  There is some mention of this in the discussion, but I would like this to be fully embraced by the paper.

The word “essential” has been removed from line 47.

L79 Delete comma and replace "which" with "that"

It has been changed.

L80  Replace "raises broods" with "reproduces"

It has been replaced.

L85-86 Change sentence.  The ecological relationships between these fungal species and I. typographus have not been extensively studied.

The sentence has been modified.

L88.  Add sentence "However, some ophiostamatoid associates of bark beetles have been shown to be commensals or antagonists to beetle brood development."

A new sentence has been added reflecting the antagonistic roles of ophiostomatoid fungi to bark beetles.

L98 Change "Interestingly," to "Additionally, "

It has been changed.

L103-105  Clarify this.  There are quite a few studies that have looked at the composition of resin within lesions on conifers following inoculation with bark beetle fungal associates.  Generally these studies didn't look for oxygenated monoterpenes, but the presence of the fungi did result in a change in volatile extractables from the phloem.

We thank the reviewer for this suggestion and have now added new text (L104-108) about our motivation to investigate oxygenated monoterpenes.

L106-107 Insert "excised" before "bark".

It has been added.

L108 Remove "dramatically"

It has been removed.

L111 Change "indicate" to "suggest" or "imply".  This statement requires a lot of untested assumptions, but it is a scenario that would explain the results.

It has been changed.

L121 Delete "much"

It has been deleted.

L123.  Explain "primary" and "secondary" in the introduction.  What does this mean and what evidence is used to make these classifications?

We deleted primary and secondary fungi as their classifications in the literature are not clear.

L124 Delete "highly"

It has been deleted.

L125  Identify Trichoderma as a possibly deleterious, non-symbiont and provide citations to support this.

We changed the sentence to “antagonistic non-symbiotic saprophyte”.

Table S2  GC-FID is not a sufficient means for identification when chromatograms are as complex as the ones in this study.  My experience is that a large amount of erroneous data is introduced into the literature when this is done, and the practice is not acceptable in chemical ecology journals.   The authors need to defend their approach here.

We ran the same sample through both GC-FID and GC-MS analyses using similar columns and identical temperature programs, and then overlaid the chromatograms. The GC-MS data was used to identify the compounds and the GC-FID data for quantification with reference to the internal standard. This approach has now been described more clearly in the methods section.

Figure S2.  Indicate the number of compounds used in the analysis.

It has been added.

L135 The figure/table references are unclear.

A few of these references have been removed for clarity.

L138 Delete parenthesis after "monoterpenes"

Deleted.

L141 Insert "for dates sampled"  after "reaching a maximum"

Inserted.

L142-143 Figure 2B suggests just the opposite, with the mean proportion of oxygenated monoterpenes increasing several-fold. Table S3 supports the statement.  You should give the statistics for the uninoculated treatment and mention something about the variance or in some way reconcile the text and the figure.  The P-value for G. penicillata seems strangely high (on the edge of significance) given the enormous difference in means (~10 fold).  Consider adding statistics for summed compounds of each class in tables S3-S7.

The pie charts in Fig 2B were made by calculating the relative amount of each compound class at each time point i.e., 5 % of the total volatile profile at 12 d in uninfected bark was represented by oxygenated monoterpenes. This does not mean that the total amount of oxygenated monoterpenes increased by 5-fold at 12 d when compared to 4 d or 8 d samples because the relative amount of oxygenated monoterpenes at 12 d depends on the total amount of other volatiles at this time. However, the text in lines 145-149 is based on the time series comparison of total amounts of oxygenated monoterpenes in uninfected bark and fungus-infected bark as listed in Tables S3-S6. To minimize confusion for other readers, we have now added more explanation to the legend for Fig. 2B.

We have added the summed compound statistics in tables S3-6. There was an error in reporting the P value for *G. penicillata*, which has been corrected now to 0.01 (L152)

L146-148.  Again, the data represented in figure 2B seem inconsistent with these stats.  In 2B, the change in proportion of hydrocarbon monoterpenes was only 7% whereas the P-value was very low.  Make a comment to reconcile this

Please see the comment above

Figure 2. Be consistent about placing the reference lettering either before or after the associated text.  Remove the sentence "Principal…treatment" as this information is apparent in the figure.

It has been removed.

L146 If the Tukey test results aren't reported here, delete "Tukey's test"

It has been deleted

L158 Move "(Fig…Table)" before "including"

It has been moved

L163  Normally "Fig" is written before the associated number/letter.  Be consistent about this.

Thanks for the reminder. The PLOS Biology format for citing supplementary figures is (Sx Fig). We are not used to this nomenclature either!

L166 Bornyl acetate is technically an "oxygenated monoterpene".   The authors restrict the use of "oxygenated monoterpene" to alcohols, ketones, and aldehydes.   Some comment on this limitation would be helpful.

The reviewer is correct that bornyl acetate is also an oxygenated monoterpene as it has an ester function. That’s why we listed this separately from monoterpene hydrocarbons in this title. Bornyl acetate like various monoterpene hydrocarbons is hydrolyzed and oxidized in the presence of the fungus to form other metabolites. We don’t want to restrict the use of “oxygenated monoterpene” to alcohols, ketones and aldehydes only because this is not chemically correct; esters should be included. Thus, we rephrased the heading to try to minimize confusion. Hopefully, it’s clearer now.

L173 Insert a comma after "similar"

It has been inserted.

L175 "included." Change "from" to "in the presence of"

It has been changed.

Table S2  GC-FID is not a sufficient means for identification when chromatograms are as complex as the ones in this study.  My experience is that a large amount of erroneous data is introduced into the literature when this is done, and the practice is not acceptable in chemical ecology journals.   The fact that large amounts of spiroketals (fairly rare compounds) were detected in large quantities in this FID study, but none or small amounts in the GC-MS studies, increases my skepticism.

The reviewer is correct that GC-FID is not sufficient for identification. We actually ran our samples for Table S2 on GC-MS for identification (carried out by calculating the sum formula from the molecular ion and comparing the fragmentation pattern and relative retention time with those in the literature) and on GC-FID for quantification. We have now revised the text in the Methods section and in the caption for the table to make this point clearer.

The fact that spiroketals show different intensities between GC-MS and GC-FID relative to terpenes, for example, is what might be expected given the very different chemical properties of the two classes. Total ion abundance in the GC-MS depends on the ability of a compound to ionize and its degree of fragmentation, whereas GC-FID abundance is relatively insensitive to the extent of fragmentation. Kudos to the reviewer for recognizing the differences in spiroketal abundance between these two methods! These results suggest that new attention should be given to these compounds because of their relative abundance, as well as their variability among samples.

L186 Remove the word "dramatically".

It has been removed.

L192-200.  Examination of an insect with electron microscopy as not compelling evidence of sterility.  There are many concealed locations on the exoskeleton of an insect where propagules could hide, and SEM obviously would not detect propagules carried internally. The plating data (Table S9) are the only meaningful evidence of removal of mycelial fungi.  Furthermore, figure 4A does not support the assertion that fungi were successfully reintroduced to the cuticle.  Figure Aii clearly shows classic "pillow shaped" ophiostamatoid sexual spores, whereas 4A  shows nothing that looks like a fungal propagule.  Hence, 4A provides no support (but does not contradict) the contention that fungi were successfully re-introduced, and should probably be removed.

We agree with the reviewer that scanning electron microscopy of beetle elytra should not be considered the only evidence for the sterility of beetles. However, since elytral pits are the main site of fungal transport in *I. typographus*, these micrographs help validate our sterility protocol. Together with plating data, they confirm that the fungi were successfully removed and reintroduced to the beetle. But, we agree that the plating data are more important, so since these are in the supplemental materials, we have also moved the micrographs there so that these are not overemphasized.

L206 Missing a parenthesis

It has been added.

203-206  In figure 4, reinoculated beetles were not significantly different from fungus free beetles. For terpinene-4-ol, there was no difference between no beetles and the reinoculated beetles.  Reconcile this.

The text has now been edited to correct this mistake. The figure is correct.

189-222  Despite the difficulties with re-inoculated beetles, this is still a very compelling experiment I think, and it is an outstanding contribution to the literature.

Thank you.

L271 The statement "within the range of natural concentrations in P. abies bark" requires support from experiments or citations.

Citations have now been added to support our statement.

L277-278  The most obvious explanation for the third test in 6A is that beta-pinene was causing the repellancy.  Why was it presumed that an oxygenated monoterpene was doing this?

Since we showed in Fig S11 that the addition of 1 mg/g of beta-pinene in bark agar did not result in the repulsion of beetles from the monoterpene-amended diet, we don’t think that the beta-pinene was the repellent in this case either, when it was presented with fungi. In Fig. 6A, on the other hand, we measured an increase in the production of oxygenated monoterpenes with increasing concentrations of beta-pinene in the diet, so these may be responsible for the repulsion observed (or at least the non-attraction to the fungus). High concentrations of the individual oxygenated beta-pinene metabolites, terpinen-4-ol and *trans*-4-thujanol and (+)-isopinocamphone we not attractive to the beetles. We have now revised the text to state that when spruce bark agar had a high concentration of beta-pinene, there was no significant choice with and without fungus, and attribute this to the presence of high levels of oxygenated monoterpenes.

L285-287  This conclusion is NOT supported by the results.  There is no evidence that the combination of beta-pinene and fungus is more attractive than fungus alone, and there is no evidence that repellency is caused by a metabolite of beta pinene and not beta-pinene itself.  There is evidence that specific oxygenated monoterpenes demonstrated elsewhere to be produced by the combination of fungi and host tissue can be either attractive or repellant to beetles.  This statement as well as the title of the section must be changed to reflect this.

We agree that we have not unequivocally demonstrated that fungal metabolites of beta-pinene are attractive to beetles, because we have not ruled out other fungal volatiles (Fig. 6A). Nonetheless, the correlation of attraction to beta-pinene metabolites provided an impetus to test the behavioral response of beetles to individual oxygenated monoterpenes Here the results show no net attraction to beta-pinene (Fig. S11) and the oxygenated monoterpene terpinen-4-ol and (+)-isopinocamphone at any concentration tested (Fig 6B), while there was attraction to *trans*-4-thujanol (Fig 6B) and camphor (Fig. 6D) at specific concentrations. . Hence the last sentence of the paragraph is actually correct. We have changed the title to indicate that oxygenated monoterpenes can attract or repel beetles.

L303-305 This statement belongs in the methods.

It has been moved to methods.

Figure 7.  The figure is a bit confusing because the compound combinations are indicated on the left hand side of the axis.  I am not sure how to rearrange this but, one gets the impression that the contrast is between the synthetic pheromone and the combinations.   Replacing "synthetic pheromone" with "pheromone components" would be helpful.

We edited the figure by moving compound combinations to the right side of the figure, and also replaced “synthetic pheromone” with “pheromone components”.

L310 Delete "significant"

Deleted.

L315  Consider deleting "success"

Also removed.

L320  The reference to units isn't helpful.  Just state the percentages.

We now state the percentages in the text and have deleted the units.

L315-338 These are good data, but tests of interactions between monoterpene presence and fungus would be informative regarding the hypothesis regarding the possible role of oxygenated monoterpenes.

We describe the significant interaction between beta-pinene and the fungus, and now state that no other significant interactions between fungus and monoterpenes were found to affect tunneling probability.

L337 figure 8B is the only evidence in the paper indicating that the combination of a terpene and fungus is more attractive than either singly, a finding supportive of the oxygenated monoterpene hypothesis.

L342 Entomocorticium are badiomycetes.

We have removed the word “ascomycete”.

L350 Data is presented that these compounds increased, but not they became dominant.

Based on quantification by GC-FID, oxygenated monoterpenes increased in our time course from 4% of total volatiles measured at 4 d to 49% at 12 d and 68% at 18 d (Fig. 2B, Table S2). We feel that this proportion (over two-thirds of total) merits the word “dominant”, but now also express this in a more quantitative way.

L352.  There is no evidence presented that oxygenated monoterpenes stimulate mining.

We have removed “and stimulate tunneling in” in the sentence.

L388-391. This cannot be concluded from the data in this paper.  The data presented do not demonstrate that oxygenated monoterpenes produced by fungal growth are attracting beetles to fungal cultures. There are no data presented on blends.  Only two compounds (one of the two - trans-4-thujanol - is produced by G. pinicillata in only minute amounts) were shown to be attractive singly, and no blends were tested.  Acceptance of the blends hypothesis would only be justified by tests with synthetic blends matching those produced by the fungi in which identical results were observed as with the fungi themselves. The fact that the oxygenated monoterpenes have many sources including non-symbionts is a problem for the hypothesis that oxygenated monoterpenes are used by beetles to distinguish beneficial fungi, no question, but to accept the blends hypothesis as the only possible solution is unjustified.  The role of oxygenated monoterpenes in allowing beetles to distinguish fungi has not been demonstrated definitively in this study, hence there is no urgency in resolving an inconsistency conflicting with this unproven hypothesis.  The blends hypothesis must be indicated as one possible explanation.  Another possible explanation (and the simpler one) is that oxygenated monoterpenes aren't what is allowing insects to distinguish fungi, and the variation in oxygenated monoterpene blends is irrelevant.  Relevant example: Healthy conifers typically have very specific blends of resin monoterpenes both among species and geographically, and many of these monoterpenes are attractants for bark beetles.  However, I am not aware of evidence that the beetles discriminate blends.  Is it acceptable to assume that, merely because there is variation in terpene blends associated with conifers, that bark beetles must be highly sensitive to blends, and there is no need to test this hypothesis?

Please see our response below.

L391-395  Since the blends hypothesis is not demonstrated or supported by tests of its validity, its existence as a known phenomenon cannot be used in the discussion.

We understand the objections of the reviewer. Oxygenated monoterpenes are correlated with the attraction of bark beetles, but of the four pure compounds tested, two were attractive and two had no effect. In a previous study, synthetic blends of de novo-produced fungal volatiles, including aliphatic and aromatic alcohols and esters, were attractive to beetles, but no synthetic blends of oxygenated monoterpenes were tested in this paper, and so the attractiveness of such blends is only a hypothesis. When it comes to tunneling, bark beetles tunnel more when host tree monoterpenes are added to diet in the presence of a fungus than in its absence. This is presumably due to fungal production of oxygenated monoterpenes, but only one of three host tree monoterpenes tested gave significant results. Thus, the thesis that oxygenated monoterpenes are critical fungal metabolites in bark beetle behavior is still tentative. We have now rewritten this section to be consistent with these points.

L407 "Metabolism" is perhaps too specific.  It could be a signal that defenses have been overcome or reduced, however the fungi accomplish this.

Good point. We have now added “or signal that tree defenses are overwhelmed or reduced” in line 460.

L416 Insert "could" before "provide"

Inserted.

L434 Delete "natural attacks upon"

Deleted.

L436 Change "within few days and" to "sufficiently rapidly to"

Changed.

L437-439  I don't see where it was demonstrated that specifically oxygenated monoterpenes increased tunnelling.  It is one possibility, and there are others.  Modify this statement accordingly.

We have now limited the statement by explaining the specific results on which it is based.

L440 Was there a reason to suspect there would be sex-specific differences?  Why was sex specificity only examined in the tunnelling and pheromone experiments?

Due to the distinct roles of male vs. female beetles in the *I. typographus* life cycle (males are pioneers, females follow later and colonize a tree in which host defenses are reduced, females construct galleries for egg laying) they could well respond differently to chemical cues that change during the invasion process. Thus, we did examine sex specificity in all of our bioassays, but only found it in the experiments with pheromone mixtures. We have now added this information to the text.

L451 Delete comma

Deleted.

L452 Verbenone can also be produced by autoxidation directly from alpha-pinene.

We agree with the reviewer, and we have now included this information in the text.

L454-455  I suggest deleting this sentence, since obviously structural differences produce different behaviors, and the same compound does not have a different effect at different stages of the beetle life cycle.

Deleted.

L457  Insert "natural" before "enemies"

Inserted.

L462-465  There is no evidence that this is mediated by oxygenated monoterpenes.

This sentence has been removed.

L456-471  Other relevant citations: Salom, S.M., Ascoli‐Christensen, A., Birgersson, G., Payne, T.L. and Berisford, C.W., 1992. Electroantennogram responses of the southern pine beetle parasitoid Coeloides pissodis (Ashmead)(Hym., Braconidae) to potential semiochemicals. Journal of Applied Entomology, 114(1‐5), pp.472-479.    Salom, S.M., Birgersson, G., Payne, T.L. and Berisford, C.W., 1991. Electroantennogram responses of the southern pine beetle parasitoid Dinotiscus dendroctoni (Ashmead)(Hymenoptera: Pteromalidae) to potential semiochemicals. Journal of chemical ecology, 17(12), pp.2527-2538.

L504-505 How did you identify it as a metabolite of alpha- and beta-pinene?

Elsewhere in the paper, we have now added further support to our claims that the oxygenated monoterpenes emitted by fungi growing on spruce bark or on medium supplemented with spruce monoterpenes are genuine fungal metabolites of host tree monoterpenes.

L506  Replace "another" with "an"

Replaced

L506-507 Provide citations

Citations have been added.

L522-524  I suppose exposing wounds to dead tissue of different fungal species (this can be sufficient to elicit a defensive response) might be a way to get at this.  There is evidence that feeding by certain insect herbivores elicit species-specific secondary volatiles from their hosts, and these blends have been hypothesized to attract specialist naturally enemies.  So it is not impossible that the tree might respond with specific blends.   You don't need to mention this…

This is an interesting proposal. We have now included a sentence (lines 571-573) to highlight the need for more research to ascertain or rule out the role of host tree as the source of oxygenated monoterpenes.

L559 What kind of insect is Xenopus?

*Xenopus* is a genus of African frogs and the oocytes of *X. laevis* are often used as a heterologous system to characterize insect odorant receptors. We have now described *Xenopus* in the text.

L566-569 Nice

L586.  There was no evidence presented that metabolism was how the compounds arise, only that the presence of fungi increased their concentrations.

We have now added more support for our claims of fungal metabolism elsewhere in the text.

L589 There was no evidence presented that oxygenated monoterpenes had any effect on beetle attraction to fungi or tunneling.  Clearly the fungi have an effect on attraction, but none of the experiments showed that fungal oxidation of monoterpenes played any role.

We have now revised these conclusions to avoid overinterpretation of our results.

L625 Were there steps to keep the phloem cold or otherwise from oxidation etc. prior to grinding?

We stored bark pieces immediately at -80C to avoid oxidation of the phloem. This information has now been added to the methods section.

L636-649  It doesn't seem that steps were taken to preserve the sterility of the collection bottles.  It seems very unlikely that incidental microbes didn't start to grow on the exposed tissue of the controls.  I think it would be better to say "uninoculated" rather than "uninfected" since the microbial state of the control plugs was unknown.

We apologize for our oversight in not mentioning the condition of the volatile collection jars. The bottles were sterile before they received samples. The reviewer is correct; we also noticed that some of the control bark had visible fungal growth after a few days of the experiment. We think the contaminants were endophytes from the bark and we have replaced “uninfected” with “uninoculated”.

L660-662  How were quantitations performed with the GC-MS?  Were standard curves produced?

The quantification was done using GC-FID data (not GC-MS) by comparing the peak area of unknown peaks to the peak area of an internal standard. The GC-FID peaks were identified by overlaying the chromatograms on those produced by the GC-MS since the same sample was analyzed in both GC-FID and GC-MS using identical columns and GC temperature programs. The text has now been rephrased to emphasize this information.

L674  I am not familiar with this method of volatile collection, but it appears to involve an adsorbent placed in a static headspace and the desorbed thermally.  Adsorbents tend to be selective and thus the proportions of compounds collected may not be representative of the proportion in the headspace.  Also, accurate quantitation is time consuming (requires calibration of the sampling device).  How was all of this addressed?

The reviewer is correct in her/his concerns, which were also addressed in the original reference [105]. We did not use this method for accurate quantification, but only to identify the compounds present with a sensitive detection method and obtain relative peak areas to compare among samples. We have now removed “quantified from” in line 740 to make it clear that this was not intended to be used for volatile quantification.

L693-694  It seems that the monoterpenes were added while the PDA was still hot enough to pour.  Was anything done to compensate for evaporation of the monoterpenes?  Was the 0.5 mg/ml likely to be accurate?

An excellent point. Addition of volatile, non-water soluble substances like monoterpenes to agar while achieving good dispersion is a challenge. We first dissolved monoterpenes in DMSO to increase their solubility in the water-based agar medium. These solutions were then added to the medium when the temperature of the agar was 57°C, mixed well and the medium poured onto plates. The warm temperature insures good mixing of monoterpenes in the agar, but risks loss of the compounds. Thus, we performed the bioassay immediately after pouring the plates without stopping to measure the final concentration of monoterpenes in the agar. Although no strong smell of volatile monoterpenes was noted, we did not assess losses due to the elevated temperature. We have now added these methodological details to the text.

L764-778   The construction of the olfactometer is not sufficiently clear from the text.   Figure 1A provides some information, but it needs to be in the text.  A detailed and labelled figure would be helpful.  Photographs in the supplementary materials would be helpful also.  It is not explained (1) How the beetles were able to get into the cups (there are holes evident in Fig 1A - how many, how large, and how high off the floor?  What material were the cups made of? Were these purchased or hand-constructed?  (2) What did the larger arena (containing the cups) consist of (material, diameter, height)? (3) Small holes are apparent in the walls of the larger arena in Fig 1A - what are the details of these and what was their purpose? (4) L268 states that two beetles were placed inside each arena - how many bioassays were done at one time? (5) Were the beetles actually "trapped" (couldn't escape) or were they merely inside the cups when they were examined?  (6) "For up to six

hours" - the cups were checked multiple times?  What determined when the assay was over?  How was each successive observation of the same arena included in the final data set? (7)  At what temperature and humidity were the assays conducted? (8) When the beetles were placed into the arena, where were they placed?  How were they released (with a forceps, etc.)? (9) Why two beetles per assay? (10) Were beetles re-used? (11) Were the arenas/cups re-used, and, if so, how were they cleaned between uses? (12) If known, what was the air flow rate in the laminar flow hood (high air flow could interrupt with semiochemical responses)? (13) Was the arena covered in some way?  (14) Was treatment assignment to cups randomized?

This information is all necessary to ensure that the bioassays described are repeatable

We appreciate the interest and detailed questions of the reviewer. The olfactometer used in this manuscript was described already in a previous publication (Kandasamy et al., 2019). Most of the reviewer's questions were answered in this previous publication, but missing information has now been added to the text in lines 823-839.

L825  It is not clear what "combined" means.

We have now clarified this in the text

L833-834 The behavioral bioassays were binary data—how was a Wilcoxon's test applied to these?

Actually, the behavioral assay data were not binary, and we converted beetle responses into percentages before using Wilcoxon’s test.

L834 "signed"

Corrected.

Reviewer #3:

This revision has some substantial improvements over the original version. In addition to some revised wording the authors conducted additional experiments that demonstrate specificity by the host insect toward fungal volatiles. That is very helpful. The revised manuscript does, however, require additional improvement in two areas.

First, as I pointed out in the original and Reviewer 2 also indicated, some of these experiments do not account for experiment-wise error. I appreciate and agree with the authors' response that their experimental design does not allow for such corrections, but that does not exempt them from the constraints on inference that such non-accounting impose. In particular, I do not see how general trends can be further delineated into responses purportedly going up, down, or both with specific doses without either fitting an overall dose-response curve or explicitly accounting for experiment-wise error. So those statements should be adjusted to what inferences can actually be made from the experimental design.

We have now performed Bonferroni correction on bioassay data shown in Fig 6B and D. The adjusted significance levels are: Fig. 6B left panel (*trans*-4-thujanol)- α=0.007, Fig. 6B right panel (terpinen-4-ol)- α=0.012, and Fig. 6D (camphor)- α=0.012. Bonferroni adjusted significance levels did not change our conclusion on the attraction of beetles to 100 μg *trans*-thujanol and 100 μg camphor as their P values are below Bonferroni adjusted significance levels. However, repulsion by 1 mg *trans*-4-thujanol and by 1 mg terpinen-4-ol is no longer significant. We have now edited the Figure 6 title and the corresponding results section to account for these changes.

Second, the authors now do a better job than before of relating these experiments to the natural condition of beetles arriving at fallen trees, but at times the language appears to encompass the rapid mass attacks by which these beetles overwhelm the defenses of healthy live trees. The experimental design and time frame are not applicable to the latter.

We agree with the reviewer that our experimental design was not suited for making any conclusions regarding the role of fungal volatiles during a rapid bark beetle attack. If attacks last at least a few days, this should give enough time for substantial fungal growth to occur and consequently for host tree monoterpenes to be metabolized, but this hypothesis is untested. We have now edited the text to state that the emission of fungal volatiles during rapid beetle attacks is uncertain and requires further research. (L489-490).

There are also a few remaining additional issues that can be easily corrected with minor rewording:

Line 88: Reference 20 is about bacteria, not fungi. So that needs to be stated separately rather than within a description of ectosymbiotic ophiostomatoid fungi.

Reference 20 has been deleted.

Line 402: It would be good to insert 'visual and chemical' in front of 'signals' to inform the reader that beetle orientation involves multiple behavioral modalities.

This has been inserted.

3.	Line 442: 'beneficial to both sexes'. This should be reworded to make more clear whether it refers to feeding by male and female adults or by larvae that develop into males or females.

This phrase has been reworded to indicate that feeding on fungus could be beneficial for both male and female adults.

4.	Line 495: 'Whether bark beetles, their associated fungi, or other microbes can detoxify diterpenes is not yet known' This was shown for bacteria in Boone et al. 2013.

We apologize for our oversight. This sentence has been removed now.

5.	Line 515. A useful reference to cite here is Hunt et al. 1989. They did observe some auto-oxidation of alpha-pinene, but in agreement with your findings it was very low and very delayed: only 0.8% and 1.9% of the trans-verbenol produced by D. ponderosae and D. rufipennis respectively, and over a time span too lengthy to significantly influence mass attack.

Hunt et al. 1989 has now been cited in the text.

6.	Line 580" What are 'higher' centers of the brain? Reword.

We have now described this term in more detail.
